# Prevalence of living alone with dementia and other progressive neurological conditions: findings from primary care data in England

**DOI:** 10.1186/s12916-025-04443-x

**Published:** 2025-11-04

**Authors:** Laura D. Gamble, Linda Clare, Anthony Martyr, Maria Caulfield, Catherine Charlwood, Claire Hulme, Jan R. Oyebode, Matthew Prina

**Affiliations:** 1 Population Health Sciences Institute, Newcastle University, Newcastle Upon Tyne, UK; 2 NIHR Policy Research Unit in Dementia and Neurodegeneration University of Exeter (DeNPRU Exeter) , Exeter, UK; 3 Department of Health and Community Sciences, University of Exeter Medical School, Exeter, UK; 4 NIHR Applied Research Collaboration South-West Peninsula, Exeter, UK; 5 Centre for Applied Dementia Studies, University of Bradford, Bradford, UK; 6 Wolfson Centre for Applied Health Research, Bradford, UK

**Keywords:** Alzheimer’s disease, Chronic neurological disorders, Residential status, Household composition, Solitary living, Social vulnerability, Socioeconomic disparities, Routine data, Population-based study

## Abstract

**Background:**

Living alone with a neurodegenerative condition such as dementia may result in inequalities in access to support and resources. However, estimates of the number of people living alone vary widely in research studies. We aimed to estimate the prevalence of living alone with dementia, Parkinson’s disease, motor neurone disease (MND), and Huntington’s disease in England using electronic healthcare records.

**Methods:**

Individuals identified in the Clinical Practice Research Datalink (CPRD) database were categorised as living alone or living with others based on medical codes and the number of people registered at a given GP practice from a household. Excluding those in care facilities, the prevalence of living alone was estimated both overall and stratified by measures of inequality including age, sex, ethnicity, deprivation, urban/rural location, and region. Prevalence estimates of the population living alone, applied to age and sex structures of upper local authority areas, were mapped. Change in prevalence over 15 years was investigated using Joinpoint analysis.

**Results:**

In 2023, there were 205,507 people with dementia in the CPRD database, 40,244 with Parkinson’s disease, 3515 with MND and 1672 with Huntington’s disease. Of those with dementia, 40% lived alone, 33% lived alone with Parkinson’s disease and MND and 38% lived alone with Huntington’s disease. More area-level deprivation and an urban GP practice were associated with living alone for all conditions. For dementia, Parkinson’s disease, and MND, there were differences by age, sex, ethnicity and region. The proportion of people living alone with dementia and MND has increased since 2009.

**Conclusions:**

A substantial number of people with neurodegenerative conditions are living alone, possibly exposing them to higher risks in some respect than carried by those who live with others. Changes to clinical policy are needed to enable improved support and targeted care for these individuals.

**Supplementary Information:**

The online version contains supplementary material available at 10.1186/s12916-025-04443-x.

## Background

Living alone in older age is becoming increasingly common, and currently about half of people aged 65 and older in the United Kingdom (UK) live alone [1]. As the population ages, the prevalence of conditions such as dementia and Parkinson’s disease is also increasing. According to the Alzheimer’s Society, there are currently an estimated 982,000 people living with dementia in the UK, with projections indicating this will rise to 1.4 million by 2040. With dementia and other neurodegenerative conditions affecting a significant proportion of older people, a substantial number are expected to live alone.

The four most prevalent neurodegenerative conditions are Alzheimer’s disease and other types of dementia, Parkinson’s disease, Huntington’s disease, and motor neurone disease (MND) [2], including amyotrophic lateral sclerosis or Lou Gehrig’s disease. In the UK, studies have estimated between 18 and 42% of people with dementia live alone [3–6]. Studies from other parts of the world vary widely, from 25 to 37% in the United States (US) and 23 to 62% in European countries [7]. For Parkinson’s disease, one global study found that just 12% lived alone [8], whereas some small studies in the UK indicate that 18–27% lived alone [9–12]. Estimates for other progressive neurological conditions, including MND and Huntington’s disease, are lacking.

Many of those living alone face complex health challenges. Despite the growing trend of older individuals living alone, little progress has been made in addressing the needs of people living alone with neurodegenerative conditions who likely face significant inequalities in accessing care. Studies suggest that people with dementia may struggle to engage with and access services, and may find services unresponsive [13], and that people with Parkinson’s disease find services difficult to access [14]. Additionally, living alone with these conditions has been shown to result in adverse outcomes such as poorer physical and psychological health, and poorer quality of life [9, 15, 16]. Several studies have shown that people living alone with dementia do not receive the same level of care, are more likely to be hospitalised, and are more likely to move into care than those living with others [3, 4, 15, 17–19].

Identifying how many people live alone with dementia and other neurodegenerative conditions, and who they are, is important for making policy recommendations aimed at increasing support and care. It is widely assumed that a family carer is available to provide support, and currently services designed to assist people living alone are often based on this assumption. As these populations grow, it is crucial to recognise that many of them potentially live without a carer.

There has been little to no research on the prevalence of living alone with MND or Huntington’s disease. This study aims to address this gap by estimating the prevalence of living alone with MND and Huntington’s disease, and to provide more robust estimates for dementia and Parkinson’s disease by using electronic healthcare records collected from GP practices in England. It also aims to find socio-demographic factors associated with living alone and to identify groups that may be particularly vulnerable.

## Methods

### Clinical Practice Research Datalink (CPRD) and data linkages

The CPRD contains routinely collected electronic health records from UK primary care practices [20, 21]. This study uses CPRD Aurum (September 2024 release), which contains anonymised records from almost 50 million research-acceptable patients [22]. CPRD data are generally representative of the UK population with respect to age, sex and ethnicity [20]. Primary care data in CPRD Aurum has been linked to national secondary care databases, including the Admitted Patient Care (APC) data from the Hospital Episodes Statistics (HES) datasets [23], and small-area level linkages based on practice or patients’ residence postcodes to obtain area-level deprivation (Index of Multiple Deprivation) and practice-level rural–urban classification [24].

### Study design and setting

We conducted a period prevalence study investigating living alone amongst people in England with a diagnosis of dementia, Parkinson’s disease, MND or Huntington’s disease. The main study period was the 2023 calendar year, but prevalence was also estimated at yearly intervals over 15 years (2009 to 2023).

### Study population

Patients with dementia, Parkinson’s disease, MND and Huntington’s disease were identified in CPRD by searching records for the medical codes and ICD-10 codes listed in Additional file 1: Tables S1–S8. The medical codes for these conditions were finalised by consultation with clinical experts. People with relevant ICD-10 codes were identified in linked HES APC data. When a code for a given condition appeared in clinical records, that patient was assumed to have a diagnosis from that point forward. A patient was included in the annual prevalence estimate if their registration start date was before or within the study period. A patient was excluded if their registration end date, practice last collection date, or death date occurred before the study period. Patients were also excluded if the living situation could not be determined.

A sensitivity analysis was conducted for dementia, Parkinson’s disease and MND that included people identified from prescribed medications specific to their condition (product codes for medications are listed in Additional file 1: Tables S9–S11).

### Defining living situation

Living situation is time-varying and was determined for each year from 2009 to 2023. Individuals that were alive and registered within each study year were classified, where possible, as either living alone, living with others, living in a care facility, or homeless. Care facilities included care/residential homes, hospices and long-term hospitals. Since living situation is not routinely recorded in clinical records, a procedure was followed:

1. Where available for a given year, living situation was determined from medical codes (see Additional file 1: Table S12) in the consultation and observation files. Source of consultation in CPRD and source of admission to hospital in HES APC data were also used to identify people in care facilities (see Additional file 1: Tables S13–S14).
2. Where living situation was missing for a given year, but a patient had a medical code indicating they were married or in a relationship, individuals were assumed to not live alone (see Additional file 1: Table S15).
3. Where living situation was still missing for a given year, the nearest available living alone status from surrounding years was used. If a code indicated someone lived in a care facility, then this was only assumed for future years and not previous years.
4. For any remaining missing living situations, where available, living alone status was inferred from the household number as previously described [25]. The household number is a unique ID given to individuals from the same household who are registered at a given GP practice. If two or more individuals were alive, registered, and from the same household, they were considered to live with others. If there was only one person from a household registered at a given GP practice, that individual was assumed to live alone. Household number does not have a time attached to it, so an assumption is made that this was recorded at the registration start date. We made a comparison of the proportion of individuals living alone 1) using medical codes only, 2) using household number to identify people living with others and 3) using household number to identify people living with others and people living alone (see Additional file 2: Table S1).

Using this approach, living situation was determined for the majority of people. Living situation was unable to be determined for just 0.7% of people with dementia, 1.2% of people with Parkinson’s disease, 1.7% of people with motor neurone disease and 1.2% of people with Huntington’s disease.

### Study measures

Age, sex, and region are recorded in the CPRD patient file. For dementia, age was categorised as < 65, 65–69, 70–74, 75–79, and 80 + years. For Parkinson’s disease, age was categorised as < 55, 55–64, 65–69, 70–74, 75–79, and 80 + years. For MND and Huntington’s disease, age was categorised as < 45, 45–54, 55–64, 65–74, and 75 + years. Regions included the North East, North West, Yorkshire and The Humber, East Midlands, West Midlands, East of England, South West, South East and London. The Index of Multiple Deprivation quintiles and GP practice’s urban/rural location were provided as small-area data linkages. Ethnicity was determined from HES APC data and from medical codes (see Additional file 1: Table S16). Ethnicity was categorised as white, black, Bangladeshi/Indian/Pakistani, other Asian, mixed, or other. Where there were inconsistencies in the recording of ethnicity between HES APC data and medical codes, the steps of the CPRD ethnicity algorithm were followed [26].

### Statistical analysis

Excluding those in a care facility and those who were homeless, a binary measure for living alone status was created (lives alone vs lives with others). Annual prevalence of living alone was estimated for each condition for the year 2023 (01/01/2023–31/12/2023). The analysis was repeated at yearly intervals going back 15 years (2009–2023). Annual prevalence was calculated by dividing the total number living alone by the population at risk of living alone. The population at risk of living alone was those who had both a diagnosis of the condition of interest and an available living alone status, were contributing acceptable quality data to CPRD during the study period (see Additional file 2: Additional methods for CPRD’s acceptable patient criteria), and were alive and registered within the study year.

For 2023, the prevalence of living alone was stratified by age, sex, ethnicity, region, deprivation quintiles, and urban/rural location where possible (a sample size > 5 is required to minimise reidentification risk). Log-binomial regression was used to explore differences in living situation (outcome) by each individual measure, adjusted for age band and sex (exposures). Risk ratios are reported alongside 95% confidence intervals. Reported prevalence estimates are unstandardised. Analyses were performed using R.

Only those with an available living situation (> 98% of people) were included in analyses. To account for missing data on ethnicity, deprivation, and urban/rural location, multiple imputation was conducted, generating 25 imputed datasets, which were combined according to Rubin’s rules.

### Maps of prevalence of living alone by local authority area in people with dementia and Parkinson’s disease

Logistic regression was used to predict the probability of each individual having dementia or Parkinson’s disease (outcome) by age, sex, and region (exposures). These estimates were applied to the age, sex and regional population distributions of England’s upper tier local authorities (counties or cities) using population data from July 2023 [27]. Multinomial regression was then used to predict the probability of these individuals either living alone, living with others, or living in a care facility (three-categorical outcomes), by age, sex and region (exposures). The predicted probabilities were applied to the estimated age, sex, and regional distribution of the population with each condition in each local authority to give standardised estimates of those living alone. Maps show deciles of the prevalence of living alone across the local authorities. Analyses were performed using R.

### Trends in living alone over time

Joinpoint regression identified changes in temporal trends in the prevalence of living alone, using the annual prevalence estimates across 15 years (2009–2023). Age and sex standardised rates were calculated for each year, using the age and sex distribution of the 2023 population for each condition. Joinpoint analyses were performed using Joinpoint Program 4.9.1.0 (available at https://surveillance.cancer.gov/joinpoint/).

## Results

### Prevalence of living alone with a neurodegenerative condition

In 2023, 205,507 people were living with dementia in the CPRD Aurum database, 40,244 were living with Parkinson’s disease, 3515 were living with MND and 1672 were living with Huntington’s disease.

Using the procedure outlined in the methods, living situation was defined for more than 98% of people with each condition; living situation was documented in clinical notes for 68% of people with dementia and for 38–48% of individuals with the other conditions, and where living situation was not documented in clinical notes, the number of people living in the same household was used to determine living situation (see Additional file 2: Table S1).

Excluding those categorised as living in a care facility (see Additional file 2: Table S2) or homeless, 40% of individuals with dementia lived alone, 33% with Parkinson’s disease lived alone, 33% with MND lived alone, and 38% with Huntington’s disease lived alone. These prevalences remained unchanged in the sensitivity analysis when prescribed medications were used to additionally identify people with these conditions (Additional file 2: Tables S3–S5).

### Prevalence stratified by measures of inequality

We looked at how those living alone and those living with others varied by factors including age, sex, region, ethnicity, deprivation level, and whether the GP practice was in an urban or rural area.

**Fig. 1:**
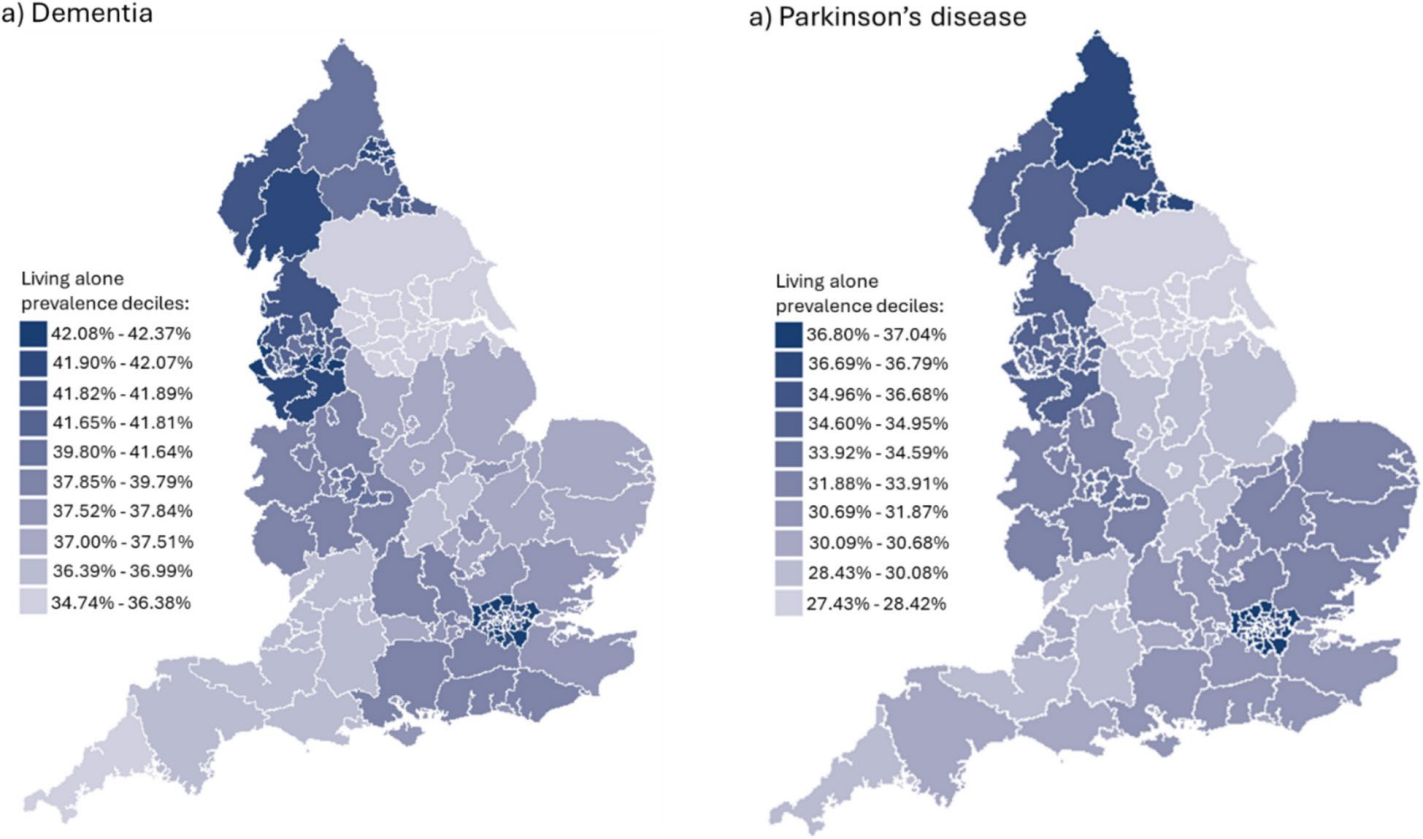
Estimated prevalence of living alone with **a** dementia and **b** Parkinson’s disease in England, by local authority area. The standardised prevalence rates of living alone, alongside 95% confidence intervals, are reported for each local authority area in Additional file 2: Table S6

Dementia was more prevalent in women than men (Table S2), and women were more likely to live alone (see Table 1). Those aged over 80 years were most likely to live alone, and there were also differences based on ethnicity; individuals of white ethnicity were more likely to live alone than those of any Asian ethnicity, while individuals of black ethnicity were more likely to live alone than both white and Asian groups. We also investigated regional differences. Individuals living in the North East and North West, London, and the West Midlands were most likely to live alone. Expanding this, we estimated standardised prevalence rates for the upper tier local authority areas across England, applied to local age and sex structures (Fig. 1a). The prevalence of living alone differs across the local authorities, with the highest prevalences in some of the local authorities in the North West, North East and London, and the lowest being in local authorities in Yorkshire and The Humber, and the South West. See Additional file 2: Table S6 for prevalence rates of all upper tier local authority areas.

**Table 1:** Prevalence of living alone with dementia stratified by measures of inequality

	Lives alone (*N*)	Lives with others (*N*)	Prevalence living alone % (95% CI)	Lives alone vs lives with others RR (95% CI)
Total	48,112	72,526	39.9% (39.6–40.2)	-
Sex				
Male	17,671	35,360	33.3% (32.9–33.7)	0.75 (0.74–0.76)
Female	30,441	37,166	45.0% (44.6–45.4)	Ref
Age				
80+	31,375	40,836	43.4% (43.1–43.8)	Ref
75-79	7239	14,133	33.9% (33.2–34.5)	0.79 (0.77–0.80)
70-74	3882	7539	34.0% (33.1–34.9)	0.79 (0.77–0.81)
65-69	2293	4179	35.4% (34.2–36.6)	0.83 (0.80–0.86)
<65	3323	5839	36.3% (35.3–37.3)	0.84 (0.81–0.86)
Region				
North East	1706	2313	42.4% (40.9–44.0)	1.11 (1.07–1.16)
North West	10,864	15,029	42.0% (41.3–42.6)	1.11 (1.09–1.14)
Yorkshire and The Humber	929	1722	35.0% (33.2–36.9)	0.93 (0.88–0.98)
East Midlands	759	1293	37.0% (34.9–39.1)	0.99 (0.94–1.05)
West Midlands	9224	13,916	39.9% (39.2–40.5)	1.05 (1.03–1.08)
East of England	1890	3110	37.8% (36.5–39.1)	1.00 (0.96–1.04)
London	8445	11,713	41.9% (41.2–42.6)	1.09 (1.06–1.11)
South East	9711	15,607	38.4% (37.8–39.0)	Ref
South West	4584	7823	36.9% (36.1–37.8)	0.97 (0.94–1.00)
Deprivation				
Quintile 1 (least)	7222	13,253	35.3% (34.6–35.9)	Ref
Quintile 2	7277	11,769	38.2% (37.5–39.9)	1.09 (1.06–1.11)
Quintile 3	6838	10,681	39.0% (38.3–39.8)	1.13 (1.10–1.16)
Quintile 4	7460	9932	42.9% (42.2–43.6)	1.23 (1.19–1.26)
Quintile 5 (most)	7126	8329	46.1% (45.3–46.9)	1.32 (1.29–1.36)
Missing	12,189	18,562		
Urban/rural				
Urban	35,305	51,476	40.7% (40.4–41.0)	Ref
Rural	4014	7714	34.2% (33.4–35.1)	0.84 (0.82–0.86)
Missing	8793	13,336		
Ethnicity				
White	42,450	61,653	40.8% (40.5–41.1)	Ref
Bangladeshi/Indian/Pakistani	1594	4929	24.4% (23.3–25.5)	0.62 (0.59–0.64)
Other Asian	502	1365	26.9% (24.9–28.9)	0.68 (0.63–0.73)
Black	2101	2315	47.6% (46.1–49.1)	1.13 (1.09–1.16)
Mixed	300	405	42.6% (38.9–46.2)	1.06 (0.97–1.15)
Other	597	1041	36.4% (34.1–38.8)	0.91 (0.85–0.97)
Missing	568	818		

*RR *risk ratio, *CI *confidence intervals. Analyses were adjusted for age band and sex.

Parkinson’s disease was more prevalent in men than women (Table S2), but women were more likely to live alone (Table 2). Similar patterns as for dementia were observed for age, ethnicity, and region. Estimated standardised prevalence rates applied to the local age and sex structure of the upper tier local authority areas are shown in Fig. 1b. Patterns are similar to those observed for dementia.

**Table 2:** Prevalence of living alone with Parkinson’s disease stratified by measures of inequality

	Lives alone (*N*)	Lives with others (*N*)	Prevalence living alone % (95% CI)	Lives alone vs lives with others RR (95% CI)
Total	11,048	22,286	33.1% (32.6–33.6)	-
Sex				
Male	6042	14,039	30.1% (29.5–30.7)	0.82 (0.78–0.85)
Female	5006	8247	37.9% (36.9–38.6)	Ref
Age				
80+	4399	7361	37.4% (36.5–38.3)	Ref
75–79	2366	5422	30.4% (29.4–31.4)	0.80 (0.77–0.82)
70–74	1711	3768	31.2% (30.0–32.5)	0.81 (0.78–0.85)
65–69	1117	2483	31.0% (29.5–32.5)	0.84 (0.80–0.88)
55–64	1151	2506	31.5% (30.0–33.0)	0.84 (0.80–0.87)
<55	304	746	29.0% (26.2–31.7)	0.77 (0.70–0.85)
Region				
North East	521	897	36.7% (34.2–39.3)	1.19 (1.10–1.28)
North West	2216	4174	34.7% (33.5–35.8)	1.12 (1.07–1.18)
Yorkshire and The Humber	222	581	27.6% (24.6–30.7)	0.90 (0.80–1.01)
East Midlands	166	407	29.0% (25.3–32.7)	0.94 (0.82–1.07)
West Midlands	2020	3924	34.0% (32.8–35.2)	1.09 (1.04–1.15)
East of England	485	1036	32.9% (29.5–34.2)	1.03 (0.95–1.12)
London	1742	2976	36.9% (35.5–38.3)	1.18 (1.13–1.24)
South East	2518	5614	31.0% (30.0–32.0)	Ref
South West	1158	2677	30.2% (28.7–31.6)	0.98 (0.92–1.04)
Deprivation				
Quintile 1 (least)	1920	4860	28.3% (27.2–29.4)	Ref
Quintile 2	1806	4090	30.6% (29.5–31.8)	1.11 (1.04–1.16)
Quintile 3	1659	3129	34.6% (33.3–36.0)	1.23 (1.17–1.30)
Quintile 4	1561	2593	37.6% (36.1–39.1)	1.35 (1.28–1.42)
Quintile 5 (most)	1291	1761	42.3% (40.5–44.1)	1.51 (1.43–1.58)
Missing	2811	5853		
Urban/rural				
Urban	7823	14,937	34.4% (33.8–35.0)	Ref
Rural	1195	3177	27.3% (26.0–28.7)	0.79 (0.76–0.84)
Missing	2030	4172		
Ethnicity				
White	10,023	19,862	33.5% (33.0–34.1)	Ref
Bangladeshi/Indian/Pakistani	259	985	20.8% (18.6–23.1)	0.63 (0.56–0.70)
Other Asian	136	424	24.3% (20.7–27.8)	0.73 (0.63–0.82)
Black	289	360	44.5% (40.7–48.4)	1.28 (1.19–1.40)
Mixed	57	110	34.1% (26.9–41.3)	1.04 (0.89–1.27)
Other	129	258	33.3% (28.6–38.0)	1.02 (0.63–1.17)
Missing	155	287		

*RR *risk ratio, *CI *confidence intervals. Analyses were adjusted for age band and sex.

MND was more prevalent in men, and whilst a sex difference in prevalence of living alone was less evident, a greater proportion of men lived alone than women, and younger individuals (< 45 years old) were least likely to live alone (Table 3). Regionally, those in the North West, Midlands and London were more likely to live alone. For ethnicity, those of Bangladeshi/Indian/Pakistani ethnicity were less likely to live alone than those of white ethnicity, whilst those of black or other ethnicity were most likely to live alone.

**Table 3:** Prevalence of living alone with motor neurone disease stratified by measures of inequality

	Lives alone (*N*)	Lives with others (*N*)	Prevalence living alone % (95% CI)	Lives alone vs lives with others RR (95% CI)
Total	975	1981	33.0% (31.3–34.7)	-
Sex				
Male	605	1156	34.4% (32.1–36.6)	1.10 (0.99–1.22)
Female	370	825	31.0% (28.3–33.6)	Ref
Age				
75+	232	434	34.8% (31.2–38.5)	Ref
65–74	215	445	32.6% (29.0–36.2)	0.94 (0.80–1.09)
55–64	209	372	36.0% (32.1–39.9)	1.04 (0.89–1.21)
45–54	124	237	34.3% (29.5–39.2)	0.99 (0.83–1.19)
<45	195	493	28.3% (25.0–31.7)	0.82 (0.70–0.96)
Region				
North East	40	83	32.5% (24.2–40.8)	1.16 (0.87–1.54)
North West	200	378	34.6% (30.7–38.5)	1.23 (1.05–1.45)
Yorkshire and The Humber	24	58	29.3% (19.4–39.1)	1.03 (0.72–1.48)
Midlands^a^	228	367	38.3% (34.4–42.2)	1.38 (1.18–1.61)
East of England	31	91	25.4% (17.7–33.1)	0.91 (0.66–1.26)
London	187	329	36.2% (32.1–40.4)	1.34 (1.13–1.58)
South East	192	491	28.1% (24.7–31.5)	Ref
South West	73	184	28.4% (22.9–33.9)	1.01 (0.80–1.27)
Deprivation				
Quintile 1 (least)	111	351	24.0% (20.1–27.9)	Ref
Quintile 2	124	311	28.5% (24.3–32.7)	1.19 (0.96–1.46)
Quintile 3	134	292	31.5% (27.0–35.9)	1.33 (1.07–1.66)
Quintile 4	164	257	39.0% (34.3–43.6)	1.63 (1.32–2.01)
Quintile 5 (most)	150	243	38.2% (33.4–43.0)	1.68 (1.36–2.08)
Missing	292	527		
Urban/rural				
Urban	717	1380	34.2% (32.2–36.2)	Ref
Rural	72	261	21.6% (17.2–26.0)	0.63 (0.51–0.79)
Missing	186	340		
Ethnicity				
White	810	1648	33.0% (31.1–34.8)	Ref
Bangladeshi/Indian/Pakistani	39	132	22.8% (16.5–29.1)	0.73 (0.55–0.97)
Other Asian	15	58	20.5% (11.3–29.8)	0.66 (0.42–1.04)
Black	49	52	48.5% (38.8–58.3)	1.54 (1.25–1.90)
Mixed	16	26	38.1% (23.4–52.8)	1.25 (0.85–1.85)
Other	28	34	45.2% (32.8–57.5)	1.45 (1.11–1.91)
Missing	18	31		

*RR *risk ratio, *CI *confidence intervals. Analyses were adjusted for age band and sex.

^a^East Midlands and West Midlands have been combined due to small numbers, in order to minimise re-identification risk.

Huntington’s disease was less common, and the smaller sample size meant less capability to detect differences. The prevalence of Huntington’s disease was similar in men and women (Table S2), and there was no difference in the prevalence of living alone detected by age or sex (Table 4). We were not able to explore differences by region or ethnicity.

**Table 4:** Prevalence of living alone with Huntington’s disease stratified by measures of inequality

	Lives alone (*N*)	Lives with others (*N*)	Prevalence living alone % (95% CI)	Lives alone vs lives with others RR (95% CI)
Total	442	718	38.1% (35.3–40.9)	-
Sex				
Male	218	345	38.7% (34.7–42.7)	1.04 (0.90–1.21)
Female	224	373	37.5% (33.6–41.4)	Ref
Age				
75+	51	82	38.3% (30.1–46.6)	Ref
65–74	71	133	34.8% (28.3–41.3)	0.91 (0.68–1.21)
55–64	113	191	37.2% (31.7–42.6)	0.97 (0.75–1.26)
45–54	105	145	42.0% (35.9–48.1)	1.10 (0.85–1.43)
<45	102	167	37.9% (32.1–43.7)	0.99 (0.76–1.29)
Deprivation				
Quintile 1 (least)	43	123	25.9% (19.2–32.6)	Ref
Quintile 2	56	92	37.8% (30.0–45.7)	1.78 (1.10–2.90)
Quintile 3	58	116	33.3% (26.3–40.3)	1.47 (0.92–2.36)
Quintile 4	72	107	40.2% (33.0–47.4)	1.99 (1.25–3.17)
Quintile 5 (most)	91	83	52.3% (44.9–59.7)	3.19 (2.02–5.10)
Missing	122	197		
Urban/rural				
Urban	325	500	39.4% (36.1–42.7)	Ref
Rural	37	90	29.1% (21.2–37.0)	0.75 (0.57–0.98)
Missing	80	128		

*RR *risk ratio, *CI *confidence intervals. Analyses were adjusted for age band and sex.

For all four conditions, individuals from the more deprived areas were more likely to live alone compared to those from less deprived areas. Individuals registered at urban GP practices were also most likely to live alone.

Associations with the study measures and living alone were similar in the sensitivity analysis where prescribed medications were used to additionally identify people with dementia, Parkinson’s disease, and MND (Additional file 2: Tables S3–S5).

### Trends in prevalence over time

**Fig. 2:**
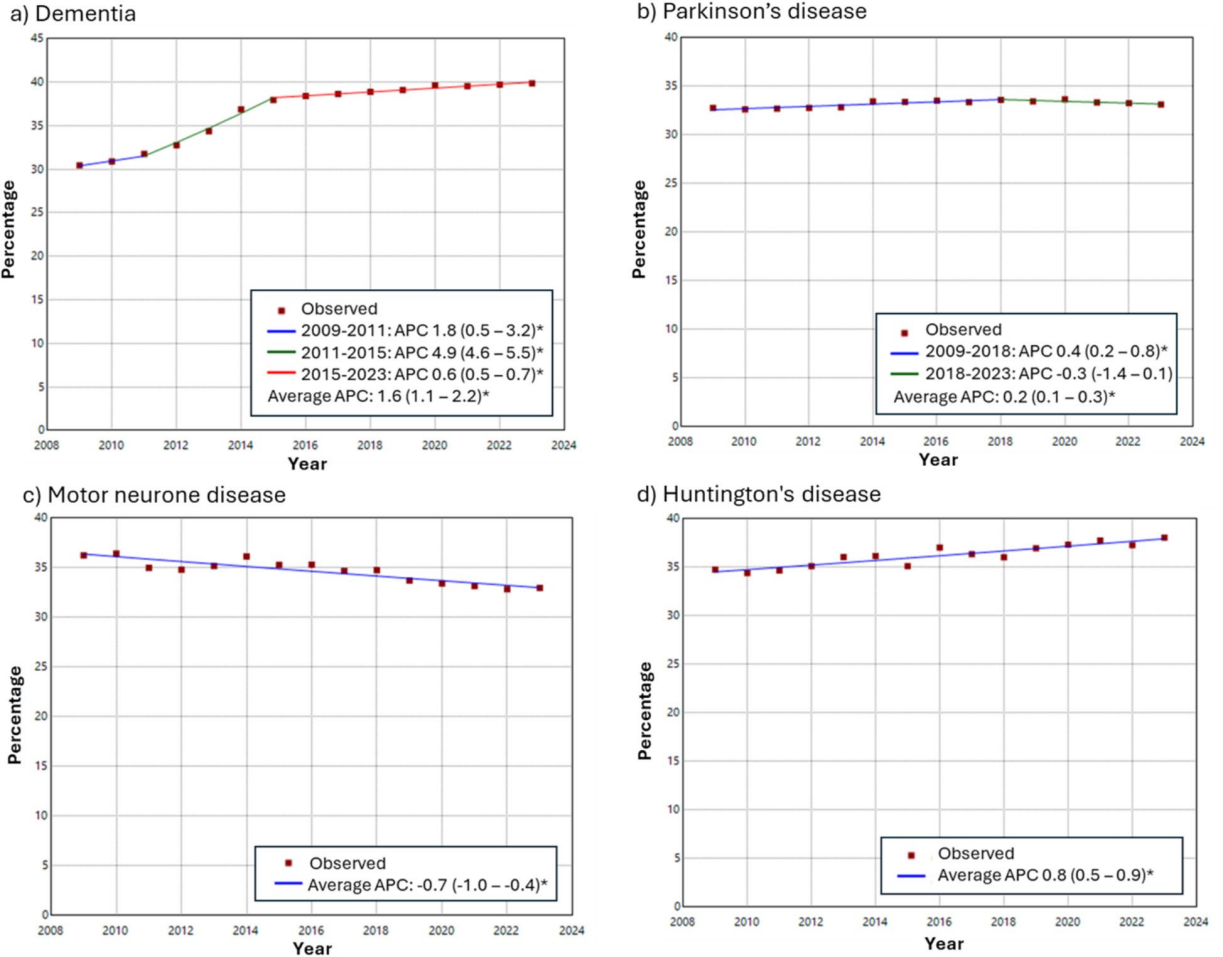
Trend over the past 15 years in prevalence of living alone with **a** dementia, **b** Parkinson’s disease, **c** motor neurone disease and **d** Huntington’s disease, using Joinpoint analysis

Over a period of 15 years, shifts are seen in the number of people recorded as living alone for some of the conditions (Fig. 2). The proportion of people living alone with dementia has risen from about 30% to 40%, and the proportion of people living alone with Huntington’s disease has increased from 35% to 38%. The proportion of individuals living alone with MND has declined slightly (36% to 33%), whereas the proportion of individuals living alone with Parkinson’s disease has remained relatively stable at around 33%.

## Discussion

Whilst living alone with a neurodegenerative condition, such as dementia, has been shown to lead to adverse outcomes, such as loneliness, depression and poorer quality of life, where available estimates of the number of people living alone vary widely. Using data from electronic healthcare records, we highlight that a substantial number of people are living alone with dementia, Parkinson’s disease, MND and Huntington’s disease in England. Certain demographic and social factors identify individuals more likely to live alone, such as being from a deprived area or attending an urban GP practice. With the exception of Huntington’s disease, there were also differences between those living alone and those living with others according to age, sex and ethnicity, and geographical variation according to English regions and localities.

We found that the majority of published studies focus on living alone with dementia, with few studies reporting on the other conditions [7, 28]. Several studies were based in the UK. A study using electronic health records from a London NHS Trust found that 23% of people with Alzheimer’s disease lived alone [3]. Of the community-dwelling individuals with dementia aged 50 + from the English Longitudinal Study of Ageing (ELSA), 42% lived alone [4]. A study in Northern Ireland reported that 35% of individuals taking dementia management medication lived alone, increasing to 42% excluding those in care homes [5]. Finally, of those admitted to a care home in Wales, 17.5% lived alone [6]. Few studies have reported on the proportion of people living alone with Parkinson’s disease, and it was not the aim of the study for those that have. Small studies in the UK suggest that 18–37% of people with Parkinson's disease lived alone when excluding those in care [9–12]. Differences in estimates are likely to vary widely due to the method of recruitment, method of data collection, how the condition is defined, exclusion/inclusion criteria and geographical location. For example, people living alone may be more difficult to recruit or have a reduced ability to participate in surveys, particularly when they have limited support. Studies that use an algorithm to detect dementia, such as ELSA, may pick up undiagnosed dementia, and studies only incorporating people with Alzheimer’s disease may have different population characteristics compared to studies incorporating all types of dementia. Differences due to geographical location will be influenced by cultural and economic differences, and also by societal expectations. Our estimate that 40% of individuals with dementia live alone, using data that is broadly representative of the UK in terms of age, sex and ethnicity, is in line with the upper estimates from the UK, as is our estimate of 33% for Parkinson’s disease. The only previous evidence for Huntington’s disease is from the Huntington’s Disease Association database [28] and supports the findings of this study that 38% live alone. There was no previous evidence of how many live alone with MND. This study brings recognition that there are a substantial number of people living alone with these neurological conditions.

Our findings are consistent with most other studies in dementia, where women and older people were more likely to live alone [17–19, 29, 30]. A study from the Netherlands highlights the age effect, with 21% of people under 70 years vs 70% of people over 90 years living alone [30]. For our age groupings, the difference was seen between < 80 and 80 + groups. Women and older people may be more likely to live alone due to being widowed, and other studies did indeed show that those who were not married or who were widowed were more likely to live alone [5, 29]. No studies have reported on age and sex in the other conditions, but our findings showed a similar pattern for Parkinson’s disease. The less pronounced age effect in MND, where those < 45 years old were less likely to live alone than those who are older, is likely because of life stage; there are fewer very old people who are likely to be widowed compared to dementia and Parkinson’s disease. There was no evidence of a difference for Huntington’s disease where the majority of people were younger than in the other conditions.

Only one previous US study has reported on ethnicity, finding no association with living alone with dementia [18]. Our findings for ethnicity were clear for all conditions except for Huntington’s disease, where numbers were insufficient; those of Asian ethnicity were most likely to live with others, whereas those of black ethnicity were most likely to live alone. People of ethnic minorities often experience higher levels of loneliness [31] and may experience inequality in accessing healthcare services [32, 33], and therefore individuals of black ethnicity may benefit from targeted interventions and support. South Asian groups are more likely to have traditional family carer roles and may more often live in multi-generation households, explaining why they are less likely to live alone [34]. For all conditions, living in more deprived areas was associated with living alone. This is consistent with findings from the Northern Ireland dementia study [5]. Other studies in dementia also found an association between lower income and living alone [18, 35]. These findings may be due to higher numbers of health conditions and greater health inequalities in deprived areas, and an increased chance of losing a spouse [36]. Additionally, those registered at urban GP practices were most likely to live alone, which is the opposite of what was found in the Northern Ireland study [5], and could be explained by the high levels of deprivation in cities such as London. We detected regional differences, and across the conditions, London consistently had higher prevalence rates of living alone along with the West Midlands and Northern regions. Understanding why older adults with these neurodegenerative conditions live alone involves a complex interplay of socioeconomic factors such as financial position, health, social networks and housing options.

Identifying how many and who live alone with dementia and other neurodegenerative conditions is important for support and provision planning. A comprehensive UK study demonstrated that people living alone with dementia have higher levels of unmet needs across multiple domains, and this leaves them particularly vulnerable to adverse outcomes [37]. A recent British study found that those living alone had more health inequalities such as poorer physical and psychological health, and poorer quality of life [15], and several studies have shown that this group does not receive the same level of care, are more likely to be hospitalised and are more likely to move into care [3, 4, 15, 17–19]. No studies have focussed specifically on living alone with Parkinson’s disease, but amongst studies that have included living alone as a factor, they reported increased risk of depression and poorer well-being, particularly if there was no family support and limited access to community resources for these individuals [9, 16, 38]. A single study in Huntington’s disease only looked at living alone in the context of suicide risk [39]. Given that neurodegenerative conditions have significant impacts on many aspects of a person’s life, some of which will be unique to the specific condition, those living alone may be vulnerable to adverse outcomes and poorer quality of life.

This study had a number of limitations. Living situation is not well recorded in clinical notes. Whilst we found this information for 68% of people with dementia, it was especially poor for the other conditions (38–48%). This led to us making several assumptions that may result in under- or over-estimations of the prevalence of living alone. If the living situation was not found in clinical notes, we made assumptions based on the number of people from the same household registered at a given GP practice, and this increased the availability of living situation to 98–99% of people. When two or more individuals from the same household were registered, those individuals were considered to not live alone. Whilst this assumption seems reasonable, it was also assumed that when only one person was registered, that person lived alone, and this may result in some overestimation. This may be the case for individuals living with working-age children, for example. However, if family number was considered for those living with others only, the number living with others would be inflated, and the prevalence of living alone with dementia would only be 17%, which is low compared to other studies. Other challenges of using electronic healthcare records include their longitudinal nature. Living situation was recorded at different times for different people, so a variable was created for each year, and the nearest living situation was taken when unavailable for a given year. This makes an assumption that living situation did not change in these instances. A key limitation of electronic healthcare records is the lack of sociodemographic information (e.g., education, income), which prevents adjustment for these factors. This may leave important confounders uncontrolled, increasing the risk of residual confounding. The findings that the prevalence of living alone with dementia and Huntington’s disease have increased over the past 15 years were expected, but the finding that the prevalence of MND decreased was not. The trends observed may be heavily influenced by changes in the recording of living alone status over time and also by increasing numbers of people going into care over the same timeframe.

## Conclusions

This study emphasises that there are substantial numbers of people living alone with neurodegenerative conditions, and this appears to be increasing for dementia and Huntington’s disease. It highlights the critical need for GPs and other service providers to routinely document the living situation of these individuals and to keep this information updated. Adaptations are needed to current systems to facilitate this, and this information needs to be easily available to the teams and individual practitioners involved. It is commonly assumed that individuals with dementia, for instance, always have a carer, yet neither health and social care services nor community resources are structured to meet the needs of those who live alone. Greater focus should be given to finding improved ways to support people living alone with dementia and other neurodegenerative conditions, and given the large number of affected individuals and the increasing ageing population, this should be a priority. This study also identifies sub-groups that may benefit from more proactive support. Targeted approaches are needed for higher-risk groups, such as those from more deprived areas or those of black ethnicity who may face unique challenges that require tailored solutions. Addressing these challenges, such as access to care, social isolation and financial hardship, could make a considerable difference to quality of life.

## Supplementary Information

Additional file 1. Table S1; Table S2, Table S3, Table S4, Table S5, Table S6, Table S7, Table S8, Table S9, Table S10. Table S11, Table S12, Table S13, Table S14, Table S15, Table S16. Table S1- Medical codes for dementia. Table S2- ICD-10 codes for dementia. Table S3- Medical codes to identify people with Parkinson’s disease. Table S4- ICD-10 codes to identify people with Parkinson’s disease. Table S5- Medical codes to identify people with Huntington’s disease. Table S6- ICD-10 codes to identify people with Huntington’s disease. Table S7- Medical codes to identify people with motor neurone disease. Table S8- ICD-10 codes to identify people with motor neurone disease. Table S9- Product codes for dementia medications. Table S10- Product codes for Parkinson’s disease medications. Table S11- Product codes for motor neurone disease medications. Table S12- Medical codes used to determine living situation (lives alone, does not live alone, living in a care facility or homeless). Table S13- EMIS® consultation source identifier (conssourceid in consultation files). Table S14- HES admission source codes (ADMISOURC – source of admission in HES APC). Table S15- Medical codes used to determine marital status. For those who were married or in a partnership, their living situation was assumed to be 'lives with others'. Table S16- Medical codes used to define ethnicity categories.

Additional file 2. Additional methods, Table S1, Table S2, Table S3, Table S4, Table S5, Table S6. Additional methods- Acceptable’ patient criteria in CPRD. Table S1- Exploring the use of both medical codes and household number to define those living alone. Table S2- A summary of the total numbers stratified by measures of inequality, and proportion living in a care home. Table S3- Sensitivity analysis: Prevalence of living alone with dementia stratified by measures of inequality, additionally including patients identified by condition-specific medications. Table S4- Sensitivity analysis: Prevalence of living alone with Parkinson’s disease stratified by measures of inequality, additionally including patients identified by condition-specific medications. Table S5- Sensitivity analysis: Prevalence of living alone with motor neurone disease stratified by measures of inequality, additionally including patients identified by condition-specific medications. Table S6- Age and sex standardised prevalence rates of living alone with dementia or Parkinson’s disease by each upper tier local authority area.

## Data Availability

Access to CPRD data is subject to a licence agreement and protocol approval via CPRD’s Research Data Governance (RDG) Process.

## Abbreviations

APC: Admitted Patient Care
CPRD: Clinical Practice Research Datalink
ELSA: English Longitudinal Study of Ageing
GP: General practitioner
HES: Hospital Episodes Statistics
ICD-10: International Classification of Diseases (10^th^ revision)
MND: Motor neurone disease
NHS: National Health Service
UK: United Kingdom
US: United States
