# Supplementary material for: Prevalence of living alone with dementia and other progressive neurological conditions: findings from primary care data in England
Source: BMC Med. 2025 Nov 4;23:607. doi: 10.1186/s12916-025-04443-x (PMC12584314; doi:10.1186/s12916-025-04443-x)
Supplement: Supplementary file 1 — Additional file 1. Table S1; Table S2, Table S3, Table S4, Table S5, Table S6, Table S7, Table S8, Table S9, Table S10. Table S11, Table S12, Table S13, Table S14, Table S15, Table S16. Table S1- Medical codes for dementia. Table S2- ICD-10 codes for dementia. Table S3- Medical codes to identify people with Parkinson’s disease. Table S4- ICD-10 codes to identify people with Parkinson’s disease. Table S5- Medical codes to identify people with Huntington’s disease. Table S6- ICD-10 codes to identify people with Huntington’s disease. Table S7- Medical codes to identify people with motor neurone disease. Table S8- ICD-10 codes to identify people with motor neurone disease. Table S9- Product codes for dementia medications. Table S10- Product codes for Parkinson’s disease medications. Table S11- Product codes for motor neurone disease medications. Table S12- Medical codes used to determine living situation (lives alone, does not live alone, living in a care facility or homeless). Table S13- EMIS® consultation source identifier (conssourceid in consultation files). Table S14- HES admission source codes (ADMISOURC – source of admission in HES APC). Table S15- Medical codes used to determine marital status. For those who were married or in a partnership, their living situation was assumed to be 'lives with others'. Table S16- Medical codes used to define ethnicity categories. [file 12916_2025_4443_MOESM1_ESM.docx]

## Additional file 1

## Table S1. Medical codes for dementia

| **MedCodeId** | **Term** |
| --- | --- |
| 914941000006112 | [D] Dementia |
| 914951000006114 | [D] Dementia in Alzheimer's disease |
| 914931000006119 | [D] Dementia with Lewy bodies |
| 914921000006117 | [D] Vascular dementia |
| 905791000006115 | [RFC] Alzheimer's disease |
| 939491000006118 | [RFC] Dementia |
| 359081000006118 | [X] Presenile dementia NOS |
| 359141000006111 | [X] Senile dementia NOS |
| 359151000006113 | [X] Senile dementia, depressed or paranoid type |
| 362941000006113 | [X]Alcoholic dementia NOS |
| 363021000006113 | [X]Alzheimer's dementia unspec |
| 363031000006111 | [X]Alzheimer's disease type 1 |
| 363041000006118 | [X]Alzheimer's disease type 2 |
| 363791000006112 | [X]Arteriosclerotic dementia |
| 295714013 | [X]Delirium superimposed on dementia |
| 295671015 | [X]Dementia in Alzheimer's dis, atypical or mixed type |
| 295668011 | [X]Dementia in Alzheimer's disease |
| 295672010 | [X]Dementia in Alzheimer's disease, unspecified |
| 376571000006116 | [X]Dementia in human immunodef virus [HIV] disease |
| 295690011 | [X]Dementia in other specified diseases classif elsewhere |
| 399031000006111 | [X]Multi-infarct dementia |
| 299325013 | [X]Other Alzheimer's disease |
| 295680015 | [X]Other vascular dementia |
| 423221000006117 | [X]Predominantly cortical dementia |
| 423351000006115 | [X]Presenile dementia, Alzheimer's type |
| 423381000006111 | [X]Primary degen dementia of Alzheimer's type, senile onset |
| 423391000006114 | [X]Primary degen dementia, Alzheimer's type, presenile onset |
| 425901000006116 | [X]Senile dementia, Alzheimer's type |
| 295681016 | [X]Vascular dementia, unspecified |
| 2931231000006118 | AD - Alzheimer's disease |
| 13909621000006119 | Aggression due to dementia |
| 13909641000006114 | Agitation due to dementia |
| 346929012 | Alcoholic dementia |
| 2502981000006117 | Alcohol-induced persisting dementia |
| 8030801000006118 | Altered behavior in Alzheimer's disease |
| 8024621000006116 | Altered behaviour co-occurrent and due to Pick's disease |
| 14514611000006117 | Altered behaviour due to Pick's disease |
| 8030791000006119 | Altered behaviour in Alzheimer's disease |
| 8024641000006111 | Altered behaviour in Huntington's dementia |
| 2931251000006113 | Alzheimer dementia |
| 2931241000006111 | Alzheimer disease |
| 15035271000006110 | Alzheimer disease with psychosis |
| 8044951000006115 | Alzheimers dementia with depressed mood |
| 3392901000006118 | Alzheimers dementia, late onset, with delusions |
| 45046017 | Alzheimer's disease |
| 8044981000006111 | Alzheimer's disease co-occurrent with delirium |
| 499946014 | Alzheimer's disease with early onset |
| 500317011 | Alzheimer's disease with late onset |
| 5005421000006117 | Amyotrophic lateral sclerosis with dementia |
| 15035231000006112 | Amyotrophic lateral sclerosis with frontotemporal dementia |
| 8028391000006119 | Anti-dementia drug therapy |
| 2290431000000110 | Antipsychotic drug therapy for dementia |
| 497559016 | Arteriosclerotic dementia |
| 294656010 | Arteriosclerotic dementia NOS |
| 294653019 | Arteriosclerotic dementia with delirium |
| 294655014 | Arteriosclerotic dementia with depression |
| 294654013 | Arteriosclerotic dementia with paranoia |
| 2284371000000110 | Assessment of psychotic and behavioural symptoms of dementia |
| 14464361000006117 | Autosomal dominant Alzheimer disease due to mutation of presenilin 1 |
| 12106391000006112 | Behavioral and psychological symptoms of dementia |
| 7755131000006117 | Behavioral variant of frontotemporal dementia |
| 12106351000006118 | Behavioural and psychological symptoms of dementia |
| 9896131000006110 | Behavioural disturbance co-occurrent and due to late onset Alzheimer dementia |
| 14805251000006118 | Behavioural disturbance due to multi-infarct dementia |
| 7755121000006115 | Behavioural variant of frontotemporal dementia |
| 149347010 | Binswanger's disease |
| 1235534016 | Binswanger's encephalopathy |
| 12106371000006111 | BPSD - behavioral and psychological symptoms of dementia |
| 12106381000006114 | BPSD - behavioural and psychological symptoms of dementia |
| 1576281000006119 | Cause of Death- Alzheimer's Disease |
| 297013012 | Cerebral degeneration due to cerebrovascular disease |
| 359101000006114 | Cerebral degeneration presenting primarily with dementia |
| 7559621000006112 | CHMP2B-related frontotemporal dementia |
| 3802671000006118 | Cortical Lewy body disease |
| 13934381000006118 | Cortical vascular dementia |
| 297000018 | Degenerative brain disorder |
| 12702281000006111 | Degenerative brain disorder |
| 7874761000006112 | Delirium co-occurrent with dementia |
| 7874771000006117 | Delirium superimposed on dementia |
| 8044911000006116 | Delusions in Alzheimer's disease |
| 359241000006119 | Dementia |
| 882171000006115 | Dementia |
| 2445471000000112 | Dementia advance care plan |
| 2345881000000115 | Dementia advance care plan agreed |
| 2743011000000119 | Dementia advance care plan agreed |
| 2345801000000113 | Dementia advance care plan declined |
| 2445691000000113 | Dementia advance care plan review declined |
| 12116721000006112 | Dementia advance care planning declined |
| 408401000000119 | Dementia annual review |
| 2451471000000113 | Dementia assessment care plan |
| 6973421000006116 | Dementia associated with AIDS |
| 2502971000006115 | Dementia associated with alcoholism |
| 295684012 | Dementia associated with another disease |
| 7510741000006111 | Dementia associated with multiple sclerosis |
| 7511961000006119 | Dementia associated with neurosyphilis |
| 7510731000006118 | Dementia associated with normal pressure hydrocephalus |
| 7043661000006117 | Dementia associated with Parkinson Disease |
| 7043651000006119 | Dementia associated with Parkinson's Disease |
| 7511971000006114 | Dementia associated with viral encephalitis |
| 2248021000000110 | Dementia care plan |
| 11930781000006119 | Dementia care plan |
| 2439591000000113 | Dementia care plan agreed |
| 2439671000000110 | Dementia care plan declined |
| 2439711000000111 | Dementia care plan review declined |
| 2439631000000113 | Dementia care plan reviewed |
| 7838831000006117 | Dementia caused by toxin |
| 1823871000006112 | Dementia confirmed |
| 7710381000006117 | Dementia co-occurrent with human immunodeficiency virus infection |
| 3636474012 | Dementia due to and following injury of head |
| 15039141000006114 | Dementia due to cerebral amyloid angiopathy |
| 15039111000006110 | Dementia due to cerebral vasculitis |
| 7838851000006112 | Dementia due to chromosomal anomaly |
| 7955111000006116 | Dementia due to chronic subdural haematoma |
| 14574821000006117 | Dementia due to cobalamin deficiency |
| 295686014 | Dementia due to Creutzfeldt Jakob disease |
| 7955131000006110 | Dementia due to herpes encephalitis |
| 295687017 | Dementia due to Huntington chorea |
| 7263021000006116 | Dementia due to Huntington’s disease |
| 13943421000006112 | Dementia due to iron deficiency |
| 8024671000006115 | Dementia due to multiple sclerosis with altered behaviour |
| 15041741000006111 | Dementia due to multiple system atrophy |
| 8031771000006112 | Dementia due to Parkinson disease |
| 8031761000006117 | Dementia due to Parkinsons disease |
| 8031751000006119 | Dementia due to Parkinson's disease |
| 8009531000006112 | Dementia due to Pick disease |
| 8009521000006114 | Dementia due to Picks disease |
| 295685013 | Dementia due to Pick's disease |
| 3636471016 | Dementia due to prion disease |
| 1972341000006111 | Dementia in Alzheimer's dis, atypical or mixed type, other mixed symptoms |
| 1972251000006119 | Dementia in Alzheimer's dis, atypical or mixed type, other symptoms, predominantly delusional |
| 1972311000006112 | Dementia in Alzheimer's dis, atypical or mixed type, other symptoms, predominantly depressive |
| 1972291000006113 | Dementia in Alzheimer's dis, atypical or mixed type, other symptoms, predominantly hallucinatory |
| 1972231000006114 | Dementia in Alzheimer's dis, atypical or mixed type, without additional symptoms |
| 6900241000006112 | Dementia in Alzheimer's disease - type 1 |
| 6897271000006114 | Dementia in Alzheimer's disease - type 2 |
| 376531000006119 | Dementia in Alzheimer's disease with early onset |
| 1972141000006113 | Dementia in Alzheimer's disease with early onset, other mixed symptoms |
| 1971541000006114 | Dementia in Alzheimer's disease with early onset, other symptoms, predominantly delusional |
| 1972131000006115 | Dementia in Alzheimer's disease with early onset, other symptoms, predominantly depressive |
| 1971771000006112 | Dementia in Alzheimer's disease with early onset, other symptoms, predominantly hallucinatory |
| 1971401000006111 | Dementia in Alzheimer's disease with early onset, without additional symptoms |
| 376541000006112 | Dementia in Alzheimer's disease with late onset |
| 1972211000006115 | Dementia in Alzheimer's disease with late onset, other mixed symptoms |
| 1972181000006119 | Dementia in Alzheimer's disease with late onset, other symptoms, predominantly delusional |
| 1972201000006118 | Dementia in Alzheimer's disease with late onset, other symptoms, predominantly depressive |
| 1972191000006116 | Dementia in Alzheimer's disease with late onset, other symptoms, predominantly hallucinatory |
| 1972171000006117 | Dementia in Alzheimer's disease with late onset, without additional symptoms |
| 1972471000006111 | Dementia in Alzheimer's disease, unspecified, other mixed symptoms |
| 1972401000006117 | Dementia in Alzheimer's disease, unspecified, other symptoms, predominantly delusional |
| 1972451000006118 | Dementia in Alzheimer's disease, unspecified, other symptoms, predominantly depressive |
| 1972371000006115 | Dementia in Alzheimer's disease, unspecified, without additional symptoms |
| 294718018 | Dementia in conditions EC |
| 295688010 | Dementia in Parkinsons disease |
| 1950791000006116 | Dementia link worker - mental health CPN |
| 1950811000006117 | Dementia link worker - mental health OT |
| 1950871000006114 | Dementia link worker - other |
| 1950821000006113 | Dementia link worker - other mental health worker |
| 1950851000006116 | Dementia link worker - other third sector worker |
| 1950861000006119 | Dementia link worker - social worker |
| 1950841000006118 | Dementia link worker - third sector (Alzheimer Scotland) |
| 6452391000006116 | Dementia management |
| 1916981000006111 | Dementia medication review |
| 2403101000000116 | Dementia medication review |
| 407461000000119 | Dementia monitoring |
| 406841000000115 | Dementia monitoring administration |
| 408361000000111 | Dementia monitoring first letter |
| 1856601000006111 | Dementia monitoring in primary care |
| 1856611000006114 | Dementia monitoring in secondary care |
| 8214731000006110 | Dementia monitoring invitation |
| 408421000000111 | Dementia monitoring second letter |
| 408501000000115 | Dementia monitoring telephone invitation |
| 408461000000115 | Dementia monitoring third letter |
| 408481000000112 | Dementia monitoring verbal invitation |
| 5618261000006114 | Dementia of frontal lobe type |
| 7969281000006110 | Dementia of the Alzheimer type with behavioural disturbance |
| 6897241000006118 | Dementia of the Alzheimers type with early onset |
| 6900201000006110 | Dementia of the Alzheimers type, late onset |
| 2575741000006113 | Dementia of the Alzheimer's type, with late onset, with delirium |
| 3802651000006111 | Dementia of the Lewy body type |
| 1950571000006111 | Dementia PDS (Scot) - peer support status |
| 1950501000006117 | Dementia PDS (Scot) - planning for future care status |
| 1950711000006114 | Dementia PDS (Scot) - planning future decision-making status |
| 1950641000006119 | Dementia PDS (Scot) - supporting community connections status |
| 1950651000006117 | Dementia PDS (Scot) support community connections - pillar met |
| 1950771000006117 | Dementia PDS plan future decision-making-pillar status not known |
| 1950551000006118 | Dementia PDS planning future care - other pillar status |
| 1950561000006116 | Dementia PDS planning future care - pillar status not known |
| 1950721000006118 | Dementia PDS planning future decision-making - pillar met |
| 1950731000006115 | Dementia PDS planning future decisn-making -pillar partially met |
| 1950661000006115 | Dementia PDS support community connections -pillar partially met |
| 1949761000006115 | Dementia PDS transition - referred for community care support |
| 1949721000006114 | Dementia post diagnostic support (PDS) - transition arrangements |
| 1949771000006110 | Dementia post diagnostic support (PDS)(Scot)- 5 pillar model |
| 1949611000006114 | Dementia post diagnostic support (Scotland) |
| 1949621000006118 | Dementia stage at diagnosis |
| 1949631000006115 | Dementia stage at diagnosis - early (mild) |
| 1949651000006110 | Dementia stage at diagnosis - late (severe) |
| 1949641000006113 | Dementia stage at diagnosis - mid (moderate) |
| 1949671000006117 | Dementia stage at diagnosis - not known |
| 1949661000006112 | Dementia stage at diagnosis - undetermined |
| 1949711000006118 | Dementia support plan (Scot) absent at 12 months |
| 1949691000006116 | Dementia support plan (Scot) in place at 12 months |
| 1949701000006116 | Dementia support plan (Scot) partly in place at 12 months |
| 14669521000006116 | Dementia with AIDS (acquired immunodeficiency syndrome) |
| 7969371000006115 | Dementia with behavioural disturbance |
| 7955161000006118 | Dementia with Down syndrome |
| 12370661000006114 | Dementia with mixed aetiology |
| 12370641000006110 | Dementia with mixed etiology |
| 14091281000006114 | Dementia with progressive multifocal leucoencephalopathy |
| 8044941000006117 | Depressed mood in Alzheimer's disease |
| 5618271000006119 | DFT - Dementia frontal lobe type |
| 2649061000006115 | Dialysis dementia |
| 299641000000112 | Diffuse Lewy body disease |
| 3189531000006118 | Diffuse Lewy body disease with spongiform cortical change |
| 13913061000006118 | Disinhibited behaviour due to dementia |
| 294688019 | Drug-induced dementia |
| 8032931000006117 | Early onset Alzheimers disease with behavioral disturbance |
| 8032911000006111 | Early onset Alzheimer's disease with behavioural disturbance |
| 8014401000006116 | Early onset dementia with delusions |
| 7863681000006115 | Epilepsy co-occurrent and due to dementia |
| 8024691000006119 | Epileptic dementia with behavioural disturbance |
| 5005501000006111 | Familial Alzheimer's disease of early onset |
| 5005541000006113 | Familial Alzheimer's disease of late onset |
| 5005581000006119 | Focal Alzheimer's disease |
| 15215821000006112 | Frontal variant non-amnestic Alzheimer disease |
| 345092016 | Frontotemporal degeneration |
| 5005601000006112 | Frontotemporal dementia |
| 7560531000006110 | Frontotemporal dementia with parkinsonism-17 |
| 7560411000006117 | Frontotemporal lobar degeneration |
| 2159232016 | GDS level 4 - moderate cognitive decline |
| 2159233014 | GDS level 5 - moderately severe cognitive decline |
| 2159234015 | GDS level 6 - severe cognitive decline |
| 2159235019 | GDS level 7 - very severe cognitive decline |
| 496157019 | General paralysis of insane |
| 3341631000006115 | General paresis |
| 496158012 | General paresis - neurosyphilis |
| 6749711000006110 | Global Deterioration Scale (GDS) level 4 - moderate cognitive decline |
| 6749741000006114 | Global Deterioration Scale (GDS) level 5 - moderately severe cognitive decline |
| 6749771000006118 | Global Deterioration Scale (GDS) level 6 - severe cognitive decline |
| 6749801000006116 | Global Deterioration Scale (GDS) level 7 - very severe cognitive decline |
| 251625013 | H/O: dementia |
| 7973121000006114 | Hallucinations co-occurrent and due to late onset dementia |
| 4539871000006116 | History of dementia |
| 3029421000006116 | Inhalant-induced persisting dementia |
| 7840631000006118 | Ischaemic vascular dementia |
| 3630121000006110 | Korsakoff psychosis |
| 394681000006119 | Korsakoff's psychosis |
| 753321000006116 | Korsakoff's syndrome - non-alcoholic |
| 3802641000006114 | LBD - Lewy body disease |
| 3195101000006116 | Lewy bodies |
| 3195111000006118 | Lewy body |
| 6024071000006116 | Lewy body dementia |
| 8042801000006115 | Lewy body dementia with behavioral disturbance |
| 8042791000006116 | Lewy body dementia with behavioural disturbance |
| 745381000006119 | Lewy body disease |
| 3802621000006119 | Lewy body variant of Alzheimer's disease |
| 7746341000006118 | Logopenic progressive aphasia |
| 3414231000006117 | MID - Multi-infarct dementia |
| 398571000006112 | Mixed cortical and subcortical vascular dementia |
| 1972931000006117 | Mixed cortical and subcortical vascular dementia, other mixed symptoms |
| 1972831000006110 | Mixed cortical and subcortical vascular dementia, other symptoms, predominantly delusional |
| 1972911000006111 | Mixed cortical and subcortical vascular dementia, other symptoms, predominantly depressive |
| 1972871000006113 | Mixed cortical and subcortical vascular dementia, other symptoms, predominantly hallucinatory |
| 1972821000006112 | Mixed cortical and subcortical vascular dementia, without additional symptoms |
| 8024201000006111 | Mixed dementia |
| 2288241000000118 | Moderate cognitive impairment |
| 3414261000006114 | Multi infarct dementia |
| 696161000006115 | Multi-infarct dementia |
| 8033241000006113 | Multi-infarct dementia due to atherosclerosis |
| 2664601000006115 | Multi-infarct dementia with delirium |
| 2912321000006113 | Multi-infarct dementia with delusions |
| 2722931000006114 | Multi-infarct dementia with depression |
| 1972681000006112 | Multi-infarct dementia, other mixed symptoms |
| 1972661000006119 | Multi-infarct dementia, other symptoms, predominantly depressive |
| 3653671000006113 | Multi-infarct dementia, uncomplicated |
| 12370651000006112 | MVAD - Mixed vascular Alzheimer dementia |
| 2773801000006117 | Non-alcoholic Korsakoff's psychosis |
| 5005691000006117 | Non-Alzheimer's progressive dysphasia |
| 7834071000006116 | Non-amnestic Alzheimer disease |
| 5005521000006118 | Non-familial Alzheimer's disease of early onset |
| 5005561000006112 | Non-familial Alzheimer's disease of late onset |
| 2742971000000119 | Offer of dementia advance care planning review declined |
| 3350441000006115 | Organic dementia |
| 401760017 | Other alcoholic dementia |
| 1973401000006112 | Other vascular dementia, other mixed symptoms |
| 1973271000006117 | Other vascular dementia, other symptoms, predominantly delusional |
| 1973381000006112 | Other vascular dementia, other symptoms, predominantly depressive |
| 1973341000006118 | Other vascular dementia, other symptoms, predominantly hallucinatory |
| 1973221000006118 | Other vascular dementia, without additional symptoms |
| 2733161000000117 | PAINAD (Pain Assessment in Advanced Dementia) Scale score |
| 3511181000006115 | Parkinson-dementia complex of Guam |
| 5005881000006116 | Patchy dementia |
| 1949681000006119 | Person centred dementia support plan (Scotland) |
| 2707661000006119 | Pick disease |
| 2707671000006114 | Picks disease |
| 22408016 | Pick's disease |
| 7734291000006113 | Posterior cortical atrophy |
| 7511421000006118 | Post-traumatic dementia with behavioural change |
| 2729771000000110 | Predominantly cortical dementia |
| 2729791000000114 | Predominantly cortical vascular dementia |
| 346897015 | Presbyophrenic psychosis |
| 21256010 | Presenile dementia |
| 6964691000006110 | Presenile dementia associated with AIDS |
| 294643015 | Presenile dementia NOS |
| 14669181000006111 | Presenile dementia with AIDS (acquired immunodeficiency syndrome) |
| 294638010 | Presenile dementia with delirium |
| 8014391000006118 | Presenile dementia with delusions |
| 294642013 | Presenile dementia with depression |
| 294641018 | Presenile dementia with paranoia |
| 2729731000000113 | Presenile dementia with psychosis |
| 6897251000006116 | Presenile dementia, Alzheimer's type |
| 8010111000006117 | Primary degenerative dementia |
| 6897211000006117 | Primary degenerative dementia of the Alzheimer type, presenile onset |
| 7515131000006110 | Primary degenerative dementia of the Alzheimer type, presenile onset in remission |
| 2603271000006110 | Primary degenerative dementia of the Alzheimer type, presenile onset, uncomplicated |
| 3558401000006117 | Primary degenerative dementia of the Alzheimer type, presenile onset, with delirium |
| 3384371000006119 | Primary degenerative dementia of the Alzheimer type, presenile onset, with delusions |
| 2667431000006115 | Primary degenerative dementia of the Alzheimer type, presenile onset, with depression |
| 6900181000006114 | Primary degenerative dementia of the Alzheimer type, senile onset |
| 7515121000006112 | Primary degenerative dementia of the Alzheimer type, senile onset in remission |
| 3575041000006117 | Primary degenerative dementia of the Alzheimer type, senile onset, uncomplicated |
| 2575731000006115 | Primary degenerative dementia of the Alzheimer type, senile onset, with delirium |
| 3392881000006115 | Primary degenerative dementia of the Alzheimer type, senile onset, with delusions |
| 2929931000006112 | Primary degenerative dementia of the Alzheimer type, senile onset, with depression |
| 7840661000006110 | Primary progressive aphasia |
| 5005681000006115 | Progressive aphasia |
| 5005711000006119 | Progressive aphasia in Alzheimer's disease |
| 7745171000006117 | Progressive non-fluent aphasia |
| 7843521000006113 | Rapidly progressive dementia |
| 1774581000006113 | Refer to dementia care advisor |
| 2405911000000115 | Referral to Alzheimer's Society |
| 1785381000000118 | Referral to dementia care advisor |
| 1933001000006115 | Referral to dementia early intervention service |
| 2221421000000114 | Referral to dementia service |
| 2373201000000112 | Referral to dementia support organisation |
| 2373161000000116 | Referral to dementia support organisation declined |
| 2345931000000111 | Review of dementia advance care plan |
| 2742991000000115 | Review of dementia advance care plan |
| 14479161000006110 | Review of dementia care plan |
| 7750421000006118 | Right temporal atrophy variant frontotemporal dementia |
| 2748441000006111 | SD - Senile dementia |
| 6900221000006117 | SDAT - Senile dementia, Alzheimer's type |
| 13810191000006114 | SDLT - senile dementia of Lewy body type |
| 5005871000006119 | Semantic dementia |
| 882191000006119 | Senile and presenile dementias |
| 76484016 | Senile degeneration of brain |
| 26545010 | Senile dementia |
| 882201000006116 | Senile dementia - simple type |
| 6024061000006111 | Senile dementia of the Lewy body type |
| 294648012 | Senile dementia with delirium |
| 6348031000006112 | Senile dementia with delusion |
| 294646011 | Senile dementia with depression |
| 294644014 | Senile dementia with depressive or paranoid features |
| 294647019 | Senile dementia with depressive or paranoid features NOS |
| 294645010 | Senile dementia with paranoia |
| 6348061000006115 | Senile dementia with psychosis |
| 882211000006118 | Senile dementia-acute confused |
| 148381000006115 | Senile/presenile dementia |
| 2288281000000114 | Severe cognitive impairment |
| 2620431000000115 | Shared care prescribing of drug for dementia |
| 2620471000000118 | Shared care prescribing of drugs for dementia declined |
| 7794741000006116 | Shared care prescribing protocol of drugs for dementia |
| 3964651000006112 | Subcortical arteriosclerotic encephalopathy |
| 3964661000006114 | Subcortical atherosclerotic dementia |
| 3635492010 | Subcortical dementia |
| 428201000006119 | Subcortical vascular dementia |
| 1972791000006115 | Subcortical vascular dementia, other mixed symptoms |
| 1972731000006119 | Subcortical vascular dementia, other symptoms, predominantly delusional |
| 1972771000006116 | Subcortical vascular dementia, other symptoms, predominantly depressive |
| 1972751000006114 | Subcortical vascular dementia, other symptoms, predominantly hallucinatory |
| 1972711000006113 | Subcortical vascular dementia, without additional symptoms |
| 294652012 | Uncomplicated arteriosclerotic dementia |
| 294637017 | Uncomplicated presenile dementia |
| 294635013 | Uncomplicated senile dementia |
| 1972081000006115 | Unspecified dementia, other mixed symptoms |
| 1972041000006114 | Unspecified dementia, other symptoms, predominantly delusional |
| 1972071000006118 | Unspecified dementia, other symptoms, predominantly depressive |
| 1972061000006113 | Unspecified dementia, other symptoms, predominantly hallucinatory |
| 1972021000006119 | Unspecified dementia, without additional symptoms |
| 3414251000006112 | VAD - Vascular dementia |
| 431681000006117 | Vascular dementia |
| 431691000006119 | Vascular dementia of acute onset |
| 1972571000006110 | Vascular dementia of acute onset, other mixed symptoms |
| 1972501000006116 | Vascular dementia of acute onset, other symptoms, predominantly delusional |
| 1972481000006114 | Vascular dementia of acute onset, without additional symptoms |
| 8089241000006117 | Vascular dementia with behavioral disturbance |
| 8089231000006110 | Vascular dementia with behavioural disturbance |
| 9902251000006117 | Vascular dementia without behavioural disturbance |
| 1976831000006111 | Vascular dementia, unspecified, other mixed symptoms |
| 1973501000006113 | Vascular dementia, unspecified, other symptoms, predominantly delusional |
| 1973551000006112 | Vascular dementia, unspecified, other symptoms, predominantly depressive |
| 1973531000006117 | Vascular dementia, unspecified, other symptoms, predominantly hallucinatory |
| 1973461000006113 | Vascular dementia, unspecified, without additional symptoms |
| 2664611000006117 | Vascular dementia, with delirium |
| 2912331000006111 | Vascular dementia, with delusions |
| 3630091000006111 | Wernicke-Korsakoff syndrome |

## Table S2. ICD-10 codes for dementia

| F00.0 | Dementia in Alzheimer disease with early onset |
| --- | --- |
| F00.1 | Dementia in Alzheimer disease with late onset |
| F00·2 | Dementia in Alzheimer disease, atypical or mixed type |
| F00·9 | Dementia in Alzheimer disease, unspecified |
| F01·0 | Vascular dementia of acute onset |
| F01·1 | Multi-infarct dementia |
| F01·2 | Subcortical vascular dementia |
| F01·3 | Mixed cortical and subcortical vascular dementia |
| F01·8 | Other vascular dementia |
| F01·9 | Vascular dementia, unspecified |
| F02·0 | Dementia in Pick disease |
| F02·1 | Dementia in Creutzfeldt-Jakob disease |
| F02·2 | Dementia in Huntington disease |
| F02·3 | Dementia in Parkinson disease |
| F02·4 | Dementia in human immunodeficiency virus [HIV] disease |
| F02·8 | Dementia in other diseases classified elsewhere |
| F03 | Unspecified dementia |
| F05·1 | Delirium superimposed on dementia |
| G30·0 | Alzheimer disease with early onset |
| G30.1 | Alzheimer disease with late onset |
| G30.8 | Other Alzheimer disease |
| G30.9 | Alzheimer disease, unspecified |
| G31.0 | Circumscribed brain atrophy (Frontotemporal dementia (FTD), Pick disease, Progressive isolated aphasia) |
| G31.1 | Senile degeneration of the brain, not elsewhere classified |
| I67.3 | Progressive vascular leukoencephalopathy (binswanger’s disease) |

## Table S3. Medical codes to identify people with Parkinson’s disease

| **MedCodeId** | **Term** |
| --- | --- |
| 905821000006112 | [RFC] Parkinson's disease |
| 909021000006117 | [RFC] Parkinson's disease |
| 1013231000006115 | Adverse reaction to Anticholinergic Drugs Used In Parkinsonism |
| 363531000006115 | Adverse reaction to antiparkinsonism drug |
| 465071000006115 | Adverse reaction to antiparkinsonism drugs |
| 331018011 | Adverse reaction to antiparkinsonism drugs NOS |
| 1013221000006118 | Adverse reaction to Dopaminergic Drugs Used In Parkinsonism |
| 1013211000006114 | Adverse reaction to Drugs Used In Parkinsonism And Related Disorders |
| 636681000000117 | Cerebral degeneration in Parkinson's disease |
| 7043661000006117 | Dementia associated with Parkinson Disease |
| 7043651000006119 | Dementia associated with Parkinson's Disease |
| 8031771000006112 | Dementia due to Parkinson disease |
| 8031761000006117 | Dementia due to Parkinsons disease |
| 8031751000006119 | Dementia due to Parkinson's disease |
| 295688010 | Dementia in Parkinsons disease |
| 3028161000006113 | Disorders presenting primarily with parkinsonism |
| 474251000000115 | Dopaminergic drug used in parkinsonism adverse reaction |
| 7730811000006111 | Early onset Parkinson disease |
| 7730821000006115 | Early onset Parkinson's disease |
| 1148731000000112 | History of Parkinson's disease |
| 3293681000006117 | Idiopathic Parkinsonism |
| 3293631000006118 | Idiopathic Parkinson's disease |
| 402514015 | O/E - Parkinson gait |
| 280121000006113 | O/E - Parkinson posture |
| 254935018 | O/E - Parkinsonian tremor |
| 254944017 | O/E -Parkinson flexion posture |
| 402512016 | O/E-festination-Parkinson gait |
| 297043014 | Orthostatic hypotension co-occurrent and due to Parkinson's disease |
| 81719014 | Paralysis agitans |
| 3293641000006111 | Parkinson disease |
| 5254231000006117 | Parkinsonian facies |
| 5930421000006111 | Parkinsonian features |
| 5528051000006115 | Parkinsonian flexion posture |
| 5279871000006117 | Parkinsonian gait |
| 5983301000006110 | Parkinsonian tremor |
| 3293661000006110 | Parkinsons disease |
| 81717011 | Parkinson's disease |
| 297037012 | Parkinson's disease NOS |
| 3293651000006113 | PD - Parkinson's disease |
| 48512013 | Progressive supranuclear ophthalmoplegia |
| 48513015 | Progressive supranuclear palsy |
| 297258013 | Progressive supranuclear palsy |
| 14463991000006112 | Progressive supranuclear palsy parkinsonism syndrome |
| 3511377010 | PSP - progressive supranuclear palsy |
| 7790241000006117 | Psychosis co-occurrent and due to Parkinson's disease |
| 1776901000006117 | Reason for referral: Parkinsons Disease |
| 1863311000006117 | Referral by Parkinsons disease nurse specialist |
| 1925611000006114 | Referral to community Parkinson's disease clinical nurse specialist |
| 7730791000006112 | Young onset Parkinson disease |
| 7730801000006113 | Young onset Parkinson's disease |

## Table S4. ICD-10 codes to identify people with Parkinson’s disease

| G20 | Parkinson's disease, including hemiparkinsonism, idiopathic Parkinsonism, paralysis agitans, and primary Parkinsonism |
| --- | --- |
| G23.1 | progressive supranuclear ophthalmoplegia |

## Table S5. Medical codes to identify people with Huntington’s disease

| **MedCodeId** | **Term** |
| --- | --- |
| 97642017 | Huntington's chorea |
| 295687017 | Dementia due to Huntington chorea |
| 5006031000006119 | Late onset Huntington's disease |
| 3454551000006117 | Huntington chorea |
| 5006041000006112 | Late onset Huntington disease |
| 14195861000006111 | Huntington disease |
| 8024641000006111 | Altered behaviour in Huntington's dementia |
| 7263021000006116 | Dementia due to Huntingtons disease |

## Table S6. ICD-10 codes to identify people with Huntington’s disease

| F02.2 | Dementia in Huntington disease |
| --- | --- |
| G10 | Huntington's disease |

## Table S7. Medical codes to identify people with motor neurone disease

| **MedCodeId** | **Term** |
| --- | --- |
| 905801000006119 | [RFC] Motor neurone disease |
| 908821000006116 | [RFC] Motor neurone disease |
| 3898661000006113 | ALS - Amyotrophic lateral sclerosis |
| 142653015 | Amyotrophic lateral sclerosis |
| 351861000000110 | Amyotrophic lateral sclerosis drugs Band 1 |
| 5005421000006117 | Amyotrophic lateral sclerosis with dementia |
| 15035231000006112 | Amyotrophic lateral sclerosis with frontotemporal dementia |
| 142016013 | Anterior horn cell disease |
| 297132016 | Anterior horn cell disease NOS |
| 3898641000006114 | Bulbar motor neuron disease |
| 3101721000006110 | MND - Motor neurone disease |
| 3007701000006115 | Motor neuron |
| 3101701000006117 | Motor neuron disease |
| 486695013 | Motor neurone disease |
| 297130012 | Motor neurone disease NOS |
| 297131011 | Other anterior horn cell disease |
| 134744017 | Primary lateral sclerosis |
| 90263012 | Progressive bulbar palsy |
| 5277731000006115 | Progressive pseudobulbar palsy |

## Table S8. ICD-10 codes to identify people with motor neurone disease

| G12.2 | Motor neuron disease |
| --- | --- |

## Table S9. Product codes for dementia medications

| **ProdCodeId** | **Product name** | **Drug substance name** |
| --- | --- | --- |
| 1609241000033116 | Exelon 1.5mg capsules | Rivastigmine hydrogen tartrate |
| 1609341000033114 | Exelon 3mg capsules | Rivastigmine hydrogen tartrate |
| 1609441000033115 | Exelon 4.5mg capsules | Rivastigmine hydrogen tartrate |
| 1609541000033119 | Exelon 6mg capsules | Rivastigmine hydrogen tartrate |
| 1673341000033115 | Rivastigmine 1.5mg capsules | Rivastigmine hydrogen tartrate |
| 1673441000033114 | Rivastigmine 3mg capsules | Rivastigmine hydrogen tartrate |
| 1673541000033110 | Rivastigmine 4.5mg capsules | Rivastigmine hydrogen tartrate |
| 1673641000033111 | Rivastigmine 6mg capsules | Rivastigmine hydrogen tartrate |
| 2656441000033116 | Rivastigmine 2mg/ml oral solution sugar free | Rivastigmine hydrogen tartrate |
| 2656541000033115 | Exelon 2mg/ml oral solution | Rivastigmine hydrogen tartrate |
| 4321241000033114 | Rivastigmine 4.6mg/24hours transdermal patches | Rivastigmine |
| 4321541000033111 | Rivastigmine 9.5mg/24hours transdermal patches | Rivastigmine |
| 4322041000033111 | Exelon 4.6mg/24hours transdermal patches | Rivastigmine |
| 4322641000033117 | Exelon 9.5mg/24hours transdermal patches | Rivastigmine |
| 6469441000033118 | Nimvastid 1.5mg capsules | Rivastigmine hydrogen tartrate |
| 6469641000033116 | Nimvastid 4.5mg capsules | Rivastigmine hydrogen tartrate |
| 6469741000033113 | Nimvastid 6mg capsules | Rivastigmine hydrogen tartrate |
| 8271941000033112 | Kerstipon 6mg capsules | Rivastigmine hydrogen tartrate |
| 8348041000033112 | Rivastigmine 13.3mg/24hours transdermal patches | Rivastigmine |
| 8348141000033111 | Exelon 13.3mg/24hours transdermal patches | Rivastigmine |
| 8349341000033119 | Voleze 4.6mg/24hours transdermal patches | Rivastigmine |
| 8349441000033113 | Voleze 9.5mg/24hours transdermal patches | Rivastigmine |
| 9073441000033113 | Erastig 4.6mg/24hours transdermal patches | Rivastigmine |
| 9073541000033114 | Erastig 9.5mg/24hours transdermal patches | Rivastigmine |
| 9165441000033111 | Alzest 4.6mg/24hours transdermal patches | Rivastigmine |
| 9165541000033112 | Alzest 9.5mg/24hours transdermal patches | Rivastigmine |
| 9366241000033112 | Somniton 4.6mg/24hours transdermal patches | Rivastigmine |
| 9366841000033111 | Somniton 9.5mg/24hours transdermal patches | Rivastigmine |
| 9411841000033112 | Prometax 4.6mg/24hours transdermal patches | Rivastigmine |
| 9412041000033110 | Prometax 9.5mg/24hours transdermal patches | Rivastigmine |
| 10642641000033112 | Erastig 13.3mg/24hours transdermal patches | Rivastigmine |
| 10674541000033119 | Eluden 4.6mg/24hours transdermal patches | Rivastigmine |
| 10674641000033118 | Eluden 9.5mg/24hours transdermal patches | Rivastigmine |
| 11403041000033111 | Voleze 13.3mg/24hours transdermal patches | Rivastigmine |
| 12198541000033112 | Almuriva 4.6mg/24hours transdermal patches | Rivastigmine |
| 12198741000033116 | Almuriva 9.5mg/24hours transdermal patches | Rivastigmine |
| 13812141000033117 | Alzest 13.3mg/24hours transdermal patches | Rivastigmine |
| 14116041000033112 | Almuriva 13.3mg/24hours transdermal patches | Rivastigmine |
| 14242041000033117 | Zeyzelf 4.6mg/24hours twice weekly transdermal patches | Rivastigmine |
| 14242141000033118 | Zeyzelf 9.5mg/24hours twice weekly transdermal patches | Rivastigmine |
| 2191341000033112 | Galantamine 4mg tablets | Galantamine hydrobromide |
| 2191441000033118 | Galantamine 8mg tablets | Galantamine hydrobromide |
| 2191541000033117 | Galantamine 12mg tablets | Galantamine hydrobromide |
| 2801441000033112 | Galantamine 20mg/5ml oral solution sugar free | Galantamine hydrobromide |
| 3293541000033114 | Galantamine 8mg modified-release capsules | Galantamine hydrobromide |
| 3293641000033110 | Galantamine 16mg modified-release capsules | Galantamine hydrobromide |
| 3293741000033118 | Galantamine 24mg modified-release capsules | Galantamine hydrobromide |
| 2191641000033116 | Reminyl 4mg tablets | Galantamine hydrobromide |
| 2191741000033113 | Reminyl 8mg tablets | Galantamine hydrobromide |
| 2191841000033115 | Reminyl 12mg tablets | Galantamine hydrobromide |
| 2801541000033113 | Reminyl 4mg/ml oral solution | Galantamine hydrobromide |
| 3293841000033111 | Reminyl XL 8mg capsules | Galantamine hydrobromide |
| 3293941000033115 | Reminyl XL 16mg capsules | Galantamine hydrobromide |
| 3294041000033118 | Reminyl XL 24mg capsules | Galantamine hydrobromide |
| 6435841000033110 | Galsya XL 8mg capsules | Galantamine hydrobromide |
| 6435941000033119 | Galsya XL 16mg capsules | Galantamine hydrobromide |
| 6436041000033112 | Galsya XL 24mg capsules | Galantamine hydrobromide |
| 6529241000033116 | Zeebral XL 8mg capsules | Galantamine hydrobromide |
| 6529341000033114 | Zeebral XL 16mg capsules | Galantamine hydrobromide |
| 6529441000033115 | Zeebral XL 24mg capsules | Galantamine hydrobromide |
| 7690141000033115 | Acumor XL 24mg capsules | Galantamine hydrobromide |
| 7690441000033111 | Acumor XL 16mg capsules | Galantamine hydrobromide |
| 7690641000033113 | Acumor XL 8mg capsules | Galantamine hydrobromide |
| 8127241000033112 | Gatalin XL 8mg capsules | Galantamine hydrobromide |
| 8127341000033119 | Gatalin XL 16mg capsules | Galantamine hydrobromide |
| 8127441000033113 | Gatalin XL 24mg capsules | Galantamine hydrobromide |
| 8242941000033118 | Lotprosin XL 8mg capsules | Galantamine hydrobromide |
| 8243041000033111 | Lotprosin XL 16mg capsules | Galantamine hydrobromide |
| 8243141000033110 | Lotprosin XL 24mg capsules | Galantamine hydrobromide |
| 8305441000033119 | Elmino XL 16mg capsules | Galantamine hydrobromide |
| 8306941000033118 | Elmino XL 24mg capsules | Galantamine hydrobromide |
| 9679141000033112 | Luventa XL 24mg capsules | Galantamine hydrobromide |
| 9679241000033117 | Luventa XL 8mg capsules | Galantamine hydrobromide |
| 9679341000033110 | Luventa XL 16mg capsules | Galantamine hydrobromide |
| 9699241000033110 | Gazylan XL 16mg capsules | Galantamine hydrobromide |
| 9699441000033111 | Gazylan XL 24mg capsules | Galantamine hydrobromide |
| 9699541000033112 | Gazylan XL 8mg capsules | Galantamine hydrobromide |
| 10217341000033112 | Consion XL 8mg capsules | Galantamine hydrobromide |
| 10217441000033118 | Consion XL 16mg capsules | Galantamine hydrobromide |
| 10217541000033117 | Consion XL 24mg capsules | Galantamine hydrobromide |
| 11825741000033113 | Galzemic XL 8mg capsules | Galantamine hydrobromide |
| 11825841000033115 | Galzemic XL 16mg capsules | Galantamine hydrobromide |
| 11825941000033111 | Galzemic XL 24mg capsules | Galantamine hydrobromide |
| 12603841000033115 | Galzemic 4mg/ml oral solution | Galantamine hydrobromide |
| 12640941000033113 | Gaalin 16mg modified-release capsules | Galantamine hydrobromide |
| 12641041000033115 | Gaalin 24mg modified-release capsules | Galantamine hydrobromide |
| 12641141000033116 | Gaalin 8mg modified-release capsules | Galantamine hydrobromide |
| 81041000033119 | Aricept 10mg tablets | Donepezil hydrochloride |
| 81141000033115 | Aricept 5mg tablets | Donepezil hydrochloride |
| 479941000033116 | Donepezil 10mg tablets | Donepezil hydrochloride |
| 480041000033117 | Donepezil 5mg tablets | Donepezil hydrochloride |
| 4022041000033110 | Donepezil 5mg orodispersible tablets sugar free | Donepezil hydrochloride |
| 4022141000033114 | Donepezil 10mg orodispersible tablets sugar free | Donepezil hydrochloride |
| 4022241000033119 | Aricept Evess 5mg orodispersible tablets | Donepezil hydrochloride |
| 4022341000033112 | Aricept Evess 10mg orodispersible tablets | Donepezil hydrochloride |
| 9302241000033114 | Donepezil 1mg/ml oral solution sugar free | Donepezil hydrochloride |
| 14265641000033111 | Donepezil 10mg orodispersible tablets | Donepezil hydrochloride |
| 2835341000033112 | Memantine 10mg tablets | Memantine hydrochloride |
| 2835441000033118 | Memantine 10mg/ml oral solution sugar free | Memantine hydrochloride |
| 4607841000033119 | Memantine 20mg tablets | Memantine hydrochloride |
| 11492741000033118 | Memantine 10mg orodispersible tablets sugar free | Memantine hydrochloride |
| 11492841000033111 | Memantine 20mg orodispersible tablets sugar free | Memantine hydrochloride |
| 12331641000033115 | Memantine 10mg soluble tablets sugar free | Memantine hydrochloride |
| 12331741000033112 | Memantine 20mg soluble tablets sugar free | Memantine hydrochloride |
| 12532241000033110 | Memantine 5mg/10mg/15mg/20mg tablets treatment initiation pack | Memantine hydrochloride |
| 13316541000033110 | Memantine 5mg/10mg/15mg/20mg orodispersible tablets initiation pack sugar free | Memantine hydrochloride |
| 14274841000033116 | Memantine 5mg orodispersible tablets sugar free | Memantine hydrochloride |
| 2835541000033117 | Ebixa 10mg tablets | Memantine hydrochloride |
| 2835641000033116 | Ebixa 5mg/0.5ml pump actuation oral solution | Memantine hydrochloride |
| 4607941000033110 | Ebixa 20mg tablets | Memantine hydrochloride |
| 8535641000033114 | Nemdatine 10mg tablets | Memantine hydrochloride |
| 8535741000033117 | Nemdatine 20mg tablets | Memantine hydrochloride |
| 11492941000033115 | Valios 10mg orodispersible tablets sugar free | Memantine hydrochloride |
| 11493041000033113 | Valios 20mg orodispersible tablets sugar free | Memantine hydrochloride |
| 11720941000033116 | Marixino 20mg tablets | Memantine hydrochloride |
| 11721041000033114 | Marixino 10mg tablets | Memantine hydrochloride |
| 12331841000033119 | Alzhok 10mg soluble tablets | Memantine hydrochloride |
| 12331941000033110 | Alzhok 20mg soluble tablets | Memantine hydrochloride |
| 14274941000033112 | Valios 5mg orodispersible tablets sugar free | Memantine hydrochloride |

##

## Table S10. Product codes for Parkinson’s disease medications

| **ProdCodeId** | **Product name** | **Drug substance name** | |
| --- | --- | --- | --- |
| 820241000033112 | Levodopa Capsules 125 mg | Levodopa | |
| 820341000033119 | Levodopa Capsules 250 mg | Levodopa |  |
| 820441000033113 | Levodopa Capsules 500 mg | Levodopa | |
| 858141000033118 | Madopar Cr 125 Capsules | Benserazide hydrochloride/ Levodopa | |
| 871041000033114 | Madopar Dispersible Tablets | Benserazide hydrochloride/ Levodopa | |
| 4074441000033111 | Caramet 25mg/100mg CR tablets | Carbidopa monohydrate/ Levodopa | |
| 4074541000033112 | Caramet 50mg/200mg CR tablets | Carbidopa monohydrate/ Levodopa | |
| 6431141000033119 | Carbidopa 25mg tablets | Carbidopa | |
| 295941000033110 | Co-beneldopa 12.5mg/50mg capsules | Benserazide hydrochloride/ Levodopa | |
| 318241000033118 | Co-beneldopa 12.5mg/50mg dispersible tablets sugar free | Benserazide hydrochloride/ Levodopa | |
| 296041000033117 | Co-beneldopa 25mg/100mg capsules | Benserazide hydrochloride/ Levodopa | |
| 317641000033116 | Co-beneldopa 25mg/100mg dispersible tablets sugar free | Benserazide hydrochloride/ Levodopa | |
| 359341000033113 | Co-beneldopa 25mg/100mg modified-release capsules | Benserazide hydrochloride/ Levodopa | |
| 296141000033118 | Co-beneldopa 50mg/200mg capsules | Benserazide hydrochloride/ Levodopa | |
| 369341000033115 | Co-careldopa 10mg/100mg tablets | Carbidopa monohydrate/ Levodopa | |
| 176041000033111 | Co-careldopa 12.5mg/50mg tablets | Carbidopa monohydrate/ Levodopa | |
| 5150741000033113 | Co-careldopa 12.5mg/50mg/5ml oral suspension | Carbidopa monohydrate/ Levodopa | |
| 336841000033110 | Co-careldopa 25mg/100mg modified-release tablets | Carbidopa monohydrate/ Levodopa | |
| 369641000033111 | Co-careldopa 25mg/100mg tablets | Carbidopa monohydrate/ Levodopa | |
| 6001141000033116 | Co-careldopa 25mg/100mg/5ml oral solution | Carbidopa monohydrate/ Levodopa | |
| 4426441000033113 | Co-careldopa 25mg/100mg/5ml oral suspension | Carbidopa monohydrate/ Levodopa | |
| 369441000033114 | Co-careldopa 25mg/250mg tablets | Carbidopa monohydrate/ Levodopa | |
| 294641000033119 | Co-careldopa 50mg/200mg modified-release tablets | Carbidopa monohydrate/ Levodopa | |
| 3909841000033110 | Co-careldopa 5mg/20mg/1ml intestinal gel 100ml cassette | Carbidopa monohydrate/ Levodopa | |
| 6116041000033118 | Co-careldopa 6.25mg/25mg/5ml oral suspension | Carbidopa monohydrate/ Levodopa | |
| 3909941000033119 | Duodopa intestinal gel 100ml cassette | Carbidopa monohydrate/ Levodopa | |
| 660841000033113 | Half Sinemet CR 25mg/100mg tablets | Carbidopa monohydrate/ Levodopa | |
| 8265041000033119 | Lecado 100mg/25mg modified-release tablets | Carbidopa monohydrate/ Levodopa | |
| 8265141000033115 | Lecado 200mg/50mg modified-release tablets | Carbidopa monohydrate/ Levodopa | |
| 12289941000033112 | Levodopa 100mg / Carbidopa 25mg / Entacapone 200mg tablets | Carbidopa/ Entacapone/ Levodopa | |
| 12290041000033119 | Levodopa 125mg / Carbidopa 31.25mg / Entacapone 200mg tablets | Carbidopa/ Entacapone/ Levodopa | |
| 12290141000033115 | Levodopa 150mg / Carbidopa 37.5mg / Entacapone 200mg tablets | Carbidopa/ Entacapone/ Levodopa | |
| 12290241000033110 | Levodopa 175mg / Carbidopa 43.75mg / Entacapone 200mg tablets | Carbidopa/ Entacapone/ Levodopa | |
| 12290341000033117 | Levodopa 200mg / Carbidopa 50mg / Entacapone 200mg tablets | Carbidopa/ Entacapone/ Levodopa | |
| 826341000033117 | Levodopa 500mg tablets | Levodopa | |
| 12290441000033111 | Levodopa 50mg / Carbidopa 12.5mg / Entacapone 200mg tablets | Carbidopa/ Entacapone/ Levodopa | |
| 12290541000033112 | Levodopa 75mg / Carbidopa 18.75mg / Entacapone 200mg tablets | Carbidopa/ Entacapone/ Levodopa | |
| 857541000033114 | Madopar 100mg/25mg capsules | Benserazide hydrochloride/ Levodopa | |
| 871341000033111 | Madopar 100mg/25mg dispersible tablets | Benserazide hydrochloride/ Levodopa | |
| 858341000033115 | Madopar 200mg/50mg capsules | Benserazide hydrochloride/ Levodopa | |
| 857641000033110 | Madopar 50mg/12.5mg capsules | Benserazide hydrochloride/ Levodopa | |
| 871241000033118 | Madopar 50mg/12.5mg dispersible tablets | Benserazide hydrochloride/ Levodopa | |
| 858441000033114 | Madopar CR capsules | Benserazide hydrochloride/ Levodopa | |
| 9836241000033115 | Sastravi 100mg/25mg/200mg tablets | Carbidopa/ Entacapone/ Levodopa | |
| 9837541000033115 | Sastravi 125mg/31.25mg/200mg tablets | Carbidopa/ Entacapone/ Levodopa | |
| 9837641000033119 | Sastravi 150mg/37.5mg/200mg tablets | Carbidopa/ Entacapone/ Levodopa | |
| 9837741000033111 | Sastravi 175mg/43.75mg/200mg tablets | Carbidopa/ Entacapone/ Levodopa | |
| 9837841000033118 | Sastravi 200mg/50mg/200mg tablets | Carbidopa/ Entacapone/ Levodopa | |
| 9837941000033114 | Sastravi 50mg/12.5mg/200mg tablets | Carbidopa/ Entacapone/ Levodopa | |
| 9838041000033112 | Sastravi 75mg/18.75mg/200mg tablets | Carbidopa/ Entacapone/ Levodopa | |
| 1337041000033118 | Sinemet 10mg/100mg tablets | Carbidopa monohydrate/ Levodopa | |
| 1919441000033117 | Sinemet 12.5mg/50mg tablets | Carbidopa monohydrate/ Levodopa | |
| 1337141000033119 | Sinemet 25mg/250mg tablets | Carbidopa monohydrate/ Levodopa | |
| 1338041000033119 | Sinemet CR 50mg/200mg tablets | Carbidopa monohydrate/ Levodopa | |
| 1337241000033114 | Sinemet Plus 25mg/100mg tablets | Carbidopa monohydrate/ Levodopa | |
| 3006341000033117 | Stalevo 100mg/25mg/200mg tablets | Carbidopa/ Entacapone/ Levodopa | |
| 5076341000033116 | Stalevo 125mg/31.25mg/200mg tablets | Carbidopa/ Entacapone/ Levodopa | |
| 3006441000033111 | Stalevo 150mg/37.5mg/200mg tablets | Carbidopa/ Entacapone/ Levodopa | |
| 6512941000033113 | Stalevo 175mg/43.75mg/200mg tablets | Carbidopa/ Entacapone/ Levodopa | |
| 4561741000033111 | Stalevo 200mg/50mg/200mg tablets | Carbidopa/ Entacapone/ Levodopa | |
| 3006241000033110 | Stalevo 50mg/12.5mg/200mg tablets | Carbidopa/ Entacapone/ Levodopa | |
| 5076641000033112 | Stalevo 75mg/18.75mg/200mg tablets | Carbidopa/ Entacapone/ Levodopa | |
| 10251441000033118 | Stanek 100mg/25mg/200mg tablets | Carbidopa/ Entacapone/ Levodopa | |
| 10251541000033117 | Stanek 125mg/31.25mg/200mg tablets | Carbidopa/ Entacapone/ Levodopa | |
| 10251641000033116 | Stanek 150mg/37.5mg/200mg tablets | Carbidopa/ Entacapone/ Levodopa | |
| 10251741000033113 | Stanek 175mg/43.75mg/200mg tablets | Carbidopa/ Entacapone/ Levodopa | |
| 10252041000033117 | Stanek 200mg/50mg/200mg tablets | Carbidopa/ Entacapone/ Levodopa | |
| 10251841000033115 | Stanek 50mg/12.5mg/200mg tablets | Carbidopa/ Entacapone/ Levodopa | |
| 10251941000033111 | Stanek 75mg/18.75mg/200mg tablets | Carbidopa/ Entacapone/ Levodopa | |
| 3226541000033110 | Tilolec 100mg/25mg modified-release tablets | Carbidopa monohydrate/ Levodopa | |
| 3156641000033112 | Tilolec 200mg/50mg modified-release tablets | Carbidopa monohydrate/ Levodopa | |
| 1337941000033117 |  | Carbidopa monohydrate/ Levodopa | |
| 1766741000033114 | Pramipexole 88microgram tablets | Pramipexole dihydrochloride monohydrate | |
| 1766841000033116 | Pramipexole 180microgram tablets | Pramipexole dihydrochloride monohydrate | |
| 1766941000033112 | Pramipexole 700microgram tablets | Pramipexole dihydrochloride monohydrate | |
| 4429741000033114 | Pramipexole 350microgram tablets | Pramipexole dihydrochloride monohydrate | |
| 5318541000033115 | Pramipexole 520microgram modified-release tablets | Pramipexole dihydrochloride monohydrate | |
| 5318641000033119 | Pramipexole 2.1mg modified-release tablets | Pramipexole dihydrochloride monohydrate | |
| 5318741000033111 | Pramipexole 3.15mg modified-release tablets | Pramipexole dihydrochloride monohydrate | |
| 5318841000033118 | Pramipexole 1.05mg modified-release tablets | Pramipexole dihydrochloride monohydrate | |
| 5318941000033114 | Pramipexole 260microgram modified-release tablets | Pramipexole dihydrochloride monohydrate | |
| 5999441000033115 | Pramipexole 2.62mg modified-release tablets | Pramipexole dihydrochloride monohydrate | |
| 5999841000033117 | Pramipexole 1.57mg modified-release tablets | Pramipexole dihydrochloride monohydrate | |
| 1767041000033113 | Mirapexin 0.088mg tablets | Pramipexole dihydrochloride monohydrate | |
| 1767141000033112 | Mirapexin 0.18mg tablets | Pramipexole dihydrochloride monohydrate | |
| 1767241000033117 | Mirapexin 0.7mg tablets | Pramipexole dihydrochloride monohydrate | |
| 4429841000033116 | Mirapexin 0.35mg tablets | Pramipexole dihydrochloride monohydrate | |
| 5319041000033117 | Mirapexin 0.52mg modified-release tablets | Pramipexole dihydrochloride monohydrate | |
| 5319141000033118 | Mirapexin 2.1mg modified-release tablets | Pramipexole dihydrochloride monohydrate | |
| 5319241000033113 | Mirapexin 3.15mg modified-release tablets | Pramipexole dihydrochloride monohydrate | |
| 5319341000033115 | Mirapexin 1.05mg modified-release tablets | Pramipexole dihydrochloride monohydrate | |
| 5319441000033114 | Mirapexin 0.26mg modified-release tablets | Pramipexole dihydrochloride monohydrate | |
| 5999641000033118 | Mirapexin 2.62mg modified-release tablets | Pramipexole dihydrochloride monohydrate | |
| 5999941000033113 | Mirapexin 1.57mg modified-release tablets | Pramipexole dihydrochloride monohydrate | |
| 6063641000033111 | Neliprax 0.088mg tablets | Pramipexole dihydrochloride monohydrate | |
| 6063741000033119 | Neliprax 0.18mg tablets | Pramipexole dihydrochloride monohydrate | |
| 6065341000033116 | Oprymea 0.088mg tablets | Pramipexole dihydrochloride monohydrate | |
| 6065441000033110 | Oprymea 0.18mg tablets | Pramipexole dihydrochloride monohydrate | |
| 6065541000033111 | Oprymea 0.35mg tablets | Pramipexole dihydrochloride monohydrate | |
| 6065641000033112 | Oprymea 0.7mg tablets | Pramipexole dihydrochloride monohydrate | |
| 9068541000033112 | Oprymea 0.26mg modified-release tablets | Pramipexole dihydrochloride monohydrate | |
| 9068641000033113 | Oprymea 0.52mg modified-release tablets | Pramipexole dihydrochloride monohydrate | |
| 9068741000033116 | Oprymea 1.05mg modified-release tablets | Pramipexole dihydrochloride monohydrate | |
| 9068841000033114 | Oprymea 1.57mg modified-release tablets | Pramipexole dihydrochloride monohydrate | |
| 9068941000033118 | Oprymea 2.1mg modified-release tablets | Pramipexole dihydrochloride monohydrate | |
| 10982641000033119 | Oprymea 2.62mg modified-release tablets | Pramipexole dihydrochloride monohydrate | |
| 10983641000033110 | Oprymea 3.15mg modified-release tablets | Pramipexole dihydrochloride monohydrate | |
| 11810841000033117 | Pipexus 0.26mg modified-release tablets | Pramipexole dihydrochloride monohydrate | |
| 11810941000033113 | Pipexus 0.52mg modified-release tablets | Pramipexole dihydrochloride monohydrate | |
| 11811041000033115 | Pipexus 1.05mg modified-release tablets | Pramipexole dihydrochloride monohydrate | |
| 11811141000033116 | Pipexus 1.57mg modified-release tablets | Pramipexole dihydrochloride monohydrate | |
| 11811241000033111 | Pipexus 2.1mg modified-release tablets | Pramipexole dihydrochloride monohydrate | |
| 11811341000033118 | Pipexus 2.62mg modified-release tablets | Pramipexole dihydrochloride monohydrate | |
| 11811441000033112 | Pipexus 3.15mg modified-release tablets | Pramipexole dihydrochloride monohydrate | |
| 1184341000033112 | Ropinirole 1mg tablets | Ropinirole hydrochloride | |
| 1184441000033118 | Ropinirole 2mg tablets | Ropinirole hydrochloride | |
| 1184541000033117 | Ropinirole 250microgram tablets | Ropinirole hydrochloride | |
| 1184641000033116 | Ropinirole 5mg tablets | Ropinirole hydrochloride | |
| 2859841000033119 |  | Ropinirole hydrochloride | |
| 2864641000033113 |  | Ropinirole hydrochloride | |
| 3922741000033119 | Ropinirole 500microgram tablets | Ropinirole hydrochloride | |
| 4501641000033115 | Ropinirole 2mg modified-release tablets | Ropinirole hydrochloride | |
| 4501741000033112 | Ropinirole 4mg modified-release tablets | Ropinirole hydrochloride | |
| 4501841000033119 | Ropinirole 8mg modified-release tablets | Ropinirole hydrochloride | |
| 10266041000033112 | Ropinirole 3mg modified-release tablets | Ropinirole hydrochloride | |
| 10266141000033111 | Ropinirole 6mg modified-release tablets | Ropinirole hydrochloride | |
| 1165241000033118 | ReQuip 1mg tablets | Ropinirole hydrochloride | |
| 1165341000033111 | ReQuip 2mg tablets | Ropinirole hydrochloride | |
| 1165441000033117 | ReQuip 250microgram tablets | Ropinirole hydrochloride | |
| 1165541000033116 | ReQuip 5mg tablets | Ropinirole hydrochloride | |
| 3922841000033112 | Adartrel 250microgram tablets | Ropinirole hydrochloride | |
| 3922941000033116 | Adartrel 500microgram tablets | Ropinirole hydrochloride | |
| 3923041000033114 | Adartrel 2mg tablets | Ropinirole hydrochloride | |
| 4501941000033110 | ReQuip XL 2mg tablets | Ropinirole hydrochloride | |
| 4502041000033116 | ReQuip XL 4mg tablets | Ropinirole hydrochloride | |
| 4502141000033117 | ReQuip XL 8mg tablets | Ropinirole hydrochloride | |
| 6385841000033110 | Spiroco XL 2mg tablets | Ropinirole hydrochloride | |
| 6385941000033119 | Spiroco XL 4mg tablets | Ropinirole hydrochloride | |
| 6386041000033112 | Spiroco XL 8mg tablets | Ropinirole hydrochloride | |
| 6391441000033114 | Ralnea XL 2mg tablets | Ropinirole hydrochloride | |
| 6391541000033110 | Ralnea XL 4mg tablets | Ropinirole hydrochloride | |
| 6391641000033111 | Ralnea XL 8mg tablets | Ropinirole hydrochloride | |
| 8048641000033117 | Repinex XL 2mg tablets | Ropinirole hydrochloride | |
| 8048741000033114 | Repinex XL 4mg tablets | Ropinirole hydrochloride | |
| 8048841000033116 | Repinex XL 8mg tablets | Ropinirole hydrochloride | |
| 8243341000033113 | Raponer XL 2mg tablets | Ropinirole hydrochloride | |
| 8243441000033119 | Raponer XL 4mg tablets | Ropinirole hydrochloride | |
| 8243541000033118 | Raponer XL 8mg tablets | Ropinirole hydrochloride | |
| 8274141000033112 | Aimpart XL 2mg tablets | Ropinirole hydrochloride | |
| 8274241000033117 | Aimpart XL 4mg tablets | Ropinirole hydrochloride | |
| 8274341000033110 | Aimpart XL 8mg tablets | Ropinirole hydrochloride | |
| 11798341000033110 | Ipinnia XL 2mg tablets | Ropinirole hydrochloride | |
| 11798441000033116 | Ipinnia XL 3mg tablets | Ropinirole hydrochloride | |
| 11798541000033115 | Ipinnia XL 4mg tablets | Ropinirole hydrochloride | |
| 11798641000033119 | Ipinnia XL 6mg tablets | Ropinirole hydrochloride | |
| 11798741000033111 | Ipinnia XL 8mg tablets | Ropinirole hydrochloride | |
| 12186141000033115 | Ropilynz XL 2mg tablets | Ropinirole hydrochloride | |
| 12186241000033110 | Ropilynz XL 4mg tablets | Ropinirole hydrochloride | |
| 12186341000033117 | Ropilynz XL 8mg tablets | Ropinirole hydrochloride | |
| 12604841000033118 | Ropiqual XL 2mg tablets | Ropinirole hydrochloride | |
| 12604941000033114 | Ropiqual XL 4mg tablets | Ropinirole hydrochloride | |
| 12605041000033114 | Ropiqual XL 8mg tablets | Ropinirole hydrochloride | |
| 2859941000033110 | ReQuip tablets starter pack | Ropinirole hydrochloride | |
| 2864741000033116 | ReQuip tablets follow on pack | Ropinirole hydrochloride | |
| 3906041000033110 | Rotigotine 2mg/24hours transdermal patches | Rotigotine | |
| 3906141000033114 | Rotigotine 4mg/24hours transdermal patches | Rotigotine | |
| 3906241000033119 | Rotigotine 6mg/24hours transdermal patches | Rotigotine | |
| 3906341000033112 | Rotigotine 8mg/24hours transdermal patches | Rotigotine | |
| 5129741000033118 | Rotigotine 1mg/24hours transdermal patches | Rotigotine | |
| 5129841000033111 | Rotigotine 3mg/24hours transdermal patches | Rotigotine | |
| 3905941000033117 |  | Rotigotine | |
| 3906541000033117 | Neupro 2mg/24hours transdermal patches | Rotigotine | |
| 3906641000033116 | Neupro 4mg/24hours transdermal patches | Rotigotine | |
| 3906741000033113 | Neupro 6mg/24hours transdermal patches | Rotigotine | |
| 3906841000033115 | Neupro 8mg/24hours transdermal patches | Rotigotine | |
| 5130041000033111 | Neupro 1mg/24hours transdermal patches | Rotigotine | |
| 5130141000033110 | Neupro 3mg/24hours transdermal patches | Rotigotine | |
| 3906441000033118 | Neupro transdermal patches treatment initiation pack | Rotigotine | |
| 507441000033119 | Eldepryl 10mg/5ml syrup | Selegiline hydrochloride | |
| 507941000033112 | Eldepryl 10mg tablets | Selegiline hydrochloride | |
| 508741000033113 | Eldepryl 5mg tablets | Selegiline hydrochloride | |
| 1274341000033115 | Selegiline 10mg/5ml oral solution | Selegiline hydrochloride | |
| 1274541000033110 | Selegiline 10mg tablets | Selegiline hydrochloride | |
| 1278141000033113 | Selegiline 5mg tablets | Selegiline hydrochloride | |
| 1752441000033113 | Selegiline 1.25mg oral lyophilisates sugar free | Selegiline hydrochloride | |
| 1765641000033113 | Zelapar 1.25mg oral lyophilisates | Selegiline hydrochloride | |
| 3307741000033115 | Rasagiline 1mg tablets | Rasagiline | |
| 3307841000033113 | Azilect 1mg tablets | Rasagiline | |
| 14260341000033114 | Rasagiline 1mg tablets | Rasagiline | |
| 73141000033113 | Apomorphine 20mg/2ml solution for injection ampoules | Apomorphine hydrochloride | |
| 73241000033118 | Apomorphine 50mg/5ml solution for injection ampoules | Apomorphine hydrochloride | |
| 73841000033119 | Apomorphine 30mg/3ml solution for injection pre-filled disposable devices | Apomorphine hydrochloride | |
| 2214141000033114 | APO-go 20mg/2ml solution for injection ampoules | Apomorphine hydrochloride | |
| 2214241000033119 | APO-go 50mg/5ml solution for injection ampoules | Apomorphine hydrochloride | |
| 2584741000033115 | Apomorphine 2mg sublingual tablets sugar free | Apomorphine hydrochloride | |
| 2584841000033113 | Apomorphine 3mg sublingual tablets sugar free | Apomorphine hydrochloride | |
| 2584941000033117 | Uprima 2mg sublingual tablets | Apomorphine hydrochloride | |
| 2585041000033117 | Uprima 3mg sublingual tablets | Apomorphine hydrochloride | |
| 2740141000033117 | APO-go PEN 30mg/3ml solution for injection | Apomorphine hydrochloride | |
| 3227841000033110 | Apomorphine 50mg/10ml solution for infusion pre-filled syringes | Apomorphine hydrochloride | |
| 3310441000033112 | APO-go PFS 50mg/10ml solution for infusion pre-filled syringes | Apomorphine hydrochloride | |
| 12875741000033110 | Apomorphine 30mg/3ml solution for injection cartridges | Apomorphine hydrochloride hemihydrate | |
| 12875841000033117 | Apomorphine 100mg/20ml solution for infusion vials | Apomorphine hydrochloride hemihydrate | |
| 12875941000033113 | Dacepton 100mg/20ml solution for infusion vials | Apomorphine hydrochloride hemihydrate | |
| 12876041000033115 | Dacepton 30mg/3ml solution for injection cartridges | Apomorphine hydrochloride hemihydrate | |
| 14146441000033110 | Apomorphine 100mg/20ml solution for infusion cartridges | Apomorphine hydrochloride hemihydrate | |
| 14146541000033111 | APO-go POD 100mg/20ml solution for infusion cartridges | Apomorphine hydrochloride hemihydrate | |
| 3227941000033119 |  | Apomorphine hydrochloride hemihydrate | |
| 11756941000033118 | Opicapone 50mg capsules | Opicapone | |
| 11757141000033118 | Ongentys 50mg capsules | Opicapone | |
| 11580141000033114 | Safinamide 100mg tablets | Safinamide methansulfonate | |
| 11580241000033119 | Safinamide 50mg tablets | Safinamide methansulfonate | |
| 11580341000033112 | Xadago 100mg tablets | Safinamide methansulfonate | |
| 11580441000033118 | Xadago 50mg tablets | Safinamide methansulfonate | |
| 156541000033115 |  | Brocadopa | |
| 156641000033119 |  | Brocadopa | |
| 156741000033111 |  | Brocadopa | |

## Table S11. Product codes for motor neurone disease medications

| **ProdCodeId** | **Product name** | **Drug substance name** |
| --- | --- | --- |
| 1177441000033116 | Rilutek 50mg tablets | Riluzole |
| 1177541000033115 | Riluzole 50mg tablets | Riluzole |
| 10714441000033118 | Riluzole 25mg/5ml oral suspension sugar free | Riluzole |
| 10714541000033117 | Teglutik 5mg/1ml oral suspension | Riluzole |
| 14227441000033114 | Riluzole 50mg orodispersible films | Riluzole |
| 14227541000033110 | Emylif 50mg orodispersible films | Riluzole |

## Table S12. Medical codes used to determine living situation (lives alone, does not live alone, living in a care facility or homeless)

| **MedCodeId** | **Term** | **Living situation** |
| --- | --- | --- |
| 1227760014 | [V]Delayed discharge - nursing home vacancy awaited | Lives in care facility |
| 1227796013 | [V]Old age home admission medical | Lives in care facility |
| 461151010 | [V]Other boarder in health-care facility | Lives in care facility |
| 360071000006115 | [X]Accidental poisoning by and exposure to organic solvents and halogenated hydrocarbons and their vapours, occurrence in residential institution | Lives in care facility |
| 361011000006116 | [X]Accidental poisoning by and exposure to other autonomic drugs, occurrence in residential institution | Lives in care facility |
| 361621000006118 | [X]Accidental suffocation and strangulation in bed, occurrence in residential institution | Lives in care facility |
| 364051000006118 | [X]Assault by blunt object occurrn in resident institution | Lives in care facility |
| 364141000006116 | [X]Assault by bodily force occurrn in residential institut'n | Lives in care facility |
| 364221000006118 | [X]Assault by corrosiv substnc occurrn in resident instit'n | Lives in care facility |
| 364361000006119 | [X]Assault by crash of motor vehicle occ resident instit'n | Lives in care facility |
| 364501000006113 | [X]Assault by drowning+submersion occurrn resident instit'n | Lives in care facility |
| 364581000006116 | [X]Assault by drug medicamnt+biolog substn occ resid instit | Lives in care facility |
| 364651000006113 | [X]Assault by explosiv material occurrn resident institut'n | Lives in care facility |
| 364791000006114 | [X]Assault by gases + vapours occurrn in resident instit'n | Lives in care facility |
| 364921000006114 | [X]Assault by handgun discharge occurrn resident institutn | Lives in care facility |
| 364991000006111 | [X]Assault by hanging strangul+suffoc occ resident instit'n | Lives in care facility |
| 365071000006110 | [X]Assault by oth specif chem+noxous substn resid instit'n | Lives in care facility |
|  |  |  |
| 365141000006115 | [X]Assault by oth specif means occurrn resident institution | Lives in care facility |
| 365291000006115 | [X]Assault by pesticides, occurrence in residential institution | Lives in care facility |
| 365401000006111 | [X]Assault by push from high place occurrn resident instit'n | Lives in care facility |
| 366361000006114 | [X]Assault by pushing or placing victim before moving object, occurrence in residential institution | Lives in care facility |
| 365601000006113 | [X]Assault by sharp object occurrn in resident institution | Lives in care facility |
| 365701000006118 | [X]Assault by smoke fire+flame occurrn resident institution | Lives in care facility |
| 365801000006114 | [X]Assault by steam, hot vapours and hot objects, occurrence in residential institution | Lives in care facility |
| 365951000006116 | [X]Assault by unspecified means occurrn resident institut'n | Lives in care facility |
| 366101000006110 | [X]Assault oth+unsp firearm discharge occ in resid instit'n | Lives in care facility |
| 366171000006116 | [X]Assault rifl s'gun+larg firearm disch occ resid instit'n | Lives in care facility |
| 366281000006114 | [X]Assault unspecif chemical/noxous subst occ resid instit'n | Lives in care facility |
| 333424016 | [X]Bitten by rat, occurrence in residential institution | Lives in care facility |
| 367641000006119 | [X]Bitten/crushed by oth reptiles occurrn resident instit'n | Lives in care facility |
| 367681000006113 | [X]Bitten/struck by crocodil/alligatr occ in resid instit'n | Lives in care facility |
| 367771000006117 | [X]Bitten/struck by dog occurrnce in residential institut'n | Lives in care facility |
| 367801000006115 | [X]Bitten/struck by oth mammal occurrn in resident instit'n | Lives in care facility |
| 371481000006113 | [X]Confind to/trappd in low-oxygen environ occ resid instit | Lives in care facility |
| 371971000006118 | [X]Contact hot heatng applianc radiatr+pipe occ resid instit | Lives in care facility |
| 372041000006118 | [X]Contact oth+unspecif heat+hot substnc occ resid instit'n | Lives in care facility |
| 372081000006112 | [X]Contact with agricultural machinery, occurrence in residential institution | Lives in care facility |
| 375081000006117 | [X]Contact with blunt object, undetermined intent, occurrence in residential institution | Lives in care facility |
| 375111000006111 | [X]Contact with centipedes and venomous millipedes (tropical), occurrence in residential institution | Lives in care facility |
| 374891000006110 | [X]Contact with explosive material, undetermined intent, occurrence in residential institution | Lives in care facility |
| 374931000006118 | [X]Contact with hornets, wasps and bees, occurrence in residential institution | Lives in care facility |
| 372371000006112 | [X]Contact with hot air and gases occurrn resident instit'n | Lives in care facility |
| 372481000006118 | [X]Contact with hot engin machinry+tool occ resident instit | Lives in care facility |
| 372631000006116 | [X]Contact with hot tap-water occurrn in resident instit'n | Lives in care facility |
| 372801000006113 | [X]Contact with lifting and transmission devices NEC, occurrence in residential institution | Lives in care facility |
| 373301000006112 | [X]Contact with other hot fluids occurrn resident instit'n | Lives in care facility |
| 373091000006115 | [X]Contact with other hot metals, occurrence in residential institution | Lives in care facility |
| 371901000006112 | [X]Contact with other powered hand tools and household machinery, occurrence in residential institution | Lives in care facility |
| 373451000006116 | [X]Contact with other+unspecif machinry occ in resid instit | Lives in care facility |
| 375001000006114 | [X]Contact with powered lawnmower, occurrence in residential institution | Lives in care facility |
| 373671000006112 | [X]Contact with scorpions, occurrence in residential institution | Lives in care facility |
| 373781000006116 | [X]Contact with sharp glass occurrn in residential instit'n | Lives in care facility |
| 375511000006115 | [X]Contact with sharp object, undetermined intent, occurrence in residential institution | Lives in care facility |
| 374001000006113 | [X]Contact with steam and hot vapours, occurrence in residential institution | Lives in care facility |
| 373951000006110 | [X]Contact with steam, hot vapours and hot objects, undetermined intent, occurrence in residential institution | Lives in care facility |
| 375621000006110 | [X]Contact with venomous marine animals and plants, occurrence in residential institution | Lives in care facility |
| 374481000006110 | [X]Contact with venomous snakes and lizards, occurrence in residential institution | Lives in care facility |
| 375031000006118 | [X]Contact with venomous spiders, occurrence in residential institution | Lives in care facility |
| 374341000006112 | [X]Contact wth knife sword/dagger occurrn in resid instit'n | Lives in care facility |
| 374401000006118 | [X]Contact wth marine animal occurrn in resident institut'n | Lives in care facility |
| 374411000006115 | [X]Contact wth nonpowerd hand tool occ in resident instit'n | Lives in care facility |
| 374981000006117 | [X]Contct with oth specif venomous plant occ resid instit'n | Lives in care facility |
| 375201000006115 | [X]Contct wth hot drink food fat+cookng oil occ resid instit | Lives in care facility |
| 375251000006116 | [X]Contct wth hot househld applianc occurrn resident instit | Lives in care facility |
| 375401000006119 | [X]Contct wth oth specif venom arthropd occ resid instit'n | Lives in care facility |
| 375431000006110 | [X]Contct wth oth specif venomous animal occ resid instit'n | Lives in care facility |
| 375451000006115 | [X]Contct wth plant thorn+spine+sharp leave occ resid instit | Lives in care facility |
| 375541000006116 | [X]Contct wth unspec venomous animal/plant occ resid instit | Lives in care facility |
| 375921000006117 | [X]Crashing of motor vehicle, undetermined intent, occurrence in residential institution | Lives in care facility |
| 376071000006113 | [X]Crushed, pushed or stepped on by crowd or human stampede, occurrence in residential institution | Lives in care facility |
| 377161000006117 | [X]Disch from other/unspecif firearms occ in resid instit'n | Lives in care facility |
| 377241000006113 | [X]Discharge of firework, occurrence in residential institution | Lives in care facility |
| 378181000006111 | [X]Diving or jumping into water causing injury other than drowning or submersion, occurrence in residential institution | Lives in care facility |
| 378821000006118 | [X]Drowning and submersion following fall into bath-tub, occurrence in residential institution | Lives in care facility |
| 378801000006111 | [X]Drowning and submersion following fall into natural water, occurrence in residential institution | Lives in care facility |
| 378721000006113 | [X]Drowning and submersion following fall into swimming-pool, occurrence in residential institution | Lives in care facility |
| 378871000006117 | [X]Drowning and submersion while in bath-tub, occurrence in residential institution | Lives in care facility |
| 378761000006119 | [X]Drowning and submersion while in natural water, occurrence in residential institution | Lives in care facility |
| 378791000006110 | [X]Drowning and submersion while in swimming-pool, occurrence in residential institution | Lives in care facility |
| 379071000006118 | [X]Drowning and submersion, undetermined intent, occurrence in residential institution | Lives in care facility |
| 380321000006116 | [X]Explosion and rupture of boiler, occurrence in residential institution | Lives in care facility |
| 380771000006116 | [X]Explosion and rupture of gas cylinder, occurrence in residential institution | Lives in care facility |
| 380711000006113 | [X]Explosion and rupture of other specified pressurized devices, occurrence in residential institution | Lives in care facility |
| 380641000006118 | [X]Explosion and rupture of pressurised tyre, pipe or hose, occurrence in residential institution | Lives in care facility |
| 380441000006115 | [X]Explosion of other material occurrn in resident instit'n | Lives in care facility |
| 381141000006116 | [X]Expos to man-mde visibl+ultraviol light occ resid instit | Lives in care facility |
| 381221000006118 | [X]Expos to oth+unsp inanim mechan force occ resid instit'n | Lives in care facility |
| 381391000006110 | [X]Expos unspecif type of radiatn occurrn resident instit'n | Lives in care facility |
| 381521000006115 | [X]Exposr excess heat man-made origin occurrn resid instit'n | Lives in care facility |
| 381691000006119 | [X]Exposr oth nonionizing radiatn occurrn resident instit'n | Lives in care facility |
| 381811000006113 | [X]Exposr to electrc transmiss line occurrn in resid instit | Lives in care facility |
| 381891000006115 | [X]Exposr to ionizng radiation occurrn in resident instit'n | Lives in care facility |
| 381931000006112 | [X]Exposr to oth specif electrc currnt occurrn resid instit | Lives in care facility |
| 381961000006115 | [X]Exposr to oth specif smoke fire+flame occ resid instit'n | Lives in care facility |
| 382571000006111 | [X]Exposur to excessv naturl heat occurrn resident institut | Lives in care facility |
| 382711000006114 | [X]Exposur to oth+unsp animat mechan force occ resid instit | Lives in care facility |
| 382871000006114 | [X]Exposur unspecif smoke fire/flame occurrn resid instit'n | Lives in care facility |
| 382171000006118 | [X]Exposure to controlled fire in building or structure, occurrence in residential institution | Lives in care facility |
| 381471000006118 | [X]Exposure to controlled fire, not in building or structure, occurrence in residential institution | Lives in care facility |
| 381491000006117 | [X]Exposure to excessive cold of man-made origin, occurrence in residential institution | Lives in care facility |
| 382561000006116 | [X]Exposure to excessive natural cold, occurrence in residential institution | Lives in care facility |
| 380921000006115 | [X]Exposure to high and low air pressure and changes in air pressure, occurrence in residential institution | Lives in care facility |
| 383311000006118 | [X]Exposure to high-pressure jet, occurrence in residential institution | Lives in care facility |
| 382261000006118 | [X]Exposure to ignition of highly flammable material, occurrence in residential institution | Lives in care facility |
| 382661000006115 | [X]Exposure to ignition or melting of nightwear, occurrence in residential institution | Lives in care facility |
| 381641000006111 | [X]Exposure to ignition or melting of other clothing and apparel, occurrence in residential institution | Lives in care facility |
| 333338010 | [X]Exposure to noise, occurrence in residential institution | Lives in care facility |
| 383001000006118 | [X]Exposure to other and unspecified forces of nature, occurrence in residential institution | Lives in care facility |
| 381781000006111 | [X]Exposure to other and unspecified man-made environmental factors, occurrence in residential institution | Lives in care facility |
| 382701000006111 | [X]Exposure to other specified factors, occurrence in residential institution | Lives in care facility |
| 382031000006117 | [X]Exposure to smoke, fire and flames, undetermined intent, occurrence in residential institution | Lives in care facility |
| 383931000006113 | [X]Exposure to sunlight occurrn in residential institution | Lives in care facility |
| 382801000006115 | [X]Exposure to uncontrolled fire in building or structure, occurrence in residential institution | Lives in care facility |
| 382111000006110 | [X]Exposure to uncontrolled fire, not in building or structure, occurrence in residential institution | Lives in care facility |
| 384061000006119 | [X]Exposure to unspecif electric current occ resid instit'n | Lives in care facility |
| 384111000006118 | [X]Exposure to unspecified factor, occurrence in residential institution | Lives in care facility |
| 384281000006117 | [X]Exposure to vibration occurrn in residential institution | Lives in care facility |
| 332995014 | [X]Fall from cliff, occurrence in residential institution | Lives in care facility |
| 384791000006112 | [X]Fall from out of/thro buildng/struct occ resid instit'n | Lives in care facility |
| 332983014 | [X]Fall from tree, occurrence in residential institution | Lives in care facility |
| 385221000006115 | [X]Fall involv other furniture occurrn resident institut'n | Lives in care facility |
| 385251000006112 | [X]Fall involv playgrnd equipm occurr in resident instit'n | Lives in care facility |
| 385351000006118 | [X]Fall involving bed occurrence in residential institution | Lives in care facility |
| 385461000006110 | [X]Fall involving chair occurrence in residential instit'n | Lives in care facility |
| 385631000006115 | [X]Fall involvng wheelchair occurrence residential instit'n | Lives in care facility |
| 385831000006116 | [X]Fall on + from ladder occurrn in residential institution | Lives in care facility |
| 385951000006119 | [X]Fall on + from stair + step occurrnce resident instit'n | Lives in care facility |
| 386151000006112 | [X]Fall on+from scaffold occurrn in residential institution | Lives in care facility |
| 386201000006115 | [X]Fall same level from slip trip + stumb occ resid instit | Lives in care facility |
| 386231000006111 | [X]Fall same level involv ice / snow occurrn resid instit'n | Lives in care facility |
| 386461000006118 | [X]Fall whle carried/supported oth persons occ resid instit | Lives in care facility |
| 385651000006110 | [X]Falling, jumping or pushed from a high place, undetermined intent, occurrence in residential institution | Lives in care facility |
| 385761000006115 | [X]Falling, lying or running before or into moving object, undetermined intent, occurrence in residential institution | Lives in care facility |
| 387231000006113 | [X]Foreign body enter into/thr eye/natrl orif, resid instit | Lives in care facility |
| 387411000006118 | [X]Foreign body or object entering through skin, occurrence in residential institution | Lives in care facility |
| 333194018 | [X]Handgun discharge, occurrence in residential institution | Lives in care facility |
| 388451000006116 | [X]Handgun discharge, undetermined intent, occurrence in residential institution | Lives in care facility |
| 388671000006112 | [X]Hanging, strangulation and suffocation, undetermined intent, occurrence in residential institution | Lives in care facility |
| 388951000006110 | [X]Hit, struck, kicked, twisted, bitten or scratched by another person, occurrence in residential institution | Lives in care facility |
| 1817261000006116 | [X]Hypodermic needle injury, occur residential institution | Lives in care facility |
| 389911000006117 | [X]Inhalation and ingestion of food causing obstruction of respiratory tract, occurrence in residential institution | Lives in care facility |
| 390071000006117 | [X]Inhalation of gastric contents occurrn resident instit'n | Lives in care facility |
| 390221000006110 | [X]Inhl+ingest oth obj caus obst resp tract occ resid instit | Lives in care facility |
| 392701000006110 | [X]Intent self harm by hangng strangult/suffoct resid instit | Lives in care facility |
| 392301000006114 | [X]Intentional self harm by blunt object, occurrence in residential institution | Lives in care facility |
| 392351000006113 | [X]Intentional self harm by crashing of motor vehicle, occurrence in residential institution | Lives in care facility |
| 392471000006111 | [X]Intentional self harm by drowning and submersion, occurrence in residential institution | Lives in care facility |
| 394001000006119 | [X]Intentional self harm by explosive material, occurrence in residential institution | Lives in care facility |
| 392571000006110 | [X]Intentional self harm by handgun discharge, occurrence in residential institution | Lives in care facility |
| 12718261000006117 | [X]Intentional self harm by hanging, strangulation and suffocation, occurrence in residential institution | Lives in care facility |
| 392721000006117 | [X]Intentional self harm by jumping from a high place, occurrence in residential institution | Lives in care facility |
| 391271000006116 | [X]Intentional self harm by jumping or lying before moving object, occurrence in residential institution | Lives in care facility |
| 393191000006113 | [X]Intentional self harm by other and unspecified firearm discharge, occurrence in residential institution | Lives in care facility |
| 392811000006116 | [X]Intentional self harm by other specified means, occurrence in residential institution | Lives in care facility |
| 391291000006115 | [X]Intentional self harm by rifle, shotgun and larger firearm discharge, occurrence in residential institution | Lives in care facility |
| 392891000006114 | [X]Intentional self harm by sharp object, occurrence in residential institution | Lives in care facility |
| 11917431000006116 | [X]Intentional self harm by sharp object, occurrence in residential institution | Lives in care facility |
| 392961000006114 | [X]Intentional self harm by smoke, fire and flames, occurrence in residential institution | Lives in care facility |
| 393021000006110 | [X]Intentional self harm by steam, hot vapours and hot objects, occurrence in residential institution | Lives in care facility |
| 393091000006112 | [X]Intentional self harm by unspecified means, occurrence in residential institution | Lives in care facility |
| 393591000006118 | [X]Intentional self poisoning by and exposure to alcohol, occurrence in residential institution | Lives in care facility |
| 393611000006112 | [X]Intentional self poisoning by and exposure to antiepileptics, occurrence in residential institution | Lives in care facility |
| 393641000006111 | [X]Intentional self poisoning by and exposure to antiparkinson drugs, occurrence in residential institution | Lives in care facility |
| 393661000006110 | [X]Intentional self poisoning by and exposure to hallucinogens, occurrence in residential institution | Lives in care facility |
| 393681000006117 | [X]Intentional self poisoning by and exposure to narcotic drugs, occurrence in residential institution | Lives in care facility |
| 393701000006119 | [X]Intentional self poisoning by and exposure to nonopioid analgesics, occurrence in residential institution | Lives in care facility |
| 391701000006118 | [X]Intentional self poisoning by and exposure to organic solvents and halogenated hydrocarbons and their vapours, occurrence in residential institution | Lives in care facility |
| 393721000006112 | [X]Intentional self poisoning by and exposure to other autonomic drugs, occurrence in residential institution | Lives in care facility |
| 393781000006111 | [X]Intentional self poisoning by and exposure to pesticides, occurrence in residential institution | Lives in care facility |
| 393801000006110 | [X]Intentional self poisoning by and exposure to psychotropic drugs, occurrence in residential institution | Lives in care facility |
| 393821000006117 | [X]Intentional self poisoning by and exposure to sedative hypnotics, occurrence in residential institution | Lives in care facility |
| 334550011 | [X]Lack of food, occurrence in residential institution | Lives in care facility |
| 334562010 | [X]Lack of water, occurrence in residential institution | Lives in care facility |
| 404331000006115 | [X]Other accidental hanging and strangulation, occurrence in residential institution | Lives in care facility |
| 405661000006117 | [X]Other and unspecified firearm discharge, undetermined intent, occurrence in residential institution | Lives in care facility |
| 461467011 | [X]Other boarder in health care facility | Lives in care facility |
| 410141000006119 | [X]Other fall on same level, occurrnce in resident instit'n | Lives in care facility |
| 405251000006117 | [X]Other specified events, undetermined intent, occurrence in residential institution | Lives in care facility |
| 405371000006118 | [X]Other specified threats to breathing, occurrence in residential institution | Lives in care facility |
| 417961000006119 | [X]Overexertn+strenuous/repetitv movement occ resid instit'n | Lives in care facility |
| 421791000006112 | [X]Poisoning by and exposure to alcohol, occurrence in residential institution, undetermined intent | Lives in care facility |
| 421811000006111 | [X]Poisoning by and exposure to antiepileptics, occurrence in residential institution, undetermined intent | Lives in care facility |
| 421841000006110 | [X]Poisoning by and exposure to antiparkinson drugs, occurrence in residential institution, undetermined intent | Lives in care facility |
| 421861000006114 | [X]Poisoning by and exposure to hallucinogens, occurrence in residential institution, undetermined intent | Lives in care facility |
| 421881000006116 | [X]Poisoning by and exposure to narcotic drugs, occurrence in residential institution, undetermined intent | Lives in care facility |
| 421181000006113 | [X]Poisoning by and exposure to organic solvents and halogenated hydrocarbons and their vapours, occurrence in residential institution, undetermined intent | Lives in care facility |
| 421921000006112 | [X]Poisoning by and exposure to other autonomic drugs, occurrence in residential institution, undetermined intent | Lives in care facility |
| 421961000006118 | [X]Poisoning by and exposure to other gas and vapours, occurrence in residential institution, undetermined intent | Lives in care facility |
| 422001000006111 | [X]Poisoning by and exposure to psychotropic drugs, occurrence in residential institution, undetermined intent | Lives in care facility |
| 422021000006118 | [X]Poisoning by and exposure to sedative hypnotics, occurrence in residential institution, undetermined intent | Lives in care facility |
| 423591000006117 | [X]Prolonged stay in weightless environment, occurrence in residential institution | Lives in care facility |
| 425201000006111 | [X]Rifle shotgun + larger firearm disch occ in resid instit | Lives in care facility |
| 425241000006113 | [X]Rifle, shotgun and larger firearm discharge, undetermined intent, occurrence in residential institution | Lives in care facility |
| 426721000006117 | [X]Sexual assault by bodily force occurrn resident instit'n | Lives in care facility |
| 427851000006117 | [X]Striking against or bumped into by another person, occurrence in residential institution | Lives in care facility |
| 427991000006115 | [X]Striking against or struck by other objects, occurrence in residential institution | Lives in care facility |
| 334527017 | [X]Travel and motion, occurrence in residential institution | Lives in care facility |
| 430451000006118 | [X]Unspecif drowning+submersion occurrn in resid instit'n | Lives in care facility |
| 430541000006119 | [X]Unspecified event, undetermined intent, occurrence in residential institution | Lives in care facility |
| 333042017 | [X]Unspecified fall, occurrence in residential institution | Lives in care facility |
| 430681000006112 | [X]Unspecified privation, occurrence in residential institution | Lives in care facility |
| 430771000006116 | [X]Unspecified threat to breathing, occurrence in residential institution | Lives in care facility |
| 432361000006111 | [X]Victim of avalanche, landslide and other earth movements, occurrence in residential institution | Lives in care facility |
| 431851000006114 | [X]Victim of cataclysmic storm, occurrence in residential institution | Lives in care facility |
| 431921000006114 | [X]Victim of earthquake occurrn in residential institution | Lives in care facility |
| 334315019 | [X]Victim of flood, occurrence in residential institution | Lives in care facility |
| 432191000006117 | [X]Victim of lightning, occurrence in residential institution | Lives in care facility |
| 432271000006113 | [X]Victim of volcanic eruption, occurrence in residential institution | Lives in care facility |
| 442501000006118 | Accident caused by electric wiring and appliances in residential institution | Lives in care facility |
| 445801000006113 | Accident/poisoning occurred in residential institution NOS | Lives in care facility |
| 1872371000006114 | Accom status - acute/long stay residential facility/hospital | Lives in care facility |
| 1872341000006118 | Accom status - mental health registered care home | Lives in care facility |
| 1872471000006118 | Accom status - non-mental health registered care home | Lives in care facility |
| 1872581000006116 | Accom status - nursing home for older persons | Lives in care facility |
| 1872581000006116 | Accom status - nursing home for older persons | Lives in care facility |
| 1872431000006116 | Accom status - other acute/long stay resident. facility/hospital | Lives in care facility |
| 1872281000006119 | Accom status - staying with friends/family as a short term guest | Lives with others |
| 1872331000006111 | Accom status - supported group home by staff/resident caretaker | Lives in care facility |
| 1777711000006115 | Activity location: Hospice | Lives in care facility |
| 14282491000006110 | Activity location: Integrated Care Home Without Nursing and Care Home With Nursing | Lives in care facility |
| 1777731000006114 | Activity location: Nursing Home | Lives in care facility |
| 1777861000006110 | Activity location: Prison | Lives with others |
| 1777721000006111 | Activity location: Residential Care Home | Lives in care facility |
| 447725019 | Admission to hospice | Lives in care facility |
| 1936211000006119 | Admission to hospice from acute hospital | Lives in care facility |
| 1936211000006119 | Admission to hospice from acute hospital | Lives in care facility |
| 1936201000006117 | Admission to hospice from care home | Lives in care facility |
| 1936261000006116 | Admission to hospice from community hospital | Lives in care facility |
| 1936371000006119 | Admission to hospice from other location | Lives in care facility |
| 1936191000006115 | Admission to hospice from patient's home | Lives in care facility |
| 1957891000006114 | Admission to hospice out of hours | Lives in care facility |
| 5939131000006114 | Admission to long stay hospital | Lives in care facility |
| 1692851000000118 | Admission to nursing home | Lives in care facility |
| 8121831000006114 | Application by prisoner to see clinic nurse | Lives with others |
| 8121851000006119 | Application by prisoner to see dentist | Lives with others |
| 8121821000006111 | Application by prisoner to see doctor | Lives with others |
| 8121811000006115 | Application by prisoner to see health professional | Lives with others |
| 2328721000000118 | Assessment of risk of prisoner in-possession medication | Lives with others |
| 534551000006118 | Carbon monoxide fumes from fire in other residential home | Lives in care facility |
| 1920601000006115 | Care home visit for annual patient review | Lives in care facility |
| 1920611000006117 | Care home visit for mid-year patient review | Lives in care facility |
| 8088861000006113 | Cares for dependent relative at home | Lives with others |
| 1991821000006111 | Children living in the home | Lives with others |
| 1991831000006114 | Children living in the home - permanently | Lives with others |
| 1488623014 | Cohabitee | Lives with others |
| 1488623014 | Cohabitee | Lives with others |
| 412134015 | Cohabitee left home | Lives with others |
| 285433016 | Cohabitee made appointment | Lives with others |
| 442161011 | Cohabitee returned | Lives with others |
| 103191012 | Companion | Lives with others |
| 14281911000006115 | Death location type code (actual) : Care home with nursing | Lives in care facility |
| 14281921000006111 | Death location type code (actual) : Care home without nursing | Lives in care facility |
| 283813014 | Delayed discharge to nursing home | Lives in care facility |
| 14130291000006116 | Died in care home | Lives in care facility |
| 14130311000006117 | Died in hospice | Lives in care facility |
| 14130331000006111 | Died in nursing home | Lives in care facility |
| 13996321000006115 | Died in residential home | Lives in care facility |
| 1951031000006114 | Discharge to care home | Lives in care facility |
| 449808016 | Discharge to community hospital | Lives in care facility |
| 5959601000006113 | Discharge to long stay hospital | Lives in care facility |
| 449801010 | Discharge to nursing home | Lives in care facility |
| 5959551000006111 | Discharge to private nursing home | Lives in care facility |
| 5959521000006119 | Discharge to private residential home | Lives in care facility |
| 449798016 | Discharge to residential home | Lives in care facility |
| 250535019 | Divorced couple sharing house | Lives with others |
| 250596015 | Elderly relative lives with family | Lives with others |
| 7224961000006115 | Fall in nursing home | Lives in care facility |
| 264938019 | Full care by hospice | Lives in care facility |
| 423255018 | Hospice | Lives in care facility |
| 14673861000006116 | Hospice acquired pressure injury | Lives in care facility |
| 6443301000006119 | Hospice care | Lives in care facility |
| 6443331000006110 | Hospice care management | Lives in care facility |
| 2338191000000117 | Hospice community lodge | Lives in care facility |
| 291519013 | In prison | Lives with others |
| 781861000006110 | Independent housing, not alone | Lives with others |
| 3411661000006114 | Intermediate care hospital | Lives in care facility |
| 1979651000006111 | Intervention setting: Prison setting | Lives with others |
| 4924261000006119 | Lives as companion | Lives with others |
| 1009711000006117 | Lives at other family carer's home | Lives with others |
| 250580018 | Lives in a bedsit | Lives with others |
| 4924301000006111 | Lives in a commune | Lives with others |
| 250560019 | Lives in a nursing home | Lives in care facility |
| 1009771000006114 | Lives in a registered adult care home | Lives in care facility |
| 1488625019 | Lives in a residential home | Lives in care facility |
| 738781000006113 | Lives in an old peoples home | Lives in care facility |
| 250563017 | Lives in an old peoples home | Lives in care facility |
| 406371000000117 | Lives in care home | Lives in care facility |
| 1859761000006111 | Lives in hospice | Lives in care facility |
| 4925351000006111 | Lives in hospital | Lives in care facility |
| 1859711000006113 | Lives in independent hospital/clinic | Lives in care facility |
| 4535231000006118 | Lives in lodgings | Lives with others |
| 15075091000006118 | Lives in nursing home | Lives in care facility |
| 1991811000006115 | Lives in secure hospital | Lives in care facility |
| 4925341000006114 | Lives in staffed home | Lives in care facility |
| 1009731000006111 | Lives independently with dependent children | Lives with others |
| 1009721000006113 | Lives independently with spouse/partner | Lives with others |
| 1009741000006118 | Lives independently with spouse/partner + children | Lives with others |
| 4924111000006111 | Lives with | Lives with others |
| 2224061000000114 | Lives with adoptive parents | Lives with others |
| 2224301000000113 | Lives with biological parent and step parent | Lives with others |
| 2224501000000116 | Lives with biological parents | Lives with others |
| 2266021000000119 | Lives with carer | Lives with others |
| 4924151000006112 | Lives with children | Lives with others |
| 450284014 | Lives with companion | Lives with others |
| 4924161000006114 | Lives with daughter | Lives with others |
| 337103016 | Lives with family | Lives with others |
| 408181000000117 | Lives with father | Lives with others |
| 337102014 | Lives with friend | Lives with others |
| 4924121000006115 | Lives with friends | Lives with others |
| 337115017 | Lives with grandfather | Lives with others |
| 337116016 | Lives with grandmother | Lives with others |
| 4924221000006113 | Lives with grandparents | Lives with others |
| 6767621000006110 | Lives with husband | Lives with others |
| 1176951000000115 | Lives with immunocompromised person | Lives with others |
| 337117013 | Lives with lodger | Lives with others |
| 408161000000114 | Lives with mother | Lives with others |
| 1009751000006116 | Lives with others: supported group living | Lives with others |
| 4924181000006116 | Lives with parents | Lives with others |
| 2470673016 | Lives with partner | Lives with others |
| 14072271000006112 | Lives with relative | Lives with others |
| 250595016 | Lives with relatives | Lives with others |
| 2774284015 | Lives with roommate | Lives with others |
| 4924171000006119 | Lives with son | Lives with others |
| 2881789016 | Lives with spouse | Lives with others |
| 7342331000006113 | Lives with wife | Lives with others |
| 460973012 | Living in residential institution | Lives in care facility |
| 2743651000000110 | Living temporarily with friends | Lives with others |
| 6723681000006110 | Living temporarily with relatives | Lives with others |
| 4928691000006116 | Living with carer | Lives with others |
| 8155211000006116 | Local authority and registered residential mental health care - 24 hour not intensive | Lives in care facility |
| 3016541000006111 | Long stay hospital | Lives in care facility |
| 736561000006112 | Long stay hospital inpatient | Lives in care facility |
| 250569018 | Long stay hospital inpatient | Lives in care facility |
| 1136671000000115 | Medically fit for prison transfer | Lives with others |
| 250525013 | Multiple occupancy | Lives with others |
| 3187751000006110 | NH - Nursing home | Lives in care facility |
| 8178471000006115 | NHS mental health nursing home/residential care | Lives in care facility |
| 8137181000006113 | Non NHS registered mental health nursing home/residential care | Lives in care facility |
| 8171261000006117 | Non NHS registered nursing home mental health care for older adults - 24 hour not intensive | Lives in care facility |
| 71184010 | Nursing home | Lives in care facility |
| 14673881000006114 | Nursing home acquired pressure injury | Lives in care facility |
| 1647741000000115 | Nursing home acquired pressure ulcer | Lives in care facility |
| 62241000000112 | Nursing home visit note | Lives in care facility |
| 296351000006117 | Nursing or other home | Lives in care facility |
| 5385771000006114 | Old peoples' home | Lives in care facility |
| 39471000006111 | Other accident due to fire in other residential home | Lives in care facility |
| 1488500019 | Other residential care home man voluntary/private agents | Lives in care facility |
| 1488499011 | Other residential care homes managed by local authority | Lives in care facility |
| 1488495017 | Other voluntary or private hospital or nursing home | Lives in care facility |
| 1488495017 | Other voluntary or private hospital or nursing home | Lives in care facility |
| 291210013 | Overcrowded in house | Lives with others |
| 1552711000000110 | Patient died in care home | Lives in care facility |
| 457265014 | Patient died in hospice | Lives in care facility |
| 2386971000000112 | Patient died in hospice community lodge | Lives in care facility |
| 1778951000006117 | Patient died in NHS hospice/specialist palliative care unit | Lives in care facility |
| 284635010 | Patient died in nursing home | Lives in care facility |
| 8266791000006118 | Patient died in residential home | Lives in care facility |
| 241371000006111 | Patient died in residential institution | Lives in care facility |
| 1778961000006115 | Patient died in voluntary hospice/specialist palliative care ut. | Lives in care facility |
| 8074901000006115 | Patient transfer to nursing home | Lives in care facility |
| 8074941000006118 | Patient transfer to residential home | Lives in care facility |
| 11932201000006115 | Patient unavailable to attend psychological therapy - in prison | Lives with others |
| 227981000006119 | Place of occurrence of accident or poisoning, old people's home | Lives in care facility |
| 328506012 | Place of occurrence of accident or poisoning, prison | Lives with others |
| 226751000006110 | Place of occurrence of accident or poisoning, residential institution | Lives in care facility |
| 8455031000006116 | Place of occurrence of injury is prison | Lives with others |
| 1877621000006118 | Prescription request by residential institution | Lives in care facility |
| 8260001000006113 | Prison first reception health assessment completed | Lives with others |
| 265577019 | Prison medical examination | Lives with others |
| 8221171000006119 | Prisoner | Lives with others |
| 5997731000006114 | Private nursing home | Lives in care facility |
| 5997721000006111 | Private residential home | Lives in care facility |
| 8054021000006111 | Provision of continuing care in hospital or care home | Lives in care facility |
| 530521000000114 | Provision of continuing care in nursing home | Lives in care facility |
| 1583741000006116 | Provision of continuing care in nursing home | Lives in care facility |
| 8053891000006119 | Provision of intermediate care in care home or hospital | Lives in care facility |
| 8053981000006112 | Provision of local authority permanent residential care | Lives in care facility |
| 8053871000006115 | Provision of nursing home care | Lives in care facility |
| 8053921000006113 | Provision of palliative care in hospital, hospice or care home | Lives in care facility |
| 8053991000006110 | Provision of private or voluntary permanent residential care | Lives in care facility |
| 8053841000006111 | Provision of residential care | Lives in care facility |
| 1850961000006119 | Reason for influenza vaccine - living in long stay care facility | Lives in care facility |
| 940661000006117 | Refer to doctor at prisoner's request | Lives with others |
| 449142011 | Referral to hospice | Lives in care facility |
| 1961541000006115 | Referred by care home | Lives in care facility |
| 2587751000000117 | Referred by care home | Lives in care facility |
| 8280231000006113 | Referred by nursing home | Lives in care facility |
| 2176711000000117 | Referred by residential home | Lives in care facility |
| 8220191000006110 | Residential care | Lives in care facility |
| 1563511000006112 | Residential Home | Lives in care facility |
| 1563731000006111 | Residential Home | Lives in care facility |
| 5386211000006116 | Residential home | Lives in care facility |
| 8233901000006112 | Residential home acquired pressure ulcer | Lives in care facility |
| 1219123014 | Residential institution | Lives in care facility |
| 8112831000006110 | Seen by member of prison inreach mental health team | Lives with others |
| 448496013 | Seen in hospice | Lives in care facility |
| 285209012 | Seen in nursing home | Lives in care facility |
| 411631010 | Seen in old people's home | Lives in care facility |
| 412131011 | Separated from cohabitee | Lives with others |
| 4928711000006118 | Staying with carer | Lives with others |
| 284134010 | Urgent admission to hospice | Lives in care facility |
| 216617011 | Independent housing, lives alone | Lives alone |
| 291148014 | Lives alone | Lives alone |
| 1939021000006111 | Lives alone | Lives alone |
| 852841000006110 | Lives alone | Lives alone |
| 250547013 | Lives alone - help available | Lives alone |
| 250549011 | Lives alone needs housekeeper | Lives alone |
| 13788921000006113 | Lives alone no help available | Lives alone |
| 250548015 | Lives alone -no help available | Lives alone |
| 1009761000006119 | Lives alone: supported individual living | Lives alone |
| 14281691000006116 | Person relationship (main carer) : None - lives alone | Lives alone |
| 1227733019 | [V]Hobo | Homeless |
| 1227730016 | [V]Tramp | Homeless |
| 1872221000006118 | Accom status - homeless | Homeless |
| 1872251000006110 | Accom status - night shelter/emergency or direct access hostel | Homeless |
| 1872291000006116 | Accom status - other homeless | Homeless |
| 2002921000006119 | Assertive outreach in the care of the homeless | Homeless |
| 2004001000006118 | Declined referral to homeless advocacy service | Homeless |
| 6260921000006111 | Feature of homelessness | Homeless |
| 959371000006114 | Homeless | Homeless |
| 485356010 | Homeless | Homeless |
| 226561000000111 | Homeless enhanced service completed | Homeless |
| 226421000000116 | Homeless enhanced services administration | Homeless |
| 169620017 | Homeless family | Homeless |
| 6515031000006113 | Homeless mental health care | Homeless |
| 6519991000006113 | Homeless mental health care - 3-5 contacts/week | Homeless |
| 6519981000006110 | Homeless mental health care - Daily intensive | Homeless |
| 6519951000006119 | Homeless mental health care - Full day : day care | Homeless |
| 6519961000006117 | Homeless mental health care - Part day : day care | Homeless |
| 250503019 | Homeless single person | Homeless |
| 4931071000006116 | Hostel for the homeless | Homeless |
| 14459501000006117 | Housing instability following recent homelessness | Homeless |
| 757521000000112 | Length of time homeless | Homeless |
| 397758011 | Lives in squat | Homeless |
| 337216012 | Living rough | Homeless |
| 2743791000000114 | Provision of community outreach care for homeless | Homeless |
| 1951101000006119 | Referral to day centre for homeless | Homeless |
| 2733781000000113 | Referral to homeless advocacy service | Homeless |
| 2002641000006117 | Referral to homeless advocacy service | Homeless |
| 2733941000000115 | Referral to homeless advocacy service declined | Homeless |
| 1950961000006110 | Referral to homeless team | Homeless |
| 1951111000006116 | Referral to support worker for homeless | Homeless |
| 8452221000006119 | Referred by homeless drop-in centre | Homeless |
| 12619001000006114 | Signposting to homeless support service | Homeless |
| 337220011 | Sleeping in night shelter | Homeless |
| 250506010 | Tramp | Homeless |
| 2286501000000119 | Under care of homeless advocacy service | Homeless |
| 2286501000000119 | Under care of homeless advocacy service | Homeless |
| 250508011 | Vagrant | Homeless |
| Table S13. EMIS® consultation source identifier (conssourceid in consultation files) 8400 Nursing home  8703 Nursing home visit note  9060 Residential home  37436 Residential Home  90268 Residential Home  9061 Residential home visit note  98289 Telephone, Nursing home visit  101218 Telephone, Residential Home Visit Table S14. HES admission source codes (ADMISOURC – source of admission in HES APC) 54 NHS run care home (retired in 2022-23) 55 Care Home with Nursing 56 Care Home without Nursing 69 Local authority home or care (1989-90 to 1995-96) 85 Non-NHS (other than local authority) run care home (retired in 2022-23) 86 Non-NHS (other than local authority) run nursing home 88 Hospice | | |

## Table S15. Medical codes used to determine marital status. For those who were married or in a partnership, their living situation was assumed to be 'lives with others'.

| **MedCodeId** | **Term** | **Marital Status** | **Living situation** |
| --- | --- | --- | --- |
| 1227741019 | [V]Divorce | Divorced/Separated |  |
| 460981013 | [V]Marital problems | Married/Partner | Lives with others |
| 1817361000006114 | [X]Maltreatment, by spouse or partner | Married/Partner | Lives with others |
| 335218014 | [X]Neglect and abandonment, by spouse or partner | Married/Partner | Lives with others |
| 335225019 | [X]Other maltreatment syndromes, by spouse or partner | Married/Partner | Lives with others |
| 5170631000006111 | Abuse of partner | Married/Partner | Lives with others |
| 4082271000006115 | Abusive emotional relationship | Married/Partner | Lives with others |
| 4082291000006119 | Abusive emotional relationship with spouse | Married/Partner | Lives with others |
| 250769015 | Affair ended | Married/Partner | Lives with others |
| 397777013 | Affair started | Married/Partner | Lives with others |
| 250770019 | Affair unsatisfactory | Married/Partner | Lives with others |
| 14130591000006118 | Alcoholic husband | Married/Partner | Lives with others |
| 213682015 | Alcoholic spouse | Married/Partner | Lives with others |
| 14130601000006114 | Alcoholic spouse | Married/Partner | Lives with others |
| 759671000000116 | Antenatal screening for partner required | Married/Partner | Lives with others |
| 759611000000114 | Antenatal screening not indicated for partner | Married/Partner | Lives with others |
| 493941000006111 | Artificial insemin by husband | Married/Partner | Lives with others |
| 460520016 | Artificial insemination by husband | Married/Partner | Lives with others |
| 13980011000006113 | At increased risk of intimate partner violence | Married/Partner | Lives with others |
| 7609861000006115 | At risk of intimate partner abuse | Married/Partner | Lives with others |
| 324767013 | Battered husband | Married/Partner | Lives with others |
| 250676018 | Battered husband - history | Married/Partner | Lives with others |
| 324766016 | Battered wife | Married/Partner | Lives with others |
| 250801010 | Boyfriend arrested | Married/Partner |  |
| 250733011 | Boyfriend relationship problem | Married/Partner |  |
| 298041000000110 | Breast lump detected by partner | Married/Partner | Lives with others |
| 397767011 | Broken with partner | Married/Partner |  |
| 6557241000006111 | Cancer care plan discussed with partner in relationship | Married/Partner | Lives with others |
| 6557191000006111 | Cancer diagnosis discussed with partner in relationship | Married/Partner | Lives with others |
| 7048041000006116 | Cared for by spouse | Married/Partner | Lives with others |
| 1488623014 | Cohabitee | Married/Partner | Lives with others |
| 412134015 | Cohabitee left home | Married/Partner |  |
| 285433016 | Cohabitee made appointment | Married/Partner | Lives with others |
| 442161011 | Cohabitee returned | Married/Partner | Lives with others |
| 487634016 | Cohabiting | Married/Partner | Lives with others |
| 476009017 | Common law partnership | Married/Partner | Lives with others |
| 1672431000006114 | Common law partnership | Married/Partner | Lives with others |
| 250209016 | Common-law husband | Married/Partner | Lives with others |
| 250210014 | Common-law wife | Married/Partner | Lives with others |
| 1747781000006111 | Concern expressed by spouse | Married/Partner | Lives with others |
| 251136017 | Crime against spouse | Married/Partner | Lives with others |
| 2575940016 | Death of husband | Widowed |  |
| 11927261000006115 | Death of husband | Widowed |  |
| 216633014 | Death of partner | Widowed |  |
| 1484966014 | Death of wife | Widowed |  |
| 250962016 | Disabled spouse | Married/Partner | Lives with others |
| 8309041000006110 | Discussion about relationship | Married/Partner | Lives with others |
| 5888151000006114 | Disharmony with partner | Married/Partner | Lives with others |
| 105113011 | Divorce | Divorced/Separated |  |
| 412088010 | Divorce problems | Divorced/Separated |  |
| 338024013 | Divorce proceedings | Divorced/Separated |  |
| 250645012 | Divorce proceedings pending | Divorced/Separated |  |
| 191517011 | Divorced | Divorced/Separated |  |
| 250535019 | Divorced couple sharing house | Divorced/Separated | Lives with others |
| 494031000000110 | Divorced/person whose civil partnership has been dissolved | Divorced/Separated |  |
| 5903761000006111 | Employment circumstances of partner or spouse | Married/Partner | Lives with others |
| 3392411000006112 | Engaged to be married | Married/Partner | Lives with others |
| 250764013 | Extra-marital problems | Married/Partner | Lives with others |
| 250778014 | Extra-marital problems NOS | Married/Partner | Lives with others |
| 6853791000006115 | Failure to conceive due to infertility of male partner | Married/Partner | Lives with others |
| 1834061000006113 | Female partner | Married/Partner | Lives with others |
| 300251000000110 | Fertility problems in partner | Married/Partner | Lives with others |
| 250143018 | FH: Boyfriend | Married/Partner |  |
| 12716291000006112 | FH: Boyfriend | Married/Partner |  |
| 250144012 | FH: Girlfriend | Married/Partner |  |
| 12716301000006113 | FH: Girlfriend | Married/Partner |  |
| 250783018 | First relationship | Married/Partner |  |
| 250802015 | Girlfriend arrested | Married/Partner |  |
| 250734017 | Girlfriend relationship problem | Married/Partner |  |
| 411077019 | Has infirm partner | Married/Partner | Lives with others |
| 7691581000006111 | Health of partner | Married/Partner | Lives with others |
| 250503019 | Homeless single person | Single |  |
| 250168016 | House husband | Married/Partner | Lives with others |
| 828641000006113 | Housewife | Married/Partner | Lives with others |
| 1136015 | Husband | Married/Partner | Lives with others |
| 418923013 | Husband alcoholic | Married/Partner | Lives with others |
| 2406881000000112 | Husband alive | Married/Partner | Lives with others |
| 4926681000006115 | Husband arrested | Married/Partner | Lives with others |
| 412100016 | Husband committed adultery | Married/Partner | Lives with others |
| 2406971000000119 | Husband deceased | Widowed |  |
| 442258018 | Husband died | Widowed |  |
| 250799012 | Husband in prison | Married/Partner |  |
| 412132016 | Husband left home | Married/Partner |  |
| 2406921000000118 | Husband unwell | Married/Partner | Lives with others |
| 2406841000000116 | Husband well | Married/Partner | Lives with others |
| 1804931000006113 | In de facto relationship | Married/Partner | Lives with others |
| 2159175019 | Informing partner | Married/Partner | Lives with others |
| 7067061000006113 | Intrauterine insemination with controlled ovarian hyperstimulation using partner sperm | Married/Partner | Lives with others |
| 1551151000006110 | Intrauterine insemination with superovulation using partner sperm | Married/Partner | Lives with others |
| 7058321000006111 | Intrauterine insemination without controlled ovarian hyperstimulation using partner sperm | Married/Partner | Lives with others |
| 1551171000006117 | Intrauterine insemination without superovulation using partner sperm | Married/Partner | Lives with others |
| 412087017 | Legal problem with divorce | Divorced/Separated |  |
| 3090441000006111 | Legally married | Married/Partner | Lives with others |
| 1009721000006113 | Lives independently with spouse/partner | Married/Partner | Lives with others |
| 1009741000006118 | Lives independently with spouse/partner + children | Married/Partner | Lives with others |
| 6767621000006110 | Lives with husband | Married/Partner | Lives with others |
| 2470673016 | Lives with partner | Married/Partner | Lives with others |
| 2881789016 | Lives with spouse | Married/Partner | Lives with others |
| 7342331000006113 | Lives with wife | Married/Partner | Lives with others |
| 412031013 | Looks after chronically sick husband | Married/Partner | Lives with others |
| 412033011 | Looks after chronically sick spouse | Married/Partner | Lives with others |
| 412034017 | Looks after chronically sick wife | Married/Partner | Lives with others |
| 300231000000115 | Low sperm count in partner | Married/Partner | Lives with others |
| 852911000006116 | Mad wife | Married/Partner | Lives with others |
| 486188017 | Maladjustment to married life | Married/Partner | Lives with others |
| 1834051000006111 | Male partner | Married/Partner | Lives with others |
| 250650018 | Marital breakdown | Married/Partner | Lives with others |
| 65545019 | Marital conflict | Married/Partner | Lives with others |
| 1207808017 | Marital counselling | Married/Partner | Lives with others |
| 923471000006119 | Marital counselling | Married/Partner | Lives with others |
| 717161000006113 | Marital discord | Married/Partner | Lives with others |
| 717171000006118 | Marital disharmony | Married/Partner | Lives with others |
| 500032013 | Marital problems | Married/Partner | Lives with others |
| 8111491000006116 | Marital psychotherapy | Married/Partner | Lives with others |
| 290530016 | Marital reconciliation | Married/Partner | Lives with others |
| 194168015 | Marital status |  |  |
| 717221000006112 | Marital stress | Married/Partner | Lives with others |
| 717231000006110 | Marital trouble | Married/Partner | Lives with others |
| 191514016 | Married | Married/Partner | Lives with others |
| 493971000000110 | Married/civil partner | Married/Partner | Lives with others |
| 412098016 | Mistress taken | Married/Partner | Lives with others |
| 4380351000006116 | Never married | Single |  |
| 1539061000006113 | New partner male | Married/Partner |  |
| 250782011 | New relationship | Married/Partner |  |
| 415854014 | Newly wed | Married/Partner | Lives with others |
| 262935017 | No partner at present | Single |  |
| 411070017 | Occupation of husband | Married/Partner | Lives with others |
| 397721011 | Occupation of spouse | Married/Partner | Lives with others |
| 250169012 | Occupation of spouse NOS | Married/Partner | Lives with others |
| 411071018 | Occupation of wife | Married/Partner | Lives with others |
| 412101017 | Oil rig wives syndrome | Married/Partner | Lives with others |
| 5448541000006117 | Partner | Married/Partner | Lives with others |
| 2407111000000112 | Partner alive | Married/Partner | Lives with others |
| 250791010 | Partner begins work | Married/Partner | Lives with others |
| 1661521000000116 | Partner contraception | Married/Partner | Lives with others |
| 2407071000000118 | Partner deceased | Widowed |  |
| 759851000000112 | Partner declined antenatal screening | Married/Partner | Lives with others |
| 1485014011 | Partner dying | Married/Partner | Lives with others |
| 738831000000111 | Partner had tubal ligation | Married/Partner | Lives with others |
| 13932191000006111 | Partner had vasectomy | Married/Partner | Lives with others |
| 401444017 | Partner had vasectomy | Married/Partner | Lives with others |
| 389947015 | Partner in relationship | Married/Partner | Lives with others |
| 312241000000110 | Partner is informal carer | Married/Partner | Lives with others |
| 562571000000116 | Partner pregnant | Married/Partner | Lives with others |
| 250793013 | Partner retires | Married/Partner | Lives with others |
| 411878013 | Partner sterilised | Married/Partner | Lives with others |
| 250792015 | Partner stops work | Married/Partner | Lives with others |
| 250817016 | Partner unemployed | Married/Partner | Lives with others |
| 2407151000000111 | Partner unwell | Married/Partner | Lives with others |
| 2407191000000115 | Partner well | Married/Partner | Lives with others |
| 250794019 | Partner works after retirement | Married/Partner | Lives with others |
| 412070019 | Partnership problems | Married/Partner | Lives with others |
| 1757711000006118 | Patient in a relationship | Married/Partner | Lives with others |
| 4118631000006112 | Personal relationship breakdown | Married/Partner |  |
| 6995701000006114 | Physically abusive to partner | Married/Partner | Lives with others |
| 981081000006118 | Physically/emotionally abusive to partner | Married/Partner | Lives with others |
| 1998361000006115 | Polygamous partner | Married/Partner |  |
| 6557381000006110 | Preferred place of death discussed with partner in relationship | Married/Partner | Lives with others |
| 8033011000006119 | Problem with aged spouse or partner | Married/Partner | Lives with others |
| 4064611000006116 | Psychologically abused spouse | Married/Partner | Lives with others |
| 5887191000006116 | Reconciliation with partner | Married/Partner | Lives with others |
| 283715014 | Refer to partner | Married/Partner | Lives with others |
| 5465611000006118 | Relationship | Married/Partner | Lives with others |
| 8223191000006117 | Relationship | Married/Partner | Lives with others |
| 4118641000006119 | Relationship breakdown | Married/Partner |  |
| 6025471000006110 | Relationship counseling | Married/Partner | Lives with others |
| 456915015 | Relationship counselling | Married/Partner | Lives with others |
| 14842121000006116 | Relationship problem | Married/Partner | Lives with others |
| 250732018 | Relationship problems | Married/Partner |  |
| 8097291000006118 | Relationship psychosexual therapy | Married/Partner | Lives with others |
| 415853015 | Remarried | Married/Partner | Lives with others |
| 412102012 | Row with wife | Married/Partner | Lives with others |
| 195523014 | Separated | Divorced/Separated |  |
| 412131011 | Separated from cohabitee | Divorced/Separated | Lives with others |
| 250777016 | Seven year itch - marital | Married/Partner | Lives with others |
| 84322012 | Single | Single |  |
| 397763010 | Single parent | Single |  |
| 36839019 | Single parent family | Single |  |
| 251140014 | Single parent family, father present | Single |  |
| 397807015 | Single parent family, mother present | Single |  |
| 142341000006112 | Single person | Single |  |
| 4380341000006118 | Single, never married | Single |  |
| 1135016 | Spouse | Married/Partner | Lives with others |
| 250800011 | Spouse arrested | Married/Partner | Lives with others |
| 250583016 | Spouse cannot care for patient | Married/Partner | Lives with others |
| 483248013 | Spouse committed adultery | Married/Partner | Lives with others |
| 483247015 | Spouse committed infidelity | Married/Partner | Lives with others |
| 253433011 | Spouse cooks food | Married/Partner | Lives with others |
| 169507013 | Spouse deceased | Widowed |  |
| 411044018 | Spouse haemophiliac | Married/Partner | Lives with others |
| 412104013 | Spouse inattentive | Married/Partner | Lives with others |
| 250961011 | Spouse is handicapped | Married/Partner | Lives with others |
| 412135019 | Spouse left home | Married/Partner |  |
| 7289791000006116 | Spouse of subject is victim of crime | Married/Partner | Lives with others |
| 282957016 | Spouse reassured | Married/Partner | Lives with others |
| 412089019 | Spouse returned home | Married/Partner | Lives with others |
| 4535281000006117 | Spouse unable to care for patient | Married/Partner | Lives with others |
| 412103019 | Spouse unsympathetic | Married/Partner | Lives with others |
| 397800018 | Spouse unwell | Married/Partner | Lives with others |
| 251152019 | Spouse works away from home | Married/Partner |  |
| 6916801000006110 | Victim of abusive sexual relationship with partner | Married/Partner | Lives with others |
| 7609871000006110 | Victim of intimate partner abuse | Married/Partner | Lives with others |
| 7709991000006113 | Victim of sexual assault by intimate partner | Married/Partner | Lives with others |
| 250677010 | Violent spouse | Married/Partner | Lives with others |
| 251056018 | War widows pension | Widowed |  |
| 485552011 | Widowed | Widowed |  |
| 494151000000119 | Widowed/surviving civil partner | Widowed |  |
| 129552010 | Widower | Widowed |  |
| 251052016 | Widows allowance | Widowed |  |
| 53381000006116 | Widows allowances | Widowed |  |
| 405075010 | Widows benefits | Widowed |  |
| 251057010 | Widows benefits NOS | Widowed |  |
| 4537501000006116 | Widows payment | Widowed |  |
| 251054015 | Widows pension | Widowed |  |
| 53421000006114 | Widows pensions | Widowed |  |
| 250835013 | Wife alive | Married/Partner | Lives with others |
| 4926671000006118 | Wife arrested | Married/Partner | Lives with others |
| 412099012 | Wife committed adultery | Married/Partner | Lives with others |
| 2406671000000114 | Wife deceased | Widowed |  |
| 412133014 | Wife left home | Married/Partner |  |
| 443775016 | Wife unable to cope | Married/Partner | Lives with others |
| 2406051000000112 | Wife unwell | Married/Partner | Lives with others |

## Table S16. Medical codes used to define ethnicity categories

| **MedCodeId** | **Term** | **Ethnicity category** |
| --- | --- | --- |
| 196641000006110 | RACE: Caucasian | White |
| 2537217015 | Race: White | White |
| 285925010 | White | White |
| 459726019 | White British | White |
| 459727011 | White Irish | White |
| 459728018 | Other white ethnic group | White |
| 1780407014 | White Scottish | White |
| 1780408016 | Other white British ethnic group | White |
| 285978014 | Irish (NMO) | White |
| 405070017 | Greek/Greek Cypriot (NMO) | White |
| 411594011 | Greek (NMO) | White |
| 411595012 | Greek Cypriot (NMO) | White |
| 405071018 | Turkish/Turkish Cypriot (NMO) | White |
| 411596013 | Turkish (NMO) | White |
| 411597016 | Turkish Cypriot (NMO) | White |
| 285987017 | Other European (NMO) | White |
| 459786016 | Irish traveller | White |
| 286007012 | New Zealand European | White |
| 286008019 | Pakeha | White |
| 286009010 | Other European in New Zealand | White |
| 286022019 | New Zealand ethnic group NOS | White |
| 850671000006119 | Traveller - gypsy | White |
| 1158211000000111 | Romanian | White |
| 1158301000000115 | Bulgarian | White |
| 1160331000000119 | Czech | White |
| 1551471000000116 | Slovak | White |
| 133078012 | Portuguese | White |
| 1063981000000117 | White British - ethnic category 2001 census | White |
| 141301000000110 | Irish - ethnic category 2001 census | White |
| 1064041000000111 | White Irish - ethnic category 2001 census | White |
| 141311000000112 | Other White background - ethnic category 2001 census | White |
| 157281000000117 | English - ethnic category 2001 census | White |
| 141431000000111 | Scottish - ethnic category 2001 census | White |
| 141441000000119 | Welsh - ethnic category 2001 census | White |
| 141461000000118 | Cornish - ethnic category 2001 census | White |
| 141451000000116 | Northern Irish - ethnic category 2001 census | White |
| 142691000000116 | Ulster Scots - ethnic category 2001 census | White |
| 141661000000115 | Cypriot (part not stated) - ethnic category 2001 census | White |
| 142701000000116 | Greek - ethnic category 2001 census | White |
| 142711000000119 | Greek Cypriot - ethnic category 2001 census | White |
| 156921000000110 | Turkish - ethnic category 2001 census | White |
| 142721000000113 | Turkish Cypriot - ethnic category 2001 census | White |
| 158481000000115 | Italian - ethnic category 2001 census | White |
| 138171000000114 | Irish Traveller - ethnic category 2001 census | White |
| 138181000000111 | Traveller - ethnic category 2001 census | White |
| 138191000000113 | Gypsy/Romany - ethnic category 2001 census | White |
| 138201000000110 | Polish - ethnic category 2001 census | White |
| 937301000006110 | Baltic Estonian/Latvian/Lithuanian - ethn categ 2001 census | White |
| 142741000000118 | Kosovan - ethnic category 2001 census | White |
| 138231000000116 | Albanian - ethnic category 2001 census | White |
| 142751000000115 | Bosnian - ethnic category 2001 census | White |
| 142761000000117 | Croatian - ethnic category 2001 census | White |
| 157991000000110 | Serbian - ethnic category 2001 census | White |
| 937371000006116 | Other republics former Yugoslavia - ethnic categ 2001 census | White |
| 142781000000114 | Mixed Irish and other White - ethnic category 2001 census | White |
| 937391000006115 | Oth White European/European unsp/Mixed European 2001 census | White |
| 142791000000111 | Other mixed White - ethnic category 2001 census | White |
| 937411000006115 | Other White or White unspecified ethnic category 2001 census | White |
| 2484671000000118 | White: Irish - England and Wales ethnic category 2011 census | White |
| 1968081000006112 | White: other White backgrd- Eng+Wales ethnic cat 2011 census | White |
| 2486161000000112 | White - Northern Ireland ethnic category 2011 census | White |
| 2487281000000112 | White: Scottish - Scotland ethnic category 2011 census | White |
| 2487321000000116 | White: other British - Scotland ethnic category 2011 census | White |
| 2487361000000112 | White: Irish - Scotland ethnic category 2011 census | White |
| 1968441000006112 | White: Gypsy/Irish Traveller - Scotland ethnic cat 2011 cens | White |
| 2487481000000113 | White: Polish - Scotland ethnic category 2011 census | White |
| 1968461000006111 | White: other White ethnic grp- Scotland ethnic cat 2011 cens | White |
| 6846391000006112 | Caucasoid race | White |
| 2645811000000115 | Roma ethnic group | White |
| 4740381000006118 | Other ethnic, mixed white origin | White |
| 286006015 | New Zealand ethnic groups | White |
| 937311000006113 | Commonwealth (Russian) Indep States - ethn categ 2001 census | White |
| 1968051000006116 | White:Eng/Welsh/Scot/NI/Brit - England and Wales 2011 census | White |
| 1968071000006114 | White: Gypsy/Irish Traveller - Eng+Wales eth cat 2011 census | White |
| 1968091000006110 | Mixed: White+Black Caribbean - Eng+Wales eth cat 2011 census | White |
| 1968101000006116 | Mixed: White+Black African - Eng+Wales eth cat 2011 census | White |
| 1968121000006114 | Mixed: other Mixed/multiple backgrd - Eng+Wales 2011 census | White |
| 1968251000006113 | Irish Traveller - Northern Ireland ethnic cat 2011 census | White |
| 158341000000117 | British or mixed British - ethnic category 2001 census | Mixed |
| 196631000006117 | RACE: Bangladeshi | Bandladeshi/Indian/Pakistani |
| 250228011 | Indian origin | Bandladeshi/Indian/Pakistani |
| 250231012 | West Indian origin | Bandladeshi/Indian/Pakistani |
| 250243013 | Race: West indian | Bandladeshi/Indian/Pakistani |
| 196721000006111 | RACE: Pakistani | Bandladeshi/Indian/Pakistani |
| 285954016 | Indian | Bandladeshi/Indian/Pakistani |
| 285955015 | Pakistani | Bandladeshi/Indian/Pakistani |
| 285956019 | Bangladeshi | Bandladeshi/Indian/Pakistani |
| 411579012 | West Indian (NMO) | Bandladeshi/Indian/Pakistani |
| 285976013 | Indian sub-continent (NMO) | Bandladeshi/Indian/Pakistani |
| 781081000006113 | Indian | Bandladeshi/Indian/Pakistani |
| 157271000000119 | Indian or British Indian - ethnic category 2001 census | Bandladeshi/Indian/Pakistani |
| 141361000000114 | Pakistani or British Pakistani - ethnic category 2001 census | Bandladeshi/Indian/Pakistani |
| 937541000006115 | Bangladeshi or British Bangladeshi - ethn categ 2001 census | Bandladeshi/Indian/Pakistani |
| 141521000000110 | Punjabi - ethnic category 2001 census | Bandladeshi/Indian/Pakistani |
| 141531000000112 | Kashmiri - ethnic category 2001 census | Bandladeshi/Indian/Pakistani |
| 1968131000006112 | Asian/Asian Brit: Indian - Eng+Wales ethnic cat 2011 census | Bandladeshi/Indian/Pakistani |
| 1968301000006118 | Asian or Asian British: Indian - NI ethnic cat 2011 census | Bandladeshi/Indian/Pakistani |
| 1968311000006115 | Asian/Asian British: Pakistani - NI ethnic cat 2011 census | Bandladeshi/Indian/Pakistani |
| 1968321000006111 | Asian/Asian British: Bangladeshi - NI ethnic cat 2011 census | Bandladeshi/Indian/Pakistani |
| 1968141000006119 | Asian/Asian British:Pakistani- Eng+Wales eth cat 2011 census | Bandladeshi/Indian/Pakistani |
| 1968151000006117 | Asian/Asian Brit: Bangladeshi- Eng+Wales eth cat 2011 census | Bandladeshi/Indian/Pakistani |
| 1968481000006118 | Asian: Pakistani/Pakistani Scot/Pakistani Brit- Scot 2011 | Bandladeshi/Indian/Pakistani |
| 1968491000006115 | Asian: Indian, Indian Scot/Indian Brit- Scotland 2011 census | Bandladeshi/Indian/Pakistani |
| 1968501000006111 | Bangladeshi, Bangladeshi Scot or Bangladeshi Brit- Scot 2011 | Bandladeshi/Indian/Pakistani |
| 136081000000111 | Sri Lankan - ethnic category 2001 census | Other Asian |
| 141551000000117 | Tamil - ethnic category 2001 census | Other Asian |
| 157301000000116 | Sinhalese - ethnic category 2001 census | Other Asian |
| 250224013 | Asian origin | Other Asian |
| 250230013 | Far Eastern origin | Other Asian |
| 196651000006112 | RACE: Chinese | Other Asian |
| 196661000006114 | RACE: Japanese | Other Asian |
| 196671000006119 | RACE: Korean | Other Asian |
| 196701000006118 | RACE: Oriental | Other Asian |
| 56590016 | Chinese | Other Asian |
| 411582019 | Iranian (NMO) | Other Asian |
| 285977016 | Other Asian (NMO) | Other Asian |
| 456650013 | Vietnamese | Other Asian |
| 459784018 | Other Asian ethnic group | Other Asian |
| 286018012 | South East Asian | Other Asian |
| 550541000006110 | Chinese | Other Asian |
| 286020010 | Other Asian | Other Asian |
| 1572831000000110 | Nepali | Other Asian |
| 141381000000117 | Other Asian background - ethnic category 2001 census | Other Asian |
| 141561000000119 | British Asian - ethnic category 2001 census | Other Asian |
| 937651000006117 | Other Asian or Asian unspecified ethnic category 2001 census | Other Asian |
| 141401000000117 | Chinese - ethnic category 2001 census | Other Asian |
| 141621000000111 | Vietnamese - ethnic category 2001 census | Other Asian |
| 141631000000113 | Japanese - ethnic category 2001 census | Other Asian |
| 141641000000116 | Filipino - ethnic category 2001 census | Other Asian |
| 141651000000118 | Malaysian - ethnic category 2001 census | Other Asian |
| 937941000006111 | Mauritian/Seychellois/Maldivian/St Helena eth cat 2001census | Other Asian |
| 1968161000006115 | Asian/Asian Brit: Chinese - Eng+Wales ethnic cat 2011 census | Other Asian |
| 1968331000006114 | Asian/Asian British: Chinese - NI ethnic cat 2011 census | Other Asian |
| 1968341000006116 | Asian/Asian British: other Asian - NI ethnic cat 2011 census | Other Asian |
| 1968511000006114 | Asian: Chinese - Scotland ethnic category 2011 census | Other Asian |
| 1968521000006118 | Asian: other Asian group - Scotland ethnic cat 2011 census | Other Asian |
| 1968171000006110 | Asian/Asian Brit: other Asian- Eng+Wales eth cat 2011 census | Other Asian |
| 250223019 | African origin | Black |
| 196601000006113 | RACE: Afro-caribbean | Black |
| 514611000006111 | Black Caribbean | Black |
| 30683015 | Black African | Black |
| 285931013 | Black, other, non-mixed origin | Black |
| 285932018 | Black British | Black |
| 405064011 | Black Caribbean/W.I./Guyana | Black |
| 285930014 | Black Caribbean | Black |
| 453109012 | Black West Indian | Black |
| 453110019 | Black Guyana | Black |
| 405065012 | Black N African/Arab/Iranian | Black |
| 411573013 | Black North African | Black |
| 411574019 | Black Arab | Black |
| 411575018 | Black Iranian | Black |
| 285943014 | Black - other African country | Black |
| 411577014 | Black Indo-Caribbean | Black |
| 285950013 | Black Black - other | Black |
| 285951012 | Black - other, mixed | Black |
| 405067016 | Caribbean I./W.I./Guyana (NMO) | Black |
| 411578016 | Caribbean Island (NMO) | Black |
| 411580010 | Guyana (NMO) | Black |
| 459782019 | Other black ethnic group | Black |
| 154401000000118 | Caribbean - ethnic category 2001 census | Black |
| 141391000000115 | African - ethnic category 2001 census | Black |
| 158351000000119 | Other Black background - ethnic category 2001 census | Black |
| 141591000000113 | Somali - ethnic category 2001 census | Black |
| 141601000000119 | Nigerian - ethnic category 2001 census | Black |
| 157311000000119 | Black British - ethnic category 2001 census | Black |
| 158371000000111 | Mixed Black - ethnic category 2001 census | Black |
| 937731000006115 | Other Black or Black unspecified ethnic category 2001 census | Black |
| 1968541000006113 | African: any other African - Scotland ethnic cat 2011 census | Black |
| 459730016 | Black - ethnic group | Black |
| 1968181000006113 | Black/African/Carib/Black Brit: African- Eng+Wales 2011 cens | Black |
| 1968191000006111 | Black/African/Caribbn/Black Brit: Caribbean - Eng+Wales 2011 | Black |
| 1968201000006114 | Black/Afr/Carib/Black Brit: other Black- Eng+Wales 2011 cens | Black |
| 1968351000006119 | Black/Afri/Carib/Black Brit: African- NI eth cat 2011 census | Black |
| 1968361000006117 | Black/Afri/Carib/Black Brit: Caribbean- NI eth cat 2011 cens | Black |
| 1968371000006112 | Black/Afri/Carib/Black Brit: other - NI eth cat 2011 census | Black |
| 1968531000006115 | African: African/African Scot/African Brit - Scotland 2011 | Black |
| 1968551000006110 | Carib/Black: Caribbean/Carib Scot/Carib Brit- Scotland 2011 | Black |
| 1968561000006112 | Carib/Black: Black/Black Scot/Black Brit- Scotland 2011 cens | Black |
| 1968571000006117 | Carib/Black: any other Black/Caribbean grp - Scotland 2011 | Black |
| 514651000006112 | Black E Afric Asia/Indo-Caribb | Mixed |
| 411576017 | Black East African Asian | Mixed |
| 285948017 | Black Indian sub-continent | Mixed |
| 285949013 | Black - other Asian | Mixed |
| 285952017 | Other Black - Black/White orig | Mixed |
| 285953010 | Other Black - Black/Asian orig | Mixed |
| 196681000006116 | RACE: Mixed | Mixed |
| 196611000006111 | RACE: Afro-caucasian | Mixed |
| 405069018 | E Afric Asian/Indo-Carib (NMO) | Mixed |
| 411583012 | East African Asian (NMO) | Mixed |
| 411584018 | Indo-Caribbean (NMO) | Mixed |
| 285989019 | Other ethnic, mixed origin | Mixed |
| 285990011 | Other ethnic, Black/White orig | Mixed |
| 285991010 | Other ethnic, Asian/White orig | Mixed |
| 285992015 | Other ethnic, mixed white orig | Mixed |
| 285993013 | Other ethnic, other mixed orig | Mixed |
| 460153018 | Black Caribbean and White | Mixed |
| 460154012 | Black African and White | Mixed |
| 141321000000118 | White and Black Caribbean - ethnic category 2001 census | Mixed |
| 141331000000116 | White and Black African - ethnic category 2001 census | Mixed |
| 141341000000113 | White and Asian - ethnic category 2001 census | Mixed |
| 141351000000111 | Other Mixed background - ethnic category 2001 census | Mixed |
| 141471000000113 | Black and Asian - ethnic category 2001 census | Mixed |
| 141481000000110 | Black and Chinese - ethnic category 2001 census | Mixed |
| 157291000000115 | Black and White - ethnic category 2001 census | Mixed |
| 141491000000112 | Chinese and White - ethnic category 2001 census | Mixed |
| 158361000000116 | Asian and Chinese - ethnic category 2001 census | Mixed |
| 937511000006119 | Other Mixed or Mixed unspecified ethnic category 2001 census | Mixed |
| 141541000000115 | East African Asian - ethnic category 2001 census | Mixed |
| 141571000000114 | Caribbean Asian - ethnic category 2001 census | Mixed |
| 141511000000116 | Mixed Asian - ethnic category 2001 census | Mixed |
| 1968111000006118 | Mixed: White+Asian - Eng+Wales ethnic category 2011 census | Mixed |
| 1968261000006110 | Mixed: White and Black Caribbean - NI ethnic cat 2011 census | Mixed |
| 1968271000006115 | Mixed: White and Black African - NI ethnic cat 2011 census | Mixed |
| 1968281000006117 | Mixed: White and Asian - NI ethnic category 2011 census | Mixed |
| 1968291000006119 | Mixed: other Mixed/multiple ethnic backgrd - NI 2011 census | Mixed |
| 1968471000006116 | Mixed/multiple ethnic grps: any- Scot ethnic cat 2011 census | Mixed |
| 4740361000006111 | Other ethnic, Asian/White origin | Mixed |
| 4740341000006112 | Other ethnic, Black/White origin | Mixed |
| 4740401000006118 | Other ethnic, other mixed origin | Mixed |
| 459729014 | Mixed ethnic census group | Mixed |
| 371005013 | Race: Other | Other |
| 285958018 | Other ethnic non-mixed (NMO) | Other |
| 405068014 | N African Arab/Iranian (NMO) | Other |
| 411581014 | North African Arab (NMO) | Other |
| 285988010 | Other ethnic NEC (NMO) | Other |
| 459785017 | Other ethnic group | Other |
| 286012013 | New Zealand Maori | Other |
| 507015012 | Samoan | Other |
| 286013015 | Cook Island Maori | Other |
| 504723011 | Tongan | Other |
| 286014014 | Niuean | Other |
| 286015010 | Tokelauan | Other |
| 501416013 | Fijian | Other |
| 286017019 | Other Pacific ethnic group | Other |
| 286021014 | Other New Zealand ethnic group | Other |
| 141411000000115 | Other - ethnic category 2001 census | Other |
| 142811000000112 | North African - ethnic category 2001 census | Other |
| 142851000000111 | Moroccan - ethnic category 2001 census | Other |
| 142861000000114 | Latin American - ethnic category 2001 census | Other |
| 138271000000119 | South and Central American - ethnic category 2001 census | Other |
| 142901000000119 | Any other group - ethnic category 2001 census | Other |
| 1968211000006112 | Other ethnic group: Arab - Eng+Wales ethnic cat 2011 census | Other |
| 1968221000006116 | Other ethnic: any other grp - Eng+Wales eth cat 2011 census | Other |
| 1968381000006110 | Other ethnic group: Arab - NI ethnic category 2011 census | Other |
| 1968391000006113 | Other ethnic group: any other grp- NI ethnic cat 2011 census | Other |
| 1968581000006119 | Other ethnic grp: Arab/Arab Scot/Arab British- Scotland 2011 | Other |
| 1968591000006116 | Other ethnic grp: any other ethnic grp- Scotland 2011 census | Other |
| 250229015 | Middle Eastern origin | Other |
| 196621000006115 | RACE: Arab | Other |
| 523591000000116 | Yemeni | Other |
| 138251000000111 | Arab - ethnic category 2001 census | Other |
| 142831000000116 | Israeli - ethnic category 2001 census | Other |
| 138261000000114 | Iranian - ethnic category 2001 census | Other |
| 142841000000113 | Kurdish - ethnic category 2001 census | Other |
| 937871000006114 | Mid East (excl Israeli, Iranian & Arab) - eth cat 2001 cens | Other |
