# Supplementary material for: Prevalence of living alone with dementia and other progressive neurological conditions: findings from primary care data in England
Source: BMC Med. 2025 Nov 4;23:607. doi: 10.1186/s12916-025-04443-x (PMC12584314; doi:10.1186/s12916-025-04443-x)
Supplement: Supplementary file 2 — Additional file 2. Additional methods, Table S1, Table S2, Table S3, Table S4, Table S5, Table S6. Additional methods- Acceptable’ patient criteria in CPRD. Table S1- Exploring the use of both medical codes and household number to define those living alone. Table S2- A summary of the total numbers stratified by measures of inequality, and proportion living in a care home. Table S3- Sensitivity analysis: Prevalence of living alone with dementia stratified by measures of inequality, additionally including patients identified by condition-specific medications. Table S4- Sensitivity analysis: Prevalence of living alone with Parkinson’s disease stratified by measures of inequality, additionally including patients identified by condition-specific medications. Table S5- Sensitivity analysis: Prevalence of living alone with motor neurone disease stratified by measures of inequality, additionally including patients identified by condition-specific medications. Table S6- Age and sex standardised prevalence rates of living alone with dementia or Parkinson’s disease by each upper tier local authority area. [file 12916_2025_4443_MOESM2_ESM.docx]

**Additional file 2**

**Additional methods**

*‘Acceptable’ patient criteria in CPRD*

Patients are labelled as ‘acceptable’ for use in research by a process that identifies and excludes patients with non-continuous follow up or patients with poor data recording that raises suspicion as to the validity of that patient’s record. Patient data is checked for the following issues:

- Year of birth is empty
- Current registration date is empty
- Current registration date is greater than the practice’s last collection date
- Current registration date is less than or equal to 01/01/1900
- Current registration date is equal to or greater than the registration end date
- Current registration date is prior to the birth year
- Gender other than male, female or indeterminate
- Age is greater than 115 at end of follow-up (based on registration end date, death or last collection date)
- All recorded health care episodes have empty event dates
- All recorded health care episodes have invalid events dates (less than or equal to 01/01/1900 or greater than last collection date)
- All recorded health care episodes have dates before the birth year
- Patients are not permanently registered.

If any of these conditions are true, then the patient is labelled unacceptable and is not recommended for use in research.

**Table S1. Exploring the use of both medical codes and household number to define those living alone**

|  | Dementia | | Parkinson’s disease | | Motor neurone disease | | Huntington’s disease | |
| --- | --- | --- | --- | --- | --- | --- | --- | --- |
|  | % with living situation | % living alone | % with living situation | % living alone | % with living situation | % living alone | % with living situation | % living alone |
| Medical codes only | 67.5% | 27.7% | 45.9% | 17.6% | 38.4% | 14.4% | 48.0% | 15.2% |
| Medical codes plus household number to define living with others | 83.3% | 17.4% | 76.6% | 8.7% | 74.1% | 5.8% | 75.0% | 6.7% |
| Medical codes plus household number to define both living alone and living with others | 99.3% | 39.9% | 98.8% | 33.1% | 98.3% | 33.0% | 98.8% | 38.1% |

.

**Table S2. A summary of the total numbers stratified by measures of inequality, and proportion living in a care home**

|  | Dementia | | Parkinson’s disease | | Motor neurone disease | | Huntington’s disease | |
| --- | --- | --- | --- | --- | --- | --- | --- | --- |
|  | Total (N) | Lives in a care facility  (N, %) | Total (N) | Lives in a care facility  (N, %) | Total (N) | Lives in a care facility  (N, %) | Total (N) | Lives in a care facility  (N, %) |
| Total | 204010 | 83372 (40.9%) | 39753 | 6419 (16.1%) | 3456 | 500 (14.5%) | 1649 | 489 (29.7%) |
| Sex  Male  Female | 81155  122855 | 28124 (34.7%)  55248 (45.0%) | 23569  16184 | 3488 (14.8%)  2931 (18.1%) | 2043  1413 | 282 (13.8%)  218 (15.4%) | 790  859 | 227 (28.7%)  262 (30.5%) |
| Age  Group 1 (oldest)  Group 2  Group 3  Group 4  Group 5  Group 6 | 136387  31784  15803  8553  11483  - | 64176 (47.1%)  10412 (32.8%)  4382 (27.7%)  2081 (24.3%)  2321 (20.2%)  - | 15788  9063  6118  3854  3848  1082 | 4028 (25.5%)  1275 (14.1%)  639 (10.4%)  254 (6.6%)  191 (5.0%)  32 (3.0%) | 873  796  672  401  714  - | 207 (23.7%)  136 (17.1%)  91 (13.5%)  40 (10.0%)  26 (3.6%)  - | 200  324  476  332  317  - | 67 (33.5%)  120 (37.0%)  172 (36.1%)  82 (24.7%)  48 (15.1%)  - |
| Region  North East  North West  Yorkshire and The Humber  East Midlands  West Midlands  East of England  London  South East  South West | 8071  44006  4329  3272  39554  8045  28758  45431  22544 | 4052 (50.2%)  18113 (41.2%)  1678 (38.8%)  1220 (37.3%)  16414 (41.5%)  3045 (37.8%)  8600 (29.9%)  20113 (44.3%)  10137 (45.0%) | 1705  7622  942  662  7246  1763  5342  9797  4674 | 287 (16.8%)  1232 (16.2%)  139 (14.8%)  89 (13.4%)  1302 (18.0%)  242 (13.7%)  624 (11.7%)  1665 (17.0%)  839 (18.0%) | 142  675  92  699  152  582  794  320 | 19 (13.4%)  97 (14.4%)  10 (10.9%)  104 (14.9%)^  30 (19.7%)  66 (11.3%)  111 (14.0%)  63 (19.7%) | -  -  -  -  -  -  -  - | -  -  -  -  -  -  -  - |
| Deprivation  Quintile 1 (least)  Quintile 2  Quintile 3  Quintile 4  Quintile 5 (most)  Missing | 29101  27441  24998  23923  21917  76630 | 8626 (29.6%)  8395 (30.6%)  7479 (29.9%)  6531 (27.3%)  6462 (29.5%)  45879 | 7498  6499  5271  4639  3387  12459 | 718 (9.6%)  603 (9.3%)  483 (9.2%)  485 (10.5%)  335 (9.9%)  3795 | 533  504  480  471  435  1033 | 71 (13.3%)  69 (13.7%)  54 (11.3%)  50 (10.6%)  42 (9.7%)  214 | 196  197  235  247  216  558 | 30 (15.3%)  49 (24.9%)  61 (26.0%)  68 (27.5%)  42 (19.4%)  239 |
| Urban/rural  Urban  Rural  Missing | 144509  20697  38804 | 57728 (39.9%)  8969 (43.3%)  16675 | 27167  5102  7848 | 4407 (16.2%)  730 (14.3%)  1282 | 2447  389  620 | 350 (14.3%)  56 (14.4%)  94 | 1138  187  324 | 313 (27.5%)  60 (32.1%)  116 |
| Ethnicity  White  South Asian  Chinese/Other Asian  Black  Mixed  Other  Missing | 182143  7451  2284  5704  996  2186  3246 | 78040 (42.8%)  928 (12.5%)  417 (18.3%)  1288 (22.6%)  291 (29.2%)  548 (25.1%)  1860 | 35899  1330  610  715  193  420  588 | 6012 (16.7%)  86 (6.5%)  50 (8.2%)  66 (9.2%)  26 (13.5%)  33 (7.9%)  146 | 2897  185  79  118  49  79  56 | 439 (15.2%)  14 (7.6%)  6 (7.6%)  17 (14.4%)  7 (14.3%)  10 (12.7%)  7 | -  -  -  -  -  - | -  -  -  -  -  - |

*Notes:* Age groups: dementia - group 1 = 80+, group 2 = 75-79, group 3 = 70-74, group 4 = 65-69, group 5 = <65; Parkinson’s disease - group 1 = 80+, group 2 = 75-79, group 3 = 70-74, group 4 = 65-69, group 5 = <55-65, group 6 = <55; motor neurone disease - group 1 = 75+, group 2 = 65-74, group 3 = 55-64, group 4 = 45-54, group 5 = <45. ^East Midlands and West Midlands were combined for motor neurone disease due to small numbers.

**Table S3. Sensitivity analysis: Prevalence of living alone with dementia stratified by measures of inequality, additionally including patients identified by condition-specific medications**

|  | Lives alone (N) | Lives with others (N) | Prevalence living alone % (95% CI) | Lives alone vs lives with others  RR (95% CI) |
| --- | --- | --- | --- | --- |
| Total | 49129 | 74293 | 39.8% (39.5 – 40.1) | - |
| Sex  Male  Female | 31029  18100 | 38003  36290 | 33.3% (32.9 – 33.7)  44.9% (44.6 – 45.3) | 0.75 (0.74 – 0.76)  Ref |
| Age  80+  75-79  70-74  65-69  <65 | 31869  7455  4003  2360  3442 | 41579  14516  7784  4332  6082 | 43.4% (43.0 – 43.7)  33.9% (33.3 – 34.6)  34.0% (33.1 – 34.8)  35.3% (34.1 – 36.4)  36.1% (35.2 – 37.1) | Ref  0.79 (0.74 – 0.75)  0.79 (0.77 – 0.81)  0.83 (0.80 – 0.85)  0.83 (0.81 – 0.86) |
| Region  North East  North West  Yorkshire and The Humber  East Midlands  West Midlands  East of England  London  South East  South West | 1762  11072  942  775  9316  1922  8685  9958  4697 | 2381  15341  1762  1322  14076  3178  12063  16125  8045 | 42.5% (41.0 – 44.0)  41.9% (41.3 – 42.5)  34.8% (33.0 – 36.6)  37.0% (34.9 – 39.0)  39.8% (39.2 – 40.5)  37.7% (36.4 – 39.0)  41.9% (41.2 – 42.5)  38.2% (37.6 – 38.8)  36.9% (36.0 – 37.7) | 1.12 (1.08 – 1.16)  1.12 (1.10 – 1.14)  0.93 (0.88 – 0.98)  1.00 (0.94 – 1.05)  1.06 (1.03 – 1.08)  1.00 (0.96 – 1.04)  1.09 (1.07 – 1.11)  Ref  0.97 (0.95 – 1.00) |
| Deprivation  Quintile 1 (least)  Quintile 2  Quintile 3  Quintile 4  Quintile 5 (most)  Missing | 7393  7413  6997  7597  7260  12469 | 13645  12061  10924  10136  8476  19051 | 35.1% (34.5 – 35.8)  38.1% (37.4 – 38.7)  39.0% (38.3 – 39.8)  42.8% (42.1 – 43.6)  46.1% (45.4 – 46.9) | Ref  1.08 (1.06 – 1.11)  1.12 (1.09 – 1.15)  1.22 (1.19 – 1.25)  1.33 (1.29 – 1.36) |
| Urban/rural  Urban  Rural  Missing | 36043  4117  8969 | 52696  7933  13664 | 40.6% (40.3 – 40.9)  34.2% (33.3 – 35.0) | Ref  0.75 (0.74 – 0.76) |
| Ethnicity  White  Bangladeshi/Indian/Pakistani  Other Asian  Black  Mixed  Other  Missing | 43340  1621  518  2144  312  603  591 | 63172  5014  1405  2350  422  1076  854 | 40.7% (40.4 – 41.0)  24.4% (23.4 – 25.5)  26.9% (25.0 – 28.9)  47.7% (46.2 – 49.2)  42.5% (38.9 – 46.1)  35.9% (33.6 – 38.2) | Ref  0.62 (0.59 – 0.64)  0.68 (0.63 – 0.73)  1.13 (1.10 – 1.17)  1.06 (0.97 – 1.15)  0.90 (0.84 – 0.96) |

*Notes:* RR, risk ratio. CI, confidence intervals. Analyses were adjusted for age band and sex.

**Table S4. Sensitivity analysis: Prevalence of living alone with Parkinson’s disease stratified by measures of inequality, additionally including patients identified by condition-specific medications**

|  | Lives alone (N) | Lives with others (N) | Prevalence living alone % (95% CI) | Lives alone vs lives with others  RR (95% CI) |
| --- | --- | --- | --- | --- |
| Total | 31127 | 60949 | 33.8% (33.5 – 34.1) | - |
| Sex  Male  Female | 13818  17309 | 29163  31786 | 32.1% (31.7 – 32.6)  35.3% (34.8 – 35.7) | 0.88 (0.86 – 0.90)  Ref |
| Age  80+  75-79  70-74  65-69  55-64  <55 | 10090  4968  3809  2993  4874  4393 | 14725  10467  8049  6507  10234  10967 | 40.7% (40.0 – 41.3)  32.2% (31.4 – 32.9)  32.1% (31.3 – 33.0)  31.5% (30.6 – 32.4)  32.3% (31.5 – 33.0)  28.6% (27.9 – 29.3) | Ref  0.79 (0.77 – 0.81)  0.79 (0.76 – 0.81)  0.77 (0.74 – 0.79)  0.78 (0.76 – 0.20)  0.68 (0.66 – 0.70) |
| Region  North East  North West  Yorkshire and The Humber  East Midlands  West Midlands  East of England  London  South East  South West | 1580  8248  747  443  6151  1075  3523  6131  3229 | 2893  14771  1867  1151  11140  2409  5972  13394  7352 | 35.3% (33.9 – 36.7)  35.8% (35.2 – 36.5)  28.6% (26.8 – 30.3)  27.8% (25.6 – 30.0)  35.6% (34.9 – 36.3)  30.9% (29.3 – 32.4)  37.1% (36.1 – 38.1)  31.4% (30.7 – 32.1)  30.5% (29.6 – 31.4) | 1.14 (1.10 – 1.19)  1.16 (1.13 – 1.19)  0.92 (0.86 – 0.98)  0.89 (0.82 – 0.97)  1.13 (1.10 – 1.16)  0.98 (0.93 – 1.03)  1.18 (1.14 – 1.22)  Ref  0.98 (0.94 – 1.01) |
| Deprivation  Quintile 1 (least)  Quintile 2  Quintile 3  Quintile 4  Quintile 5 (most)  Missing | 4320  4620  4363  4693  5251  7880 | 10261  10149  8609  8272  8073  15585 | 29.6% (28.9 – 30.4)  31.3% (30.5 – 32.0)  33.6% (32.8 – 34.4)  36.2% (35.4 – 37.0)  39.4% (38.6 – 40.2) | Ref  1.07 (1.03 – 1.10)  1.16 (1.12 – 1.20)  1.26 (1.22 – 1.31)  1.42 (1.37 – 1.46) |
| Urban/rural  Urban  Rural  Missing | 22322  3153  5652 | 41281  8182  11486 | 35.1% (34.7 – 35.5)  27.8% (27.0 – 28.6) | Ref  0.78 (0.76 – 0.81) |
| Ethnicity  White  Bangladeshi/Indian/Pakistani  Other Asian  Black  Mixed  Other  Missing | 28834  656  239  535  164  292  407 | 55473  2362  766  656  290  623  779 | 34.2% (33.9 – 34.5)  21.7% (20.3 – 23.2)  23.8% (21.1 – 26.4)  44.9% (42.1 – 47.7)  36.1% (31.7 – 40.5)  31.9% (28.9 – 34.9) | Ref  0.65 (0.61 – 0.70)  0.72 (0.64 – 0.80)  1.29 (1.21 – 1.38)  1.11 (0.98 – 1.26)  0.97 (0.88 – 1.06) |

*Notes:* RR, risk ratio. CI, confidence intervals. Analyses were adjusted for age band and sex.

**Table S5. Sensitivity analysis: Prevalence of living alone with motor neurone disease stratified by measures of inequality, additionally including patients identified by condition-specific medications**

|  | Lives alone (N) | Lives with others (N) | Prevalence living alone % (95% CI) | Lives alone vs lives with others  RR (95% CI) |
| --- | --- | --- | --- | --- |
| Total | 984 | 1995 | 33.0% (31.3 – 34.7) | - |
| Sex  Male  Female | 613  371 | 1164  831 | 34.5% (32.3 – 36.7)  30.9% (28.3 – 33.5) | 1.17 (1.00 – 1.37)  Ref |
| Age  75+  65-74  55-64  45-54  <45 | 234  217  211  126  196 | 440  448  375  237  495 | 34.7% (31.1 – 38.3)  32.6% (29.1 – 36.2)  36.0% (32.1 – 39.9)  34.7% (29.8 – 39.6)  28.4% (25.0 – 31.7) | Ref  0.94 (0.81 – 1.09)  1.04 (0.90 – 1.21)  1.01 (0.85 – 1.20)  0.83 (0.71 – 0.97) |
| Region  North East  North West  Yorkshire and The Humber  Midlands^  East of England  London  South East  South West | 40  202  24  229  32  189  195  73 | 83  381  58  369  91  335  494  184 | 32.5% (24.2 – 40.8)  34.6% (30.8 – 38.5)  29.3% (19.4 – 39.1)  38.3% (34.4 – 42.2)  26.0% (18.3 – 33.8)  36.1% (32.0 – 40.2)  28.3% (24.9 – 31.7)  28.4% (22.9 – 33.9) | 1.15 (0.87 – 1.53)  1.23 (1.04 – 1.44)  1.02 (0.72 – 1.46)  1.37 (1.17 – 1.60)  0.93 (0.68 – 1.28)  1.32 (1.12 – 1.55)  Ref  1.00 (0.80 – 1.26) |
| Deprivation  Quintile 1 (least)  Quintile 2  Quintile 3  Quintile 4  Quintile 5 (most)  Missing | 111  127  137  164  151  294 | 357  312  291  259  243  533 | 23.7% (19.9 – 27.6)  28.9% (24.7 – 33.2)  32.0% (27.6 – 36.4)  38.8% (34.1 – 43.4)  38.3% (33.5 – 43.1) | Ref  1.20 (0.96 – 1.49)  1.36 (1.10 – 1.69)  1.66 (1.35 – 2.02)  1.67 (1.36 – 2.04) |
| Urban/rural  Urban  Rural  Missing | 724  72  188 | 1388  262  345 | 34.3% (32.3 – 36.3)  21.6% (17.1 – 26.0) | Ref  0.62 (0.50 – 0.76) |
| Ethnicity  White  Bangladeshi/Indian/Pakistani  Other Asian  Black  Mixed  Other  Missing | 816  40  15  49  16  28  20 | 1657  134  59  52  26  35  32 | 33.0% (31.1 – 34.8)  23.0% (16.7 – 29.2)  20.3% (11.1 – 29.4)  48.5% (38.8 – 58.3)  38.1% (23.4 – 52.8)  44.4% (32.2 – 56.7) | Ref  0.72 (0.55 – 0.96)  0.64 (0.40 – 1.01)  1.55 (1.26 – 1.90)  1.26 (0.85 – 1.87)  1.43 (1.09 – 1.88) |

*Notes:* RR, risk ratio. CI, confidence intervals. Analyses were adjusted for age band and sex. ^ East Midlands and West. Midlands have been combined due to small numbers, in order to minimise re-identification risk.

**Table S6. Age and sex standardised prevalence rates of living alone with dementia or Parkinson’s disease by each upper tier local authority area**

| Upper tier local authority area | Dementia | | | Parkinson's disease | | |
| --- | --- | --- | --- | --- | --- | --- |
|  | Prevalence | Lower CI | Upper CI | Prevalence | Lower CI | Upper CI |
| **North East** |  |  |  |  |  |  |
| County Durham | 41.6% | 40.0% | 43.3% | 36.7% | 34.1% | 39.2% |
| Darlington | 41.9% | 38.3% | 45.4% | 36.8% | 31.1% | 42.5% |
| Gateshead | 42.0% | 39.3% | 44.6% | 36.8% | 32.5% | 41.2% |
| Hartlepool | 41.8% | 37.9% | 45.8% | 36.7% | 30.3% | 43.0% |
| Middlesbrough | 41.9% | 38.5% | 45.3% | 36.8% | 31.2% | 42.3% |
| Newcastle upon Tyne | 42.1% | 39.6% | 44.6% | 36.8% | 32.8% | 40.9% |
| North Tyneside | 41.8% | 39.3% | 44.4% | 36.8% | 32.6% | 40.9% |
| Northumberland | 41.6% | 39.8% | 43.5% | 36.7% | 33.7% | 39.7% |
| Redcar and Cleveland | 41.7% | 38.7% | 44.7% | 36.8% | 31.9% | 41.6% |
| South Tyneside | 41.9% | 38.9% | 45.0% | 36.8% | 31.9% | 41.7% |
| Stockton-on-Tees | 41.7% | 38.9% | 44.4% | 36.7% | 32.2% | 41.1% |
| Sunderland | 41.8% | 39.5% | 44.1% | 36.8% | 33.1% | 40.4% |
| **North West** |  |  |  |  |  |  |
| Blackburn with Darwen | 41.6% | 38.3% | 44.8% | 34.4% | 28.3% | 40.5% |
| Blackpool | 41.9% | 39.1% | 44.7% | 34.7% | 29.3% | 40.0% |
| Bolton | 41.7% | 39.6% | 43.9% | 34.6% | 30.6% | 38.6% |
| Bury | 41.9% | 39.3% | 44.4% | 34.7% | 29.8% | 39.5% |
| Cheshire East | 42.0% | 40.5% | 43.6% | 34.8% | 31.8% | 37.8% |
| Cheshire West and Chester | 42.0% | 40.2% | 43.7% | 34.7% | 31.5% | 38.0% |
| Cumberland | 41.8% | 39.9% | 43.7% | 34.6% | 31.0% | 38.2% |
| Halton | 41.4% | 38.2% | 44.6% | 34.4% | 28.5% | 40.3% |
| Knowsley | 41.8% | 38.8% | 44.8% | 34.6% | 29.0% | 40.2% |
| Lancashire | 41.8% | 40.9% | 42.8% | 34.7% | 32.9% | 36.4% |
| Liverpool | 41.8% | 40.1% | 43.6% | 34.6% | 31.2% | 38.0% |
| Manchester | 41.7% | 39.5% | 43.8% | 34.4% | 30.4% | 38.3% |
| Oldham | 41.8% | 39.3% | 44.3% | 34.6% | 30.0% | 39.3% |
| Rochdale | 41.7% | 39.1% | 44.2% | 34.5% | 29.8% | 39.3% |
| Salford | 41.8% | 39.2% | 44.3% | 34.6% | 29.8% | 39.4% |
| Sefton | 42.4% | 40.5% | 44.2% | 34.9% | 31.4% | 38.5% |
| St. Helens | 41.7% | 39.3% | 44.2% | 34.6% | 30.0% | 39.3% |
| Stockport | 42.2% | 40.3% | 44.2% | 34.9% | 31.2% | 38.6% |
| Tameside | 41.6% | 39.2% | 44.1% | 34.5% | 30.0% | 39.0% |
| Trafford | 42.3% | 40.0% | 44.6% | 34.9% | 30.4% | 39.4% |
| Warrington | 41.9% | 39.5% | 44.3% | 34.7% | 30.2% | 39.2% |
| Westmorland and Furness | 41.9% | 39.9% | 43.9% | 34.7% | 30.9% | 38.5% |
| Wigan | 41.5% | 39.6% | 43.5% | 34.5% | 31.0% | 38.1% |
| Wirral | 42.1% | 40.3% | 43.9% | 34.8% | 31.4% | 38.2% |
| **Yorkshire** |  |  |  |  |  |  |
| Barnsley | 34.7% | 32.4% | 37.0% | 27.5% | 23.7% | 31.3% |
| Bradford | 35.1% | 33.4% | 36.8% | 27.6% | 24.7% | 30.5% |
| Calderdale | 34.8% | 32.3% | 37.4% | 27.5% | 23.3% | 31.7% |
| Doncaster | 34.8% | 32.8% | 36.9% | 27.6% | 24.1% | 31.0% |
| East Riding of Yorkshire | 34.9% | 33.3% | 36.6% | 27.7% | 24.9% | 30.4% |
| Kingston upon Hull, City of | 34.7% | 32.2% | 37.2% | 27.4% | 23.3% | 31.6% |
| Kirklees | 34.9% | 33.1% | 36.7% | 27.6% | 24.6% | 30.6% |
| Leeds | 35.1% | 33.7% | 36.5% | 27.7% | 25.4% | 30.0% |
| North East Lincolnshire | 34.9% | 32.2% | 37.7% | 27.7% | 23.1% | 32.3% |
| North Lincolnshire | 34.9% | 32.3% | 37.4% | 27.6% | 23.3% | 31.9% |
| North Yorkshire | 35.0% | 33.7% | 36.2% | 27.7% | 25.6% | 29.8% |
| Rotherham | 34.8% | 32.6% | 37.0% | 27.6% | 23.9% | 31.2% |
| Sheffield | 35.1% | 33.6% | 36.7% | 27.7% | 25.0% | 30.5% |
| Wakefield | 34.8% | 32.8% | 36.7% | 27.5% | 24.3% | 30.8% |
| York | 35.3% | 32.9% | 37.8% | 27.8% | 23.6% | 32.1% |
| **East Midlands** |  |  |  |  |  |  |
| Derby | 37.4% | 35.1% | 39.7% | 28.7% | 24.7% | 32.7% |
| Derbyshire | 37.0% | 35.9% | 38.2% | 28.5% | 26.6% | 30.5% |
| Leicester | 37.1% | 34.8% | 39.4% | 28.4% | 24.5% | 32.4% |
| Leicestershire | 37.1% | 35.9% | 38.4% | 28.6% | 26.5% | 30.7% |
| Lincolnshire | 37.0% | 35.9% | 38.1% | 28.6% | 26.7% | 30.5% |
| North Northamptonshire | 36.9% | 35.0% | 38.8% | 28.5% | 25.2% | 31.7% |
| Nottingham | 37.3% | 34.8% | 39.8% | 28.5% | 24.3% | 32.8% |
| Nottinghamshire | 37.2% | 36.1% | 38.3% | 28.6% | 26.7% | 30.6% |
| Rutland | 37.2% | 32.7% | 41.8% | 28.8% | 20.7% | 36.9% |
| West Northamptonshire | 37.0% | 35.2% | 38.8% | 28.5% | 25.5% | 31.5% |
| **West Midlands** |  |  |  |  |  |  |
| Birmingham | 39.9% | 38.7% | 41.2% | 33.9% | 31.6% | 36.3% |
| Coventry | 39.9% | 37.7% | 42.0% | 33.9% | 29.9% | 38.0% |
| Dudley | 39.8% | 37.9% | 41.7% | 34.0% | 30.4% | 37.5% |
| Herefordshire, County of | 39.6% | 37.4% | 41.8% | 33.9% | 29.8% | 38.0% |
| Sandwell | 39.8% | 37.7% | 42.0% | 33.9% | 29.9% | 37.9% |
| Shropshire | 39.7% | 38.0% | 41.4% | 33.9% | 30.8% | 37.0% |
| Solihull | 40.0% | 37.8% | 42.3% | 34.1% | 29.9% | 38.3% |
| Staffordshire | 39.6% | 38.5% | 40.7% | 33.9% | 31.8% | 35.9% |
| Stoke-on-Trent | 39.6% | 37.3% | 42.0% | 33.8% | 29.5% | 38.0% |
| Telford and Wrekin | 39.4% | 36.7% | 42.2% | 33.7% | 28.8% | 38.7% |
| Walsall | 39.9% | 37.7% | 42.1% | 34.0% | 29.9% | 38.0% |
| Warwickshire | 39.7% | 38.4% | 41.1% | 33.9% | 31.4% | 36.5% |
| Wolverhampton | 40.0% | 37.7% | 42.3% | 34.0% | 29.7% | 38.3% |
| Worcestershire | 39.7% | 38.4% | 41.0% | 33.9% | 31.5% | 36.3% |
| **East of England** |  |  |  |  |  |  |
| Bedford | 37.6% | 34.9% | 40.3% | 31.9% | 27.4% | 36.5% |
| Cambridgeshire | 37.4% | 36.1% | 38.8% | 31.9% | 29.6% | 34.2% |
| Central Bedfordshire | 37.3% | 35.2% | 39.4% | 31.8% | 28.3% | 35.3% |
| Essex | 37.6% | 36.7% | 38.5% | 32.0% | 30.5% | 33.4% |
| Hertfordshire | 37.8% | 36.8% | 38.8% | 32.0% | 30.2% | 33.8% |
| Luton | 37.6% | 34.6% | 40.5% | 31.9% | 26.9% | 36.9% |
| Norfolk | 37.5% | 36.5% | 38.5% | 31.9% | 30.2% | 33.7% |
| Peterborough | 37.5% | 34.8% | 40.3% | 31.9% | 27.2% | 36.5% |
| Southend-on-Sea | 37.8% | 35.2% | 40.4% | 32.0% | 27.6% | 36.5% |
| Suffolk | 37.5% | 36.4% | 38.6% | 32.0% | 30.0% | 33.9% |
| Thurrock | 37.4% | 34.2% | 40.6% | 31.7% | 26.4% | 37.0% |
| **London** |  |  |  |  |  |  |
| Barking and Dagenham | 42.0% | 38.9% | 45.2% | 36.7% | 30.4% | 42.9% |
| Barnet | 42.3% | 40.5% | 44.1% | 36.9% | 33.3% | 40.6% |
| Bexley | 42.4% | 40.3% | 44.5% | 37.0% | 32.7% | 41.4% |
| Brent | 42.1% | 39.9% | 44.2% | 36.8% | 32.5% | 41.2% |
| Bromley | 42.3% | 40.6% | 44.1% | 37.0% | 33.4% | 40.7% |
| Camden | 42.0% | 39.2% | 44.7% | 36.8% | 31.3% | 42.3% |
| City of London | 40.6% | 28.2% | 52.9% | 36.0% | 12.6% | 59.4% |
| Croydon | 42.1% | 40.2% | 43.9% | 36.8% | 33.1% | 40.6% |
| Ealing | 41.8% | 39.8% | 43.9% | 36.7% | 32.6% | 40.7% |
| Enfield | 42.2% | 40.2% | 44.2% | 36.9% | 32.9% | 41.0% |
| Greenwich | 41.7% | 39.2% | 44.2% | 36.5% | 31.6% | 41.5% |
| Hackney | 41.6% | 38.4% | 44.7% | 36.5% | 30.5% | 42.5% |
| Hammersmith and Fulham | 41.9% | 38.7% | 45.0% | 36.8% | 30.5% | 43.0% |
| Haringey | 41.8% | 39.2% | 44.5% | 36.7% | 31.5% | 41.8% |
| Harrow | 42.1% | 40.0% | 44.2% | 36.9% | 32.6% | 41.3% |
| Havering | 42.4% | 40.4% | 44.4% | 37.0% | 32.9% | 41.1% |
| Hillingdon | 42.2% | 40.1% | 44.3% | 36.9% | 32.6% | 41.1% |
| Hounslow | 41.7% | 39.3% | 44.1% | 36.6% | 31.9% | 41.3% |
| Islington | 41.7% | 38.5% | 44.8% | 36.5% | 30.4% | 42.6% |
| Kensington and Chelsea | 41.9% | 38.9% | 44.9% | 36.8% | 30.8% | 42.8% |
| Kingston upon Thames | 42.0% | 39.3% | 44.7% | 36.8% | 31.2% | 42.3% |
| Lambeth | 41.8% | 39.2% | 44.5% | 36.6% | 31.4% | 41.7% |
| Lewisham | 42.1% | 39.5% | 44.6% | 36.7% | 31.7% | 41.8% |
| Merton | 42.2% | 39.6% | 44.8% | 36.8% | 31.5% | 42.1% |
| Newham | 41.5% | 38.7% | 44.3% | 36.4% | 31.1% | 41.8% |
| Redbridge | 42.0% | 39.8% | 44.2% | 36.8% | 32.3% | 41.2% |
| Richmond upon Thames | 41.9% | 39.4% | 44.3% | 36.7% | 31.9% | 41.6% |
| Southwark | 41.8% | 39.0% | 44.5% | 36.5% | 31.2% | 41.7% |
| Sutton | 42.3% | 39.9% | 44.7% | 36.9% | 32.0% | 41.8% |
| Tower Hamlets | 41.5% | 38.3% | 44.8% | 36.4% | 30.0% | 42.7% |
| Waltham Forest | 41.8% | 39.3% | 44.4% | 36.7% | 31.6% | 41.8% |
| Wandsworth | 42.1% | 39.7% | 44.5% | 36.8% | 32.0% | 41.7% |
| Westminster | 41.9% | 39.2% | 44.6% | 36.7% | 31.3% | 42.2% |
| **South East** |  |  |  |  |  |  |
| Bracknell Forest | 37.6% | 33.9% | 41.3% | 30.6% | 24.6% | 36.5% |
| Brighton and Hove | 37.8% | 35.2% | 40.4% | 30.6% | 26.4% | 34.8% |
| Buckinghamshire | 37.9% | 36.4% | 39.5% | 30.8% | 28.3% | 33.4% |
| East Sussex | 38.0% | 36.7% | 39.3% | 30.9% | 28.7% | 33.1% |
| Hampshire | 37.9% | 37.0% | 38.8% | 30.8% | 29.3% | 32.3% |
| Isle of Wight | 37.7% | 35.1% | 40.2% | 30.7% | 26.6% | 34.9% |
| Kent | 37.8% | 36.9% | 38.7% | 30.8% | 29.3% | 32.2% |
| Medway | 37.5% | 35.1% | 40.0% | 30.6% | 26.7% | 34.5% |
| Milton Keynes | 37.4% | 34.8% | 40.0% | 30.5% | 26.3% | 34.6% |
| Oxfordshire | 37.9% | 36.5% | 39.3% | 30.8% | 28.5% | 33.1% |
| Portsmouth | 37.8% | 34.9% | 40.8% | 30.7% | 25.9% | 35.4% |
| Reading | 37.8% | 34.3% | 41.2% | 30.7% | 25.0% | 36.4% |
| Slough | 37.4% | 33.2% | 41.6% | 30.5% | 23.8% | 37.1% |
| Southampton | 37.8% | 35.0% | 40.6% | 30.6% | 26.1% | 35.2% |
| Surrey | 38.1% | 37.1% | 39.2% | 30.9% | 29.2% | 32.7% |
| West Berkshire | 37.5% | 34.6% | 40.4% | 30.6% | 25.9% | 35.3% |
| West Sussex | 38.1% | 36.9% | 39.2% | 30.9% | 29.1% | 32.8% |
| Windsor and Maidenhead | 37.9% | 35.0% | 40.9% | 30.8% | 25.9% | 35.8% |
| Wokingham | 37.8% | 35.0% | 40.7% | 30.8% | 26.1% | 35.5% |
| **South West** |  |  |  |  |  |  |
| Bath and North East Somerset | 36.7% | 34.2% | 39.3% | 30.2% | 25.8% | 34.5% |
| Bournemouth, Christchurch and Poole | 36.8% | 35.2% | 38.5% | 30.2% | 27.4% | 33.1% |
| Bristol, City of | 36.6% | 34.6% | 38.7% | 30.0% | 26.6% | 33.4% |
| Cornwall | 36.4% | 35.0% | 37.7% | 30.0% | 27.8% | 32.2% |
| Devon | 36.6% | 35.5% | 37.7% | 30.1% | 28.3% | 31.9% |
| Dorset | 36.6% | 35.2% | 38.1% | 30.2% | 27.7% | 32.7% |
| Gloucestershire | 36.6% | 35.3% | 37.9% | 30.1% | 27.9% | 32.3% |
| Isles of Scilly | 36.8% | 17.3% | 56.3% | 30.2% | -3.8% | 64.1% |
| North Somerset | 36.7% | 34.5% | 38.8% | 30.2% | 26.5% | 33.8% |
| Plymouth | 36.5% | 34.3% | 38.8% | 30.0% | 26.2% | 33.8% |
| Somerset | 36.6% | 35.2% | 37.9% | 30.1% | 27.8% | 32.3% |
| South Gloucestershire | 36.7% | 34.6% | 38.8% | 30.2% | 26.6% | 33.7% |
| Swindon | 36.4% | 33.8% | 39.0% | 29.9% | 25.6% | 34.2% |
| Torbay | 36.7% | 34.1% | 39.2% | 30.1% | 25.7% | 34.5% |
| Wiltshire | 36.6% | 35.1% | 38.0% | 30.1% | 27.6% | 32.6% |

*Notes:* CI, confidence intervals.
